# Understanding the epidemiological HIV risk factors and underlying risk context for youth residing in or originating from the Middle East and North Africa (MENA) region: A scoping review of the literature

**DOI:** 10.1371/journal.pone.0260935

**Published:** 2022-01-07

**Authors:** Roula Kteily-Hawa, Aceel Christina Hawa, David Gogolishvili, Mohammad Al Akel, Nicole Andruszkiewicz, Haran Vijayanathan, Mona Loutfy

**Affiliations:** 1 Family Studies and Human Development Department, School of Behavioural and Social Sciences, Brescia University College at Western University, London, Ontario, Canada; 2 Ontario HIV Treatment Network, Toronto, Ontario, Canada; 3 School of Medicine, Faculty of Health Sciences, Queen’s University, Kingston, Ontario, Canada; 4 Alliance for South Asian AIDS Prevention, Toronto, Ontario, Canada; 5 Women’s College Research Institute, Women’s College Hospital, Toronto, Ontario, Canada; 6 Faculty of Medicine, University of Toronto, Toronto, Ontario, Canada; International AIDS Vaccine Initiative, UNITED STATES

## Abstract

**Introduction:**

HIV is the second leading cause of death among young people globally, and adolescents are the only group where HIV mortality is not declining. Middle East and North Africa (MENA) is one of few regions seeing rapid increase of HIV infections (31.0%) since 2001. MENA youth are at particular risk of HIV due to dearth of research and challenges in accessing services.

**Objective:**

The purpose of this scoping review is to establish the epidemiological HIV risk factors and underlying risk context for youth residing in or originating from the MENA region.

**Methods:**

Online database searches were conducted using combination of search terms. Screening 5,853 citations, published between 1990–2019 with age groups 16 to 29, resulted in 57 studies included across 18 MENA countries.

**Results:**

‘Key populations’ engage in risky behaviors, including: overlapping risky behaviors among youth who inject drugs (PWID); lack of access to HIV testing, condomless sex, and multiple sex partners among young men who have sex with men (MSM); and high and overlapping risk behaviors among young sex workers. Challenges facing other youth groups and bridging populations include: peer pressure, inhibition about discussing sexual health, lack of credible sex education sources, low condom use, and lack of access to HIV protection/prevention services, especially testing.

**Conclusion:**

Poor surveillance coupled with scarcity of rigorous studies limit what is known about epidemiology of HIV among youth in MENA. Homophobia, stigma around PWID, and illegal status of sex work promote non-disclosure of risk behaviors among youth and curtail serving this population.

## Introduction

Young people and adolescents are disproportionately impacted by the HIV epidemic globally. Indeed, as of 2019, 1.6 million people aged between 10 and 19 years are living with HIV and 190,000 are newly infected [1]. In fact, HIV is the second leading cause of death among young people globally, and adolescents are the only group for which mortality from HIV is not declining [2].

With a steadily growing youth population, the Middle East and North Africa (MENA), which we define to include 18 countries with common socio-historical, linguistic and religious characteristics, is home to 80 million youth. Its young adult population make up 10% of the world’s population [3, 4]. Despite having the lowest HIV prevalence in the world (less than 0.1%), the Middle East is one of few regions where the HIV epidemic has not waned and in fact, is increasing at a rapid rate; with a 31.0% increase of HIV infections since 2001, it has seen the highest documented increase among all regions in the world [5]. In part due to poor access to antiretroviral treatment (ART) as well as the significant presence of homophobia and discrimination based on sexual orientation and gender identity, AIDS-related deaths increased in the region by 66.0% between 2005 and 2013 at a time when global AIDS-related deaths fell by 33.0% [6].

Mirroring global HIV transmission patterns–with the exception of Sub-Saharan Africa where generalized population epidemics are predominant–the HIV epidemic in MENA has primarily affected people who inject drugs (PWIDs), men who have sex with men (MSM), and female sex workers (FSWs), commonly referred to as ‘key populations’. Additionally, other groups–known as ‘bridging populations’ have been impacted–albeit to a much lesser extent compared to key populations. These groups, such as clients of sex workers–are commonly behind the transmission of HIV/STIs from high-risk core groups to noncore groups, linking key populations with the general population [7].

Notwithstanding the heterogeneity in risk behaviors and risk contexts that characterize the MENA region, two predominant patterns can be noted with respect to the spread of HIV across the region. On one hand, there are countries where HIV prevalence is substantial; where a state of generalized HIV epidemics–defined as HIV rates consistently greater than 1% among pregnant women–applies to parts of Somalia, Djibouti and Southern Sudan [8]. In these contexts, HIV infections are most common among key and bridging populations. The second group are countries with a low HIV prevalence, and these represent the majority of MENA countries [3, 8]. In the latter group, concentrated epidemics–defined as an epidemic with an HIV prevalence greater than 5.0% in a key population–have been documented in several countries. For example, there is evidence for concentrated epidemics among PWIDs in some MENA countries, and emerging evidence for potential concentrated epidemics among MSM. In this group of countries, however, there is no evidence for concentrated epidemics among FSWs or epidemics in the general population [3, 8].

Little is known about the epidemiology of the HIV epidemic in the region. Poor surveillance coupled with a dearth of rigorous studies in the scientific literature limit what is known about the epidemiology of HIV. Illustratively, a study assessing HIV surveillance systems in the region has found that only four of 23 MENA countries had proper systems to enable them to estimate patterns of HIV epidemics [9]. Moreover, perceptions of HIV through a ‘prism of sin’, including significant homophobia, have promoted non-disclosure of risk behaviors and curtailed the emergence of organizations to serve populations at risk [10].

Against this backdrop, a review of the literature on HIV among young populations in MENA is timely. While past reviews have examined the epidemiology of HIV in different key populations in the region [11, 12], there is no comprehensive literature review that has synthesized information on risk behaviors of young people specifically. The purpose of this scoping review is to establish the epidemiological HIV risk factors and underlying risk context for youth residing in or originating from MENA. Such undertaking should help shed light on existing gaps in the literature and provide evidence to inform the development of interventions targeting this group.

## Methodology

Our review of the literature was guided by the scoping review methodological framework developed by Arksey & O’Malley [13]. We used PRISMA Extension for Scoping Reviews (PRISMA-ScR): Checklist and Explanation in developing our protocol [14]. The final protocol is available on request from the corresponding author. The purpose of scoping reviews is to characterize the breadth of the existing literature on a given topic and as such, they lend themselves to broad research questions [15]. Our research question was thus broadly identified as “what is the volume and scope of available information on risk behaviors of youth from the MENA region and what are the research gaps in the current evidence-base?”

### Search strategy

To identify potentially relevant documents, the following bibliographic databases were searched from 1990 to September 10, 2019: MEDLINE, EMBASE, PsycINFO, CINAHL, Web of Science, Cochrane Library. The search strategies were drafted by an experienced team member [DG] and further refined through team discussion. The final search strategy for Medline can be found in S1 Table.

The final search results were exported into EndNote, and duplicates were removed. The electronic database search was supplemented by Google Advanced Searches on November 12^th^, 2019, using combinations of search terms HIV and (youth or young or student or adolescent) with MENA country names. First author and Principal Investigator [RKH] along with senior author [ML] screened the first 200 results of each of these Google Advanced searches. To maximize identification of relevant articles, we additionally reviewed reference lists of select studies and systematic reviews.

### Inclusion/exclusion criteria

To be included in the review, articles needed to address the issue of HIV risk among youth residing in or originating from the MENA region. For the purpose of this scoping review, MENA was defined as the following: Algeria, Bahrain, Egypt, Iran, Iraq, Jordan, Kuwait, Lebanon, Libya, Morocco, Oman, Palestine, Qatar, Saudi Arabia, Syria, Tunisia, United Arab Emirates (UAE), and Yemen. Peer-reviewed journal articles as well as published reports and conference abstracts were included if they were: published between the period of 1990–2019, written in English, French or Arabic; and provide a separate analysis for participants in the age group 16 to 29. Studies were excluded if they focused on one of the following (unless related to HIV/STI risk): incidence or prevalence of a disease; clinical trials; HIV/STI treatment regimens; drug use; sexual abuse, coercion, harassment, violence; clinical investigations; sexual function; mother to child transmission of HIV; cost effectiveness or cost assessment.

### Article selection

A team member [DG] imported articles into DistillerSR (Evidence Partners, Ottawa, Canada) and two reviewers [RKH] [AH] screened 4283 titles and abstracts of all publications identified in the first stage of the review, and selected articles for full-text review. Two reviewers [MA] and [NA] independently reviewed the retrieved full-text articles to assess their eligibility for inclusion in the review. Where conflicts or discrepancies arose in the study selection, disagreements were resolved through discussion between the two reviewers or further adjudication by a third reviewer [RKH].

### Charting the data

Two team members [RKH] [DG] charted the data using a data-charting tool which they developed jointly. Team members [MA] [NA] abstracted the data and discussed the results and continuously updated the data charting form iteratively with input from team members [RKH] [AH] [DG] [ML]. Data was abstracted on a variety of study characteristics such as: study jurisdiction; publication type (peer-reviewed journal, report, conference abstract); number of participants within our age range of interest (i.e. 16–29 years); age and sex of participants; outcome definition used in the study; factors associated with risk behaviors; main findings related to the HIV/STI risk behavior.

### Data synthesis and analysis

We did not assess risk of bias or appraise quality of the articles, per guidance on scoping reviews [13]. We organized our analysis by population groups, emphasizing the key populations at risk of HIV and other groups most frequently featured in the literature such as prisoners, truck drivers, and street children. When available, we presented estimates for risk behaviors that were specific to the age range of interest.

## Results

**Fig 1:**
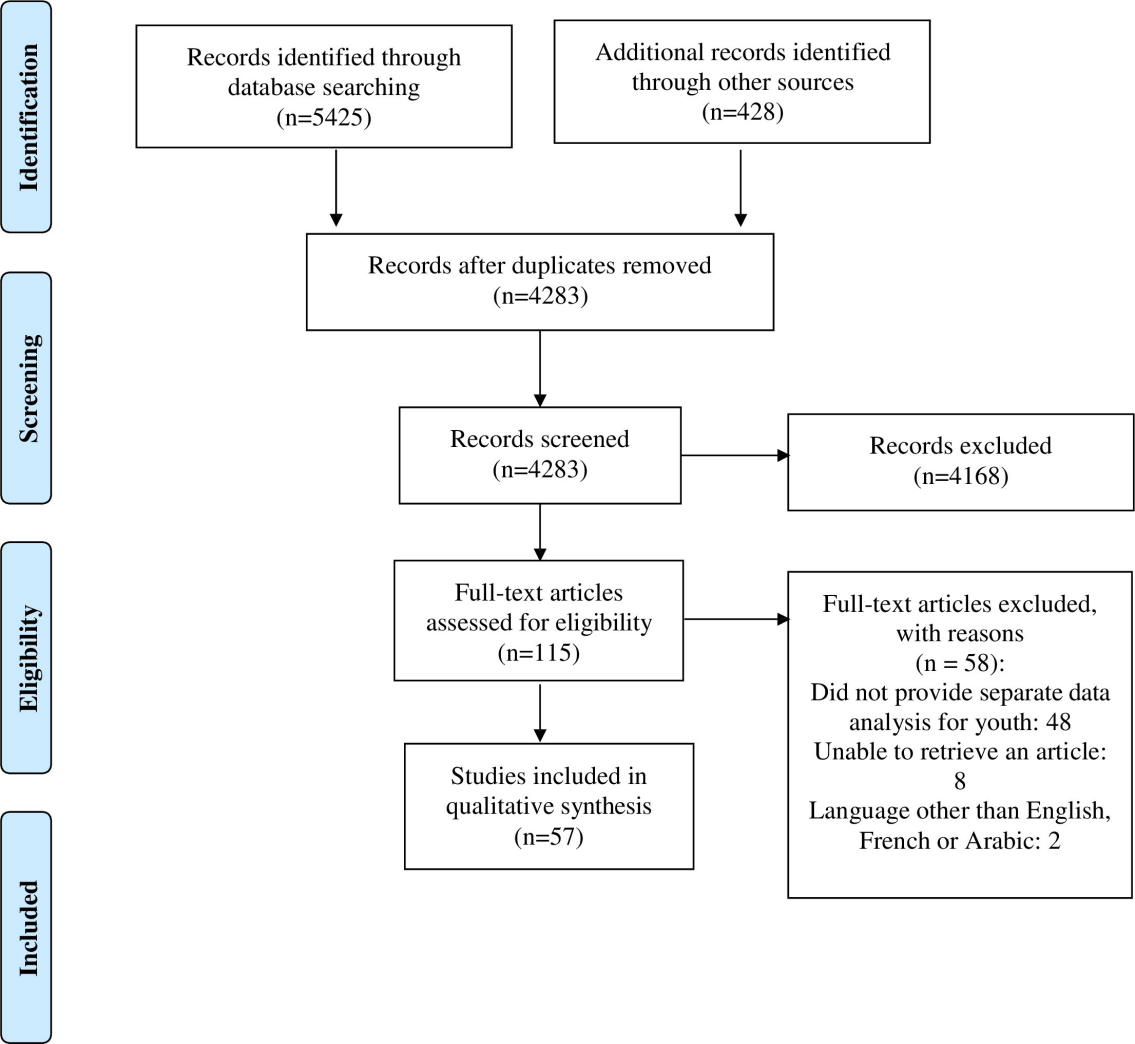
PRISMA flow diagram.

The search strategy identified 5,853 citations (Fig 1). After removal of 1,570 duplicates, 4,283 citations were screened by title and abstract. Of these, 4,168 did not meet the criteria for inclusion, with 115 full text articles to be retrieved and assessed for eligibility. Of these, 58 were excluded for these reasons: 48 did not provide separate data analysis for youth (age range 16–29), two were in Persian language, and we were unable to retrieve eight full text articles (Fig 1).

The remaining 57 studies were considered eligible for this review. These were 52 peer-reviewed journal articles four reports which mainly covered HIV risk behaviors among key and bridging populations, and one conference abstract (Table 1).

**Table 1:** Characteristics of research studies included in the scoping review.

Authors	Setting	Sample Size	Data Collection Dates	Population	Study Design	Main Findings
**Adib et al. 2002**	Beirut, Lebanon	730	1999	Male conscripts	Quantitative Cross-sectional	Urban residence, higher education, lower family crowding, and young age at first sex were factors of higher sexual risk recommended to be reduced by epidemiological and behavioral surveillance, and educational programs.
**Hendrickx et al. 2002**	Antwerp, Belgium	55	1997–1998	Adolescent boys and girls	Qualitative using FGDs*	Adolescents have limited knowledge of contraceptive and AIDS. Most boys show no concern about risks for future virgin spouses while most girls do not consider safe sex before or after marriage.
**Refaat et al. 2004**	Ismailia, Egypt	687	Not reported	University Students	Quantitative Cross-sectional	18% of students practiced risky behaviours positively correlated with tobacco use, alcohol and drug use, and risky sexual behaviour. 30% of students lacked knowledge on AIDS. Knowledge sources were the media (38%) then peers (30%).
**Hajiabdolbaghi et al. 2006**	Tehran, Iran	33	Not reported	Runaway and at-risk women	Quantitative Pre/Post test	Half were less than 24; HIV knowledge was high. Few using illicit substances said used “frequently.” Nonresponse rates to questions regarding sexual behavior were high. Half admitted a history of sexual activity; 40% of those reported their first sexual contact with someone other than their husband; three people had multiple partners. Three women reported a history of rape. Zero women tested HIV positive.
**Mohammad et al. 2007**	Tehran, Iran	382	2002	Adolescent boys	Quantitative Cross-sectional	Older age, alcohol usage, early sexual debut, and poor knowledge of reproductive physiology are predictors of multiple sexual partners among adolescent boys aged 15–18 years.
**El-Sayyed et al. 2008**	Cairo, Egypt	58	2003	MSM	Quantitative Cross-sectional	65.8% initiated sexual activity before 15 years; 65.8% took both active and passive roles in sexual acts. Heterosexual relations were reported by 73.3% of the older age group. 70.7% of the younger age group were exclusively MSM. 19.2% of the sample used condoms.
**El-Sayyed et al. 2008**	Mahalla El-Koubra, 10th of Ramadan and Kafr El- Dawar, Luxor city, South Sinai, Egypt	1170	Not reported	Industrial and tourist workers	Quantitative Cross-sectional	Compared with industrial workers, tourism workers had a significantly better perception of the magnitude of the HIV/AID, its causative agent, and its methods of transmission.
**Tehrani et al. 2008**	Saravan, Astara, Islamshahr and Kermanshah, Iran	754	2003	Truck drivers, female sex workers, and general population	Quantitative Cross-sectional	The level of knowledge about HIV was low on average, especially among individuals with high-risk behaviours. Condom use was low in all groups. Better targeted education of high-risk groups is needed.
**Kahhaleh et al. 2009**	Beirut, Lebanon	2064	2004	General Population	Quantitative Cross-sectional	Of sexually active respondents, 13.0% of men and 2.6% of women had regular partners other than the spouse but only 25.0% used a condom in their last sexual intercourse. 16.8% had sex with casual partners, 71.7% of those used condoms.
**Al-Iryani et al. 2010**	Aden, Yemen	Not reported but majority are youth	2005–2009	Not reported	Qualitative using FGDs* and IDIs**	The evaluation highlights enabling factors that facilitated the implementation of a community peer education program for youth HIV prevention in four areas of Aden, Yemen.
**Nada et al. 2010**	Greater Cairo and Alexandria, Egypt	443	2007	Street Children	Quantitative Cross-sectional	Among the sexually active 15–17-year-olds, 54% reported multiple partners and 52% never using condoms. Most girls had experienced sexual abuse. Most street children experienced more than one of these risks. Populations at highest risk for HIV were MSM, commercial sex workers, and injection drug users.
**Hosseini et al. 2010**	Tehran, Iran	271	2006	IDU	Quantitative Cross-sectional	No association between other demographic characteristics, type and years of drug abuse, age of first injection, years of injection, sharing needles inside and outside of jail, injection in jail, history of tattooing, any sexual behavior, and history of sexually transmitted diseases with HIV/HCV coinfection.
**Mahfoud et al. 2010**	Beirut, Lebanon	143	2007–2008	FSW, MSM, IDU	Quantitative Cross-sectional	MSM HIV prevalence was 3.7% but no HIV cases were detected among female sex workers or IDUs. Three- quarters of MSM had nonregular male sexual partners during the last year but only 39% reported using a condom. 36% of MSM and 12% of IDUs reported sold sex.
**FHI/MOH Egypt, 2010**	Cairo and Alexandria, Egypt	Not reported	2010	FSWs, street children, MSM, and Male IDUs	Quantitative Cross-sectional	Overlapping of risk behaviors including commercial sex, injecting drugs and MSM activity is a common finding among different groups of MARPs. MSM Luxor didn’t harbor the HIV infection.
**Kingdom of Morocco, 2010**	Morocco	2000	Not reported	General Population	Quantitative Cross-sectional	Differentials in knowledge putting women, rural populations, refugees, and other marginalised minorities at a disadvantage. Attitudes towards PLHIV tended to be negative.
**Sayarifard et al. 2011**	Tehran, Iran	Not reported	2009	FSW	Quantitative Cross-sectional	Mean age of participants was 26.8. 51 (39.8%) of the female sex workers said they never had anal intercourse, 13 (10.1%) reported sometimes, only 8 (6.3%) of whom always used condom and 56 (43.8%) never used condom.
**Mirzaee et al. 2012**	Iran	495	2013	General Population	Quantitative Cross-sectional	The differences for inconsistent condom use, were significant (P<0.05): age, gender, knowledge of HIV, attitude towards HIV, knowing infected HIV person and alcohol or stimulant(s) used before sexual contact.
**Shokoohi et al. 2012**	Kerman, Iran	333	Not reported	IDUs, MSM, and males who have sex with FSW	Quantitative Cross-sectional	13.7% of males used alcohol at least once in last year; the percent for opium was 13.1%. 12% had extra-marital sex in last year; 7% had sex with a female sex worker.
**Wagner et al. 2012**	Beirut, Lebanon	31	2011	MSM	Mixed methods	Sixty-four percent reported unprotected anal intercourse (UAI), including 23% who had UAI with unknown HIV status partners (UAIU); 62% tested for HIV.
**Ismael et al. 2012**	Erbil, Iraq	333	2010–2011	General Population	Quantitative Cross-sectional	The main reason to non-condom use was lack of need in 45.5%, fertility related reasons in 17% and the use of other methods by the female partner 13.6%. 64% of respondents heard about AIDS /HIV and 71.7% about STIS, few felt at risk of STIs 9.5% and HIV infection 8.5%.
**Sajadi et al. 2013**	Iran	210	2010	FSW	Quantitative Cross-sectional	The frequencies of condom use in the last sexual act with paying clients and non-paying partners were 57.1% and 36.3%, respectively. Any drug use was reported by 73.8% of participants, 20.5% had a history of injection drug use.
**Khajehkazemi et al. 2013**	Iran	318	2010	IUD	Quantitative Cross-sectional	Among those who had injected drugs over the last month, 36.9% had used a non-sterile needle, and 12.6% had practiced shared injection. Over the past 12 months, 30.4% had sold sex for money, drugs, goods or a favour.
**Valadez et al. 2013**	Tripoli, Libya	241	2010–2011	MSM and FSW	Quantitative Cross-sectional	MSM HIV prevalence estimated at 3.1% and FSW HIV prevalence of 15.7%. We detected high levels of risk behaviours, poor HIV-related knowledge, high stigma and lack of prevention programmes.
**Mirzazadeh et al. 2013**	Tehran, Iran	15	2011	FSW	Qualitative using IDIs**	FSW significantly under-reported number of clients, sexual contacts and non-condom use sex acts with clients and number of days engaging in sex work in the preceding week.
**Johnston et al. 2013**	Agadir and Marrakesh, Morocco	463	2010–2011	MSM	Quantitative Cross-sectional	Most MSM in both cities reported being < 25 years, being unemployed, bisexual and in a couple with both a man and a woman. Most reported selling sex and having sex with women.
**Shayestehkhou et al. 2013**	Tehran, Iran	30	Not reported	Transwomen	Quantitative Cross-sectional	Although frequency of sexual risk behaviors did not change significantly in experimental group, higher sexual risk behaviors were reduced significantly (P≤0.01).
**Wagner et al. 2014**	Beirut, Lebanon	100	2012	MSM	Quantitative Cross-sectional	Mean age of the participants was 28.4 years, 77% identified as gay and 33% as bisexual. Half reported not using condoms consistently and one quarter had not been HIV-tested.
**Hedayati-Moghaddam et al. 2014**	Mashahd, Iran	605	2008	University Students	Quantitative Cross-sectional	The mean age of first sexual experience was 23.7. In single sexually experienced students, the mean age at first sex was 17.6±3.3 years, 24% started sexual activity at <15 years, 34.3% had at least 3 partners and only 40.6% used condom in their last sex.
**Massad et al. 2014**	Jerusalem, Palestine	83	Not reported	General Population	Qualitative FGDs* and IDIs**	Youth engage in sexual activity outside marriage to challenge the culture, financial constraints and inability to marry, basic human need, personal pleasure, suppression, to kill boredom, and to prove manhood.
**Ghandour et al. 2014**	Beirut, Lebanon	983	2012	University Students	Quantitative Cross sectional	Students who used alcohol/drugs at sexual debut were twice as likely to have: their first oral and vaginal sex with an unfamiliar partner, controlling for sex, nationality, current relationship status, living abroad after the age of 12, and spirituality.
**Alkaiyat et al. 2014**	Amman, Aqaba, Irbid and Zarqa, Jordan	73	2011	MSM	Quantitative Cross-sectional	Positive determinants of condom use were higher education level, acknowledging MSM as a high-risk group, seeking advice from a medical doctor and the perceived causes ‘‘sex with prostitutes” and ‘‘sex with animals.”
**Mirzazadeh et al. 2014**	Aden and Al-Hudaydah, Yemen	166	2011	MSM	Quantitative Cross-sectional	25.8% tested for HIV in the last year and received results; 27.8% had comprehensive knowledge about HIV; 20.0% reported condom use at last anal sex; and 31.4% reported they or their sexual partner had a sexually transmitted disease symptom.
**Kobeissi et al. 2014**	Suburbs of Damascus (Rif Damascus), Lattakia and Tartous, Syria	Not reported	2013–2014	FSWs, MSM, IDUs and Prisoners	Quantitative Cross-sectional	For all four groups, the knowledge of the symptoms was slightly better when identifying women-related symptoms compared to men. Adequate knowledge was less than 30% in both categories.
**Noroozi et al. 2015**	Isfahan, Iran	30	2012–2013	General Population	Qualitative using IDIs**	Women seldom asked for condom use due to limited knowledge about STIs-HIV/AIDS and unpleasant experiences with condoms. Men had limited knowledge about HIV and STIs transmission and did not use condoms consistently because they had not seen themselves at risk of STIs or HIV and their belief of decreased sexual pleasure.
**Esmaeilzadeh et al. 2015**	Jolfa, Iran	156	2013	University Students	Quantitative Cross-sectional	Low self-control trait and low perceived susceptibility significantly were related to having a history of multi-sex partners while high level of self-efficacy significantly increased the probability of condom use.
**Salehi et al. 2015**	Shiraz, Iran	825	2006–2011	Sex workers and IDUs	Retrospective Record Review	Shared injection, history of imprisonment, maleness, unsafe sex, inadequate housing, and low education were risk factors for HIV infection. A history of imprisonment and substance use were significant risk factors for female sex workers.
**Wagner et al. 2015**	Beirut, Lebanon	100	2012	MSM	Quantitative Cross-sectional	47% were under 25 years and 67% self-identified as gay. 64% reported any unprotected anal intercourse (UAI) with men in the prior 3 months, including 23% who had unprotected anal intercourse with men whose HIV status was positive or unknown (UAIPU).
**Aunon et al. 2015**	Beirut, Lebanon	16	2011	Male sex workers	Qualitative using IDIs**	The uptake of HIV testing was limited by concerns about the confidentiality of the test results and fear of repercussions of a positive test result for health and employment.
**Shokoohi et al. 2016**	13 provinces in Iran	4950	2013	General Population	Quantitative Cross-sectional	37.3% of the participants had a high knowledge score. Misconceptions existed about HIV transmission through mosquito bites across all age groups (31.7% correct response). Positive levels of attitude were observed in 20.7% of the participants.
**Kaplan et al. 2016**	Beirut, Lebanon	40	2012	Transwomen	Quantitative Cross-sectional	Fifty-seven percent of participants reported condomless receptive anal intercourse (CRAI) with male partner(s) in the last three months, 40% reported not knowing the HIV status of the partner(s).
**Maarefvand et al. 2016**	Tehran, Iran	114	2014	Truck drivers	Quantitative Cross-sectional	Younger LDTDs reported more condom use with their partners, more extramarital sexual contacts, more pay for sex and condom use in their extramarital sex contacts.
**Maatouk et al. 2016**	Beirut, Lebanon	28	2015	MSM	Quantitative Cross-sectional	In Lebanon, men who have sex with men may account for most new human immunodeficiency virus (HIV) infections. The proportion of people infected with HIV among men who have sex with men in 2011 is estimated at 3.6%.
**Honarvar et al. 2016**	Shiraz, Southern Iran	935	2012–2013	General Population	Quantitative Cross-sectional	1076 participants (634 males, 58.9%) with a mean age of 24±5.8 years participated. In the regression analysis, alcohol use was the strongest associated factor of PMS followed by lack of religious beliefs
**Abdel-Tawab et al. 2016**	Egypt	3733	Not reported	General Population	Qualitative using FGDs* and IDIs**	Adolescents (age 15–19), young men and women with no education and those in the lowest wealth quintile are particularly vulnerable to HIV infection because of their limited knowledge and access to information.
**Sharifi et al. 2017**	Kermanshah, Iran	220	2014	IDUs	Quantitative Cross-sectional	Focused on multiple HIV risk. Compared to members in the lowest-risk class, the highest-risk class members had higher odds of being homeless in the past 12 months. Members of the high-risk class had lower odds of regularly visiting a needle and syringe exchange program as compared to the lowest-risk class members.
**Heimer et al. 2017**	Beirut, Lebanon	219	2014–2015	MSM	Quantitative Cross-sectional	HIV prevalence increased over past estimates. Efforts to control future increases will have to focus on reducing specific risk behaviors and experience of stigma and abuse, especially among Syrian refugees.
El Kak et al. 2017	Beirut, Lebanon	2180	Not reported	University Students	Quantitative Cross-sectional	Not communicating with partners was associated with increased odds of not knowing about the effectiveness of condoms at preventing pregnancy and misperceiving that birth control pills are effective at preventing HIV/AIDS.
**Hooshyar et al. 2018**	Iran	632	2013	General Population	Quantitative Cross-sectional	Men reported significantly higher condom use than women (38.5% vs. 25.7%). Having a stable job, higher knowledge of condom and sexual transmission of HIV were positively associated with condom use at last sex.
**Elamouri et al. 2018**	Tripoli, Libya	31	2015	Prisoners and youth attending rehab centers and schools	Qualitative using IDIs**	Risk factors for drug use included peer influence, the increased availability and affordability of drugs, disruption of social life and healthy recreational activities, and the distress and casualties of the war.
**Sajjadi et al. 2018**	Tehran and Alborz, Iran	634	2015	University Students	Quantitative Cross-sectional	The variables of age, gender, marital status, type of residence and academic degree were significantly related to the likelihood of having close friends with certain high-risk behaviors
Khalajabadi Farahani et al. 2018	Tehran, Iran	950	2013–2014	University Students	Quantitative Cross-sectional	Younger age at sexual debut, having one lifetime sexual partner and poor HIV knowledge were significant predictors of inconsistent condom use over the preceding month.
**Noroozi et al. 2018**	Kermanshah, Iran	312	2013–2014	IDU	Quantitative Cross-sectional	Compared to PWIDs who reported no sexual risk behavior, participants that were more likely to partake in sexual risk behaviors were those of low or moderate socioeconomic status and methamphetamine use
**Armoon et al. 2018**	Kermanshah, Iran	255	2014	IDU	Quantitative Cross-sectional	Of 433 PWID who participated in this study, 36% reported high HIV risk perception. Methamphetamine use) or use of multiple drugs at the same time were associated with higher HIV risk perception.
**Noroozi et al. 2018**	Tehran, Iran	455	2016	IDU	Quantitative Cross-sectional	The prevalence of receptive sharing, distributive sharing, and inconsistent condom use was 32%, 15% and 55%, respectively.
**Wagner et al. 2019**	Beirut, Lebanon	390	2016–2017	MSM	Quantitative Cross-sectional	Low income was the sole correlate of having recently tested for HIV. These findings suggest a temporal trend toward increased HIV protective behaviors among YMSM in Beirut over the past 5 years.
**El Kazdouh et al. 2019**	Taza, Morocco	56	2016	Adolescent girls and boys	Qualitative using FGDs*	Five overall themes seemed to influence risky sexual behaviors in adolescents: (1) risky sexual practices and STIs; (2) the adolescent’s social domain; (3) the role of school; (4) media, including internet and social media; and (5) socio- cultural norms
**Ghanem et al. 2019**	Beirut, Lebanon	218	2016–2017	MSM	Quantitative Cross-sectional	15% of the sample reported recent condomless anal sex with partners whose HIV status was positive or unknown, and 82.3% had ever been tested for HIV.

*FGD: Focus Group Discussions.

**IDI: In-Depth Interviews.

### Youth who inject drugs

Eleven articles investigated risk behaviors of injection drug users among participants who fell in our age range. Most research studies were conducted in Iran, two were conducted in Lebanon, and one was conducted in Egypt (Table 1). Understanding the social determinants of risky sexual behaviors is important among youth who use drugs. Cross-sectional data analyzed from the HIV Behavioral Surveillance among PWID, conducted from 2013 to 2014 in Kermanshah, Iran with additional data collected via in-depth interviewing, found that PWID who reported having multiple sex partners with inconsistent condom use were more likely to experience poly-drug use, being of low socioeconomic and education level, and refuse to attend harm reduction programs [16]. While there is no exact data on the number of people injecting drugs that fall in the age range 16 to 29, a significant proportion of injection drug users begin using drugs at or before age 25. A study conducted in 2014 in Iran revealed that the average age at first drug use in Kermanshah was 21.4 ±5.6, and duration was 6.0 ±4.6 years [17]. Similarly, a study in Southern Iran found that the mean age of first substance use among a group of people at Drop-in Centers was 20.24 ±6.11 [18]. In a study conducted in Lebanon in 2010, 60.76% of PWIDs stated they were at or below 25 years of age when they started injecting drugs [19].

#### Multiple HIV risks

Youth who inject drugs tended to have a significantly higher probability of having multiple HIV risks compared to their older counterparts. Studies reveal a generally high- risk context, in which young PWIDs engage in multiple risk behaviors such as sharing needles/syringes, using non-sterile injecting equipment, partaking in inconsistent condom use, selling sex for money or favors, and having multiple sexual partners [19–21]. Also, HIV/HCV co-infection was associated in Iran with being divorced, having tattoos and past incarceration history [22].

In a national bio-behavioral surveillance survey conducted in 2013 in Iran, Khajehkazemi et al. [23] found that the most common risk behavior among people who inject drugs was use of a non-sterile needle (37%), followed by selling sex for money (30.4%). Moreover, around 13.0% of the sample had practiced shared injection, and only 38.3% used a condom at last sexual intercourse. In Lebanon, Mahfoud et al. [19] state that one fifth of their sample reported sharing needles during their last injection and half of PWIDs stating they had ever bought sex. With respect to condom use, less than half of the 81 PWIDs surveyed reported using a condom the last time they had sex with either a regular noncommercial sex partner or a non-regular noncommercial sex partner. Similarly, in Egypt, PWIDs practiced multiple risk behaviors, most commonly sharing needles (40.5% in Alexandria and 23.0% in Cairo) and inconsistent condom use with regular noncommercial sex partners (3.0%), and non-regular noncommercial sex partners (30.4%) [24]. While another study in Tehran reported multiple risk behaviors among PWIDs, with inconsistent condom use at 55.0%, and receptive and distributive needle sharing at 32.0% and 15.0% respectively, concomitant alcohol use was found to significantly exacerbate prevalence of risk behaviors [25].

Moreover, there was ample evidence for heterogeneity in overlapping risk among PWIDs. For example, in Lebanon, over half of the 81 PWIDs surveyed stated they had ever bought sex and 12.0% had sold sex [19]. In Iran, about one third of the sample of 2290 PWIDs reported selling sex for money [23].

### Young men who have sex with men

Sixteen articles focused on MSM who fell in our age range. The evidence demonstrates that young MSM in the region engage in risky behaviors including condomless sex, multiple and concurrent sexual partnerships, transactional sex and concurrent injection drug use. Countries such as Lebanon, Egypt, Libya, Jordan, and Syria reported the riskiest behaviors relating to access to HIV testing, number of sexual partners, and frequency of injection drug use (Table 2).

**Table 2:** Risky behaviors among men who have sex with men (MSM) in the MENA region.

	Risky Behaviors Among MSM	
Access to HIV testing	Number of sexual partners	Frequency of injection drug use
	**Lebanon**	
79.6% (Wagner et al. 2018) 94.5% (Heimer et al. 2017) In the past year: 71% undergone at least one test (Maatouk et al. 2016) 75% (Wagner et al. 2012) 22% (Mahfoud et al. 2010) Ever tested: 81.7% (Ghanem et al. 2019) Tested in the last six months: 50.9% (Ghanem et al. 2019) In the past year: 50% (Wagner et al. 2015)	Over the past three months: 2 (Wagner et al. 2014) Over the past year: 20.7 (Heimer et al. 2017) Over the past year 25.3 (Wagner et al. 2012) Over the past year: 73% had at least one nonregular noncommercial sex partner (38% had five or more), and 37% had at least one regular noncommercial sex partner in the last year (1% had five or more) (Mahfoud et al. 2010)	Ever injected drugs: 1.7% (Heimer et al. 2017)
	**Egypt**	
Very few young people undergo HIV testing for fear of facing implications of a positive result and associated social stigma. (Abdel- Tawab et al. 2016)	Per week: < 3 among 48.3% of younger persons and among 40.0% of the older ones (El-Sayyed et al. 2008)	Alexandria (4.9%), Luxor (4.8%) and Cairo (2.3%) (FHI/MOHP Egypt 2010)
	**Libya**	
In the past year: 45.6% (Valadez et al. 2013)		
	**Jordan**	
38% (Alkaiyat et al. 2014)		64.9% (Alkaiyat et al. 2019)
	**Syria**	
31.8% (Kobeissi 2014)		

Lebanon was heavily represented in the evidence-base on MSM risk behaviors in the region with eight out of the 16 articles conducted in Beirut. Studies indicate that MSM are sexually active and engage in multiple risk behaviors at an early age with 52.0% of MSM reporting having initiated sexual activity under the age of 18 [19]. Table 3 describes average age at sexual debut for different population groups.

**Table 3:** Average age at sexual debut among youth population groups in the MENA region.

	Average Age at Sexual Debut
	**Iran**
**Sex Workers**	First commercial sex: 21 years (Mirzazadeh et al. 2013) First commercial sex: 24.6 years (Sajadi et al. 2013)
**University Students**	19 years (Farahani et al. 2018) 23.7 years (Hedayati-Moghaddam et al. 2014)
**General Population**	15 years (Mohammad et al. 2007) Heterosexual contact: 17.9 (Honarvar et al. 2016) Homosexual contact: 16 years (Honarvar et al. 2016) Bisexual contact: 14.7 years (Honarvar et al. 2016) Extramarital sex: 19 years (Shokoohi et al. 2016) Men: 19 years (Hooshyar et al. 2018) Women: 20 years (Hooshyar et al. 2018) Adolescent boys: 14.7 years (Honarvar et al. 2015) Adolescent girls: 19.7 years (Honarvar et al. 2015)
**Bridging Population**	Women at-risk: 22.5 years (Hajiabdolbaghi et al. 2007)
	**Egypt**
**MSM**	The age at which homosexual relations was initiated was < 15 years among 65.8% of respondents (El-Sayyed et al. 2008)
**Bridging Population**	Street children with opposite sex: 14 years (Nada & El Daw 2010) Street children with same sex: 13 years (Nada & El Daw 2010)
	**Syria**
**Sex Workers**	Sexual debut: 17.8 years (Kobeissi 2014) First commercial sex: 21.9 (Kobeissi 2014)
**Bridging Population**	Prisoners: 14.5 years (Kobeissi 2014)
	**Lebanon**
**MSM**	Age at first sex with a man: 16.7 years (Heimer et al. 2017) Age at first sex: 13.9 years (Wagner et al. 2012) Age at first sex with a man: 17 years (Valadez et al. 2013) Age at first sex with a man: 14.9 years (Mirzazadah et al. 2014)
**Sex Workers**	36% of FSWs had sexual intercourse before the age of 16 years and 63% of the FSWs stated they had been under 18 years of age (Mahfoud et al. 2010)
**University Students**	17.75 years (Ghandour et al. 2014)
**General Population**	36.0% first had sexual intercourse at ≤ 20 years (Kahhaleh et al. 2009)
**Bridging Population**	Trans Feminine Individuals: 13 years (Kaplan et al. 2019)

#### Risky behavior

Condomless sex was very common across studies in Lebanon [19, 26–32]. Ghanem et al. [26] found that among the 176 MSM reporting having anal sex, 48.9% did not use condoms during sex in the last three months, and 17.0% of those reported unprotected sex with a partner who had HIV or whose status was unknown [26]. Echoing these findings, Wagner et al. [32] examined trends in condom use among MSM finding that 45.8% of MSM in their 2017 sample reported sex without a condom and 17.3% reported unprotected sex with an HIV-positive or unknown status partner. However, earlier studies suggest that condomless sex was considerably more common in the MSM community compared to more recent studies. For example, Maatouk et al. [27] found that 58.1% of participants reported unprotected anal sex and in another 2014 study, Wagner et al. [30] found that nearly two-thirds (64.0%) of MSM reported any unprotected anal intercourse with men in the prior 3 months, 23.0% of whom reported unprotected anal intercourse with HIV positive or unknown status partners. Examining condom use by type of partner, Mahfoud et al. [19] found that condom use every time they had sex was more common among MSM engaged in sexual intercourse with regular non-commercial partners (63.0%) compared to those with non-regular noncommercial sex partners (39.0%).

Similar patterns were noted in other countries. In a study in Libya, 21.0% of MSM only reported using condoms during their last anal intercourse [33]. Another study in Morocco with 669 MSM revealed that condomless sex with a commercial partner was practiced by 41.0% of MSM and 53.5% reported not using condoms with a regular male sex partner [34]. In Jordan, consistent condom use was very low at 10.0%, only 27.0% reported having used a condom at last intercourse, and almost one fifth of the sample reported never using condoms during sex [35]. Condomless sex was similarly common in Egypt with between the range of 19.2% and 51.0% of MSM in Cairo, 34.4% in Alexandria, and 22.0% in Luxor reporting condom use during commercial sex [36, 37]. In Yemen, condomless anal sex was very common, with 80% reporting not using condoms in their last anal intercourse [38].

#### HIV testing

HIV testing among MSM was variable across samples. In Lebanon, ever having HIV testing ranged from 62.0% to 82.3% [26, 30]. Testing was much lower in Jordan where only 38% of MSM reported ever getting tested and only 60.0% had knowledge that testing was available in the country [35]. In Syria, only 31.8% indicated that they have ever tested for HIV/AIDS, among which only 20.5% were voluntarily tested [39]. In Egypt, HIV testing varied significantly across governorates with 22.1%, 14.5%, and 2.0% of MSM participants in Cairo, Alexandria, and Luxor ever having been tested, respectively [36].

#### MSM with female partners

Across MENA countries, most MSM reported having female sexual partners. In Libya, less than 70.0% of MSM had sex with females through spousal and non-spousal relationships, and in the past six months, around half reported having intercourse with a woman [33]. In Morocco, most participants reported being bisexual and a larger proportion reported having sex with women (81.0% in Agadir and 83.0% in Marrakech). A little less than half of participants reported using condoms with female partners [34]. In Egypt, sex with females was less common at 40.0% in Cairo, but was very high in Alexandria and Luxor, at 59.0% and 87.0%, respectively [36]. In Yemen, sex with females was reported by 40.9% of MSM, who had an average of 4.3 female sexual partners over the last 6 months of which 2 were commercial partners and the remainder were regular or casual partners [38].

#### Overlapping risk

The studies provide evidence of overlapping risk among MSM. One study in Lebanon [27] revealed that concurrent drug and alcohol use before and during sex was common at 41.9%, and another found that alcohol was available at 94.0% and multiple substances were available at 33.3%of group sex events in which MSM engaged [28]. Concurrent injection drug use appears to be low among Lebanese MSM, however, with less than 2.0% of MSM in one study reporting having ever injected drugs [28].

Commercial sexual activity was also common. In Morocco, while less than one fifth of participants reported paying for sex, 65.6% reported receiving money or gifts for sex [34]. Commercial sexual activity among Egyptian MSM was also common, at 33.2% in Cairo, 31.8% in Alexandria and a staggering 92.0% in Luxor [24, 36]. In Yemen, respondents reported having an average of 2.9 commercial partners to whom they sold sex and an average of 3 partners from whom they bought sex [38]. In Libya, anal intercourse with a commercial partner was practiced by 27.0% of the sample in the past six months and less than one in five of these respondents reported using a condom [33].

### Young sex workers

Eleven articles focused on sex workers particularly females who fell in our age range. Five articles out of the eleven took place in different regions across Iran. There was only one study on male sex workers, which was conducted in Lebanon. Studies indicated that sex workers are not yet well studied because they are hard to reach due to the illegal status of their profession and high levels of stigma in MENA [24].

#### Risky behavior

With respect to condom use, in general, unprotected sex was an alarming risk practice among sex workers. A study in Iran reported that 43.8% of sex workers never used condoms [40]. Similarly, in Syria, condom use in commercial sex was low, as reported by FSWs with 31.3% of participants using condoms every time, 14.2% most of the time and 16.0% sometimes. [39]. Egypt indicated similar outcomes with only 25.0% reporting condom use in last commercial sex [36].

Results were higher in Iran, Libya and Lebanon. FSWs in Iran using condoms in their last sexual contact with a client ranged between 39.3% and 57.1%, respectively [41, 42]. In Libya, 63.4% of FSW reported consistent condom use during sex with one-time clients, and 56.8% during sex with regular clients [33]. In Lebanon, 92.0% of the FSWs stated that they used a condom the last time they had sex with their regular male clients and 98.0% had done so with their nonregular male clients [19]. In another study in Lebanon, focusing on male sex workers, they differentiated between sex workers working in hammams (bathhouses) and escorts [43]. Sex workers from the hammams reported low condom use with clients (one of the nine sex workers consistently used condoms) while the majority of the escorts (five of seven) reported always using condoms with their clients as they are more educated and had higher HIV risk knowledge than hammam sex workers.

Generally, sex workers typically opted not to use condoms with non-client sex partners, in an effort to differentiate sex for work versus pleasure and was less than what is reported previously with paying clients [19, 43]. However, condom use was still significantly higher amongst sex workers with non-client sex partners in both Lebanon and Iran [19, 41], as opposed to Egypt and Syria [36, 39] but not as high with paying clients [19, 36, 39, 41].

#### HIV testing

HIV testing among this group varied significantly. In Libya, 38.6% of FSWs had been tested for HIV during the past year and knew the result [33]. Similar patterns were noted in Syria where only 35.3% indicated that they have ever tested for HIV/AIDS, among which only 22.8% were voluntarily tested [39]. Testing was much lower in Iran where only 8.0% of FSWs had been tested [44]. In Egypt, only 2% of the sample of the study were ever tested [36]. On the other hand, HIV testing in Lebanon was highly prevalent as 79.0% of the FSWs were tested [19]. Among those who had tested previously, 86.0% of the FSWs had taken the HIV test as part of a mandatory requirement since 80.0% of the sample are non-Lebanese and are expected to be tested in order to obtain residence or a work permit [19].

#### Overlapping risk

Regarding drug use, results again varied widely. In Sajadi et al. study [41] in Iran, 73.8% of FSWs reported a history of any drug use (Table 4). Of these, 63.2% were active drug users. Similar results in Iran were confirmed in the Ramzani Tehrani et al. study [44]. High numbers were also detected in Egypt where over half of FSWs reported ever abusing drugs and 6.0% reported injecting drugs in the past year [24]. Libya and Syria showed different patterns as only 3.0% of FSWs in Libya [33] ever injected drugs and 11.0% of FSWs in Syria indicated drug use [39] (Table 4).

**Table 4:** Risky behaviors among sex workers in the MENA region.

	Risky Behaviors Among Sex Workers
	**Iran**
**Injecting Drugs**	A total of 73.8% reported a history of any drug use. Of these, 63.2% were active drug users. (Sajadi et al. 2013) 60% used drugs and 2.5% used them intravenously (Tehrani et al. 2008) The vast majority (96.8%) had ever used drugs while injection was reported by 14.5% (Mirzazadeh et al. 2013)
**Married**	Ever being married: 83.2% (Sajadi et al. 2013) Currently married: 35.8% (Sajadi et al. 2013) Ever being married: 81.0% (Mirzazadeh et al. 2013) Currently married: 28.5% (Mirzazadeh et al. 2013)
**No. of Clients**	In last seven days: 3.1 (Sajadi et al. 2013) In last seven days: 3.4 (Mirzazadeh et al. 2013)
**Additional Income**	36.5% (Sajadi et al. 2013)
	**Egypt**
**Injecting Drugs**	50% used drugs and 6% injected drugs in the 12 months preceding the survey (FHI/MOH Egypt 2010)
**Married**	Ever being married: 89% (FHI/MOH Egypt 2010) Currently married: 45.5% (FHI/MOH Egypt 2010)
	**Syria**
**Injecting Drugs**	11% used drugs (Kobeissi 2014)
**Married**	Ever being married: 86% (Kobeissi 2014)
**Forced Sex**	35.8% (Kobeissi 2014)
**No. of Clients**	In last seven days: 4.8 (Kobeissi 2014)
	**Lebanon**
**Injecting Drugs**	0 (Mahfoud et al. 2010)
**Married**	Ever being married: 60% (Mahfoud et al. 2010) Currently married: 10% (Mahfoud et al. 2010)
**No. of Clients**	In last months: 96% had five or more clients (Mahfoud et al. 2010) Clients per year for hammer sex workers: 1,015 (median ¼ 1,095) (Aunon et al. 2015) Clients per year for escorts: 343 (median ¼ 313) (Aunon et al. 2015)
**Additional Income**	Most of male sex workers from the hammam relied on sex work as their only source of income whereas more than one half of the escorts also had another income generating activity (Aunon et al. 2015)
	**Libya**
**Injecting Drugs**	2.8% (Valadez et al. 2013)
**Forced Sex**	18.2% (Valadez et al. 2013)

### Students, general population and others

#### University students

Seven articles explored HIV-related risk factors among university students within the indicated age range, with four articles coming mainly from Iran [45–48], two from Lebanon [49, 50] and one from Egypt [51]. History of high-risk behaviors among university students included having multi-sex partners, having unsafe sex, drug use, alcohol consumption and being in the company of friends who engaged in heterosexual intercourse and were alcohol consumers, were fairly common [45–49, 51]. It was also more likely for male students to engage in risky behavior than their female counterparts [47, 49, 51]. In Iran and Lebanon, students who shared homes with each other are more likely to belong to social networks of people with high-risk behaviors and drug abuse compared to those who live with their families [48, 49]. Not discussing their sexual activity preferences with partners, in Lebanon, was associated with increased odds of not knowing about the effectiveness of condoms at preventing HIV [50]. A significant percentage (30.0%) of students in Egypt lacked adequate knowledge about HIV [51]. Also, low level of perceived susceptibility to be infected by HIV virus was common among university students which indicated that the students feel low risk or probability of being infected by HIV virus in the future [45, 46, 51].

#### General population and others

Regarding other bridging populations, twenty-two articles within the indicated age range included prisoners, street children, truck drivers, tourist workers, transgender women, conscripts and people of the general population. Participants in this group again had a limited understanding of HIV, especially around modes of transmission and the fairly high-risk behavior [44, 52–58].

For example, among tourism workers in Egypt, 69.2% knew the magnitude of the problem of HIV and 60.6% reported it was likely to get worse in the future compared to 58.0% and 53.4% respectively among industrial workers [59]. Rate of condom use is low among the general youth population, especially in Egypt, Iran, Iraq, Lebanon, and Palestine [24, 55, 60–65] and as seen in Table 5.

**Table 5:** Condom use among bridging populations in the MENA region.

	Condom Use Among Bridging Populations
	**Iran**
**Truck Drivers**	Last month: Out of 287 married truck drivers, 224 (78%) of them did not use condom (Maarefvand et al. 2015) Last month: Truck drivers who reported contacts with CSWs (123 (85.4%)) used condom (Maarefvand et al. 2015). Ever used condoms: 64.8% (Tehrani et al. 2008)
**Runaways (At Risk Women)**	During the last year: 22% (Hajiabdolbaghi et al. 2007)
**General Population**	During last sex: 35.1% (Housseini Hooshyar et al. 2018) 28.7% used condoms consistently (Honarvar et al. 2016) 21.8% used condoms consistently (26.1% male vs. 7.1% female) (Shokoohi et al. 2016 22.04% used condoms consistently (Mirzaee et al. 2017)
	**Egypt**
**Street** **Children**	During the last year: 20% used condoms consistently (Nada & El Daw 2010)
**General Population**	1% of female contraceptive users (15–49 years) use condoms (Abdel- Tawab et al. 2016)
	**Syria**
**Prisoners**	Ever used condoms: 22.8% (Kobeissi 2014) During last sex: 10.5% (Kobeissi 2014)
	**Iraq**
**General Population**	12% (Ismael et al. 2012)
	**Lebanon**
**Conscripts**	51% used condoms consistently (Adib et al. 2002)
**General Population**	Students: 36.8% (Kahhaleh et al. 2009) University graduates: 33.7% (Kahhaleh et al. 2009)
	**Palestine**
**General Population**	Some young men indicated that youths do not use condoms, especially when unplanned, or because some do not know how to use it. Others indicated that youths sometimes use condoms if they go to brothels, where the use of a condom is mandatory. (Massad et al. 2014)
	**Morocco**
**General Population**	According to participants, all Moroccan men and boys always have a condom in their pocket. (Hendrickx et al. 2002)

Among street children in Egypt who have sex, most never used a condom at all and most had multiple sex partners [66]. Condom use is more common among men, especially those who practice extramarital sex in Egypt, Iran, and Morocco [23, 24, 60, 62, 64, 67] and more frequently used among older children as opposed to those who are younger in age [64, 68]. It is worth noting that weakened religion among youth in Libya [53], Morocco [69], and Southern Iran [60] was reported to be one of the factors causing street children to engage in risky behaviors.

Testing was limited among the general population as indicated by multiple studies with the highest rate being in Iran at 23.2% [44, 60, 62]. Among transgender women in Lebanon, only 43.0% of participants had been tested for HIV within the last 12 months, whereas 40.0% had never been tested [70]. In both Lebanon and Iran, studies reveal that condom use is very limited in this group, reaching 43.0% and 13.3%, respectively [70, 71].

## Discussion

To our knowledge, this scoping review is the first to examine epidemiological HIV risk factors and underlying risk context for youth residing in or originating from MENA. Even though we are more than three decades into the HIV epidemic, our findings indicate that key populations engage in multiple risk behaviors that could potentially spread further the epidemic in the region despite its low prevalence [8]. Among PWIDs, all evidence consistently indicates that HIV has already established itself among a number of PWID populations in MENA [8]. Therefore, further research as well as HIV testing are needed to understand the magnitude and potential spread of HIV across this key population. The levels of risky behavior practices, such as the use of nonsterile injecting equipment, inconsistent condom use and selling/buying sex have been documented as significant, confirming the potential for further HIV spread among PWIDs. Availability, access and increasing awareness towards harm reduction services are crucial in addressing the high-risk context affecting this key population.

Regarding MSM, they are considered one of the hardest groups to reach given the severe stigmatization they are usually subjected to [8]. However, Lebanon seems to be standing out especially considering the availability of information on this particular group in the country and the relatively high HIV testing trends among them. Across all MENA countries that studied the matter and are included in this scoping review, it is evident that low condom use is very common within this key population and thus, special attention needs to be given with regards to increasing awareness and access to condom use. Also, definitive data are still too limited to document trends on MSM, especially in Iran. MSM remains to be a key population that requires special attention in the MENA region.

The illegal status of sex work in most countries and their young age at first sexual intercourse for profit (Table 3) may have limited the accessibility to HIV awareness and messages which would, thus, allow more spread of the epidemic. Comparing them to other key populations, condom use among sex workers could be considered higher, especially in Lebanon. However, migrants and refugees who engage in such practices may require more exposure to information and services on harm reduction as they are particularly a vulnerable group who may not own the sufficient knowledge on HIV prevention and protection. Also, delaying the average age of first sex for profit generally may contribute to the increased awareness and limited involvement in risky behaviors [46].

Multi-sex partners, having unsafe sex, drug use, alcohol consumption and belonging to a social network practicing risky behaviors are valid reasons for putting university students at a high risk for contracting HIV. Despite their high education, some lacked adequate knowledge on the matter which thus, does not inform their attitudes and leads to a low risk perception. It is also worth noting that young people are disproportionately affected by political instability and war which is prevalent in the MENA region and may have enhanced their vulnerability to drug use [53].

With the youth and bridging populations, peer pressure and young people’s inhibition about discussing sexual and reproductive health matters with credible sources are also a few of the factors underlying low utilization of condoms. Gender and age factors as well as adoption of more liberal lifestyles in some settings are potential contributors to this pattern as well [72]. The evident reluctance to access services especially HIV testing needs to be addressed. Moreover, special attention needs to be given to trans-gendered women who engage in high-risk behavior, especially due to the current dearth of the literature involving this population which may hamper the implementation of policies and programs. However, increased awareness alone is insufficient as mentioned earlier. A qualitative study involving five focus group discussions and 15 in-depth interviews conducted in Aden, Yemen examined the role of peer educators in addressing risk behaviours in MENA [73]. The study revealed key enabling factors that facilitated the implementation of a community peer education program for youth HIV prevention in four poor and vulnerable areas of Aden, Yemen, resulting in improved HIV knowledge and risk perception and decreased stigma and risky behavior. An approach that involves community participation, mobilization of targeted communities, and capacity building of all those included may be necessary to change risk context in the MENA [73].

### Limitations

We recognize several limitations in our scoping review. First, limitations in the literature due to heterogeneity in risk behaviors and risk contexts that characterize the MENA region makes it difficult to generalize the findings of these studies. Second, our process of selecting articles and literature that meet the inclusion criterion related to age is not perfect. The lack of homogeneity in studies conducted in MENA, often due to limited funding resources, makes it especially hard to locate studies entirely focused on youth.

Reporting of results in these studies is often not homogenous which makes it harder to get separate breakdown by age in some of the studies. Moreover, the scarcity of bio-behavioral surveys conducted in the region makes it more challenging to have a clearer idea about long term trends. Nevertheless, every effort was made on the part of our team to offset some of these challenges. We extracted information on the full range of participant age, in addition to median/mean in order to see who is most featured in the literature: middle vs later adolescents vs older youth. Only studies with the bulk of the sample clustered between the age range of 16–29 were included. Finally, given that HIV is highly stigmatized in the MENA region and the disclosure of sexual attitudes is considered undesirable, reporting accurate behavioral data may be hindered. Therefore, findings should be interpreted with social desirability bias possibility in mind.

## Conclusion

This scoping review shows that there is a predominant lack of research on epidemiological HIV risk factors and underlying risk context for youth residing in or originating from MENA. Despite the heterogeneity in risk behaviors and risk contexts that characterize the MENA region, the information gathered allows for identifying gaps in the research which will inform policies and programs for improving current practices related to HIV transmission among youth in MENA.

## Supporting information

S1 Checklist PRISMA-ScR. (DOCX)

S1 Table Search strategy. (DOCX)

S1 Appendix Article review and data abstraction. (PDF)
