# Supplementary material for: Understanding the epidemiological HIV risk factors and underlying risk context for youth residing in or originating from the Middle East and North Africa (MENA) region: A scoping review of the literature
Source: PLoS One. 2022 Jan 7;17(1):e0260935. doi: 10.1371/journal.pone.0260935 (PMC8741013; doi:10.1371/journal.pone.0260935)
Supplement: S1 Table — (DOCX) [file pone.0260935.s002.docx]

Additional file 1

Search strategy. Ovid MEDLINE: Epub Ahead of Print, In-Process & Other Non-Indexed Citations, Ovid MEDLINE® Daily and Ovid MEDLINE® <1946-Present>


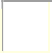


| **#** | **Search Statement** | **Results** |
| --- | --- | --- |
| 1 | HIV.mp. | 345147 |
| 2 | (Sexually transmitted disease* or STD*).mp. | 41865 |
| 3 | Sexually transmitted infection*.mp. | 13067 |
| 4 | STIs.mp. | 5255 |
| 5 | syphilis.mp. | 34109 |
| 6 | gonorrhea.mp. | 15921 |
| 7 | gonorrhoea.mp. | 4060 |
| 8 | chlamydia.mp. | 29067 |
| 9 | (sexual adj3 health).mp. | 13558 |
| 10 | sexuality.mp. | 18727 |
| 11 | (Algeria* or Bahrain* or Egypt* or Iran* or Iraq* or Israel* or Jordan* or Kuwait* or Lebanon* or Lebanese or Libya* or Morocc* or Oman* or Palestin* or Qatar* or Saudi or Syria* or Tunisia* or UAE or Emirat* or Yemen*).mp. | 206254 |
| 12 | (MENA or Middle East* or North Africa*).mp. | 21075 |
| 13 | Arab*.mp. | 141916 |
| 14 | 1 or 2 or 3 or 4 or 5 or 6 or 7 or 8 or 9 or 10 | 456605 |
| 15 | 11 or 12 or 13 | 328377 |
| 16 | 14 and 15 | 4246 |
| 17 | youth*.mp. | 75292 |
| 18 | young adult.mp. or exp Young Adult/ | 789162 |
| 19 | student*.mp. | 296994 |
| 20 | adolescent*.mp. | 2014903 |
| 21 | young*.mp. | 1328306 |
| 22 | 17 or 18 or 19 or 20 or 21 | 3021309 |
| 23 | 16 and 22 | 1311 |
