# Supplementary material for: Understanding the epidemiological HIV risk factors and underlying risk context for youth residing in or originating from the Middle East and North Africa (MENA) region: A scoping review of the literature
Source: PLoS One. 2022 Jan 7;17(1):e0260935. doi: 10.1371/journal.pone.0260935 (PMC8741013; doi:10.1371/journal.pone.0260935)
Supplement: S1 Appendix — (PDF) [file pone.0260935.s003.pdf]

Refid	Author	Title	Abstract	User	Level	Author	Date	Jurisd	PubType	PubType_comment	Quant-CaseContr
2	G. Wagner, B. Ghosh-Dastidar, C. El Khoury, C. Abi Ghanem, M. G. Mutchler, E. Balan, H. Green, S. Kegeles, J. Mokhbat	A Serial, Cross-Sectional Comparison of Condomless Anal Sex and HIV Testing Among Young MSM in Beirut, Lebanon	In 2012, our research with young men who have sex with men (YMSM) in Beirut showed high rates of recent condomless anal sex and low rates of recent HIV testing. In 2017, we collected data from YMSM to assess for temporal changes and sociodemographic correlates. Propensity score weighting was used to eliminate any sociodemographic differences between the 2012 (n = 164) and 2017 (n = 226) samples of YMSM (age 18-29) recruited with long-chain peer referral sampling. Regression analysis was used to examine sociodemographic correlates of recent condomless anal sex and	mohammad.alakel	5	Wagner	2019	Beirut, Lebanon	PubPeer		

Refid	Quant-CrossSect	Quant-Other	Quant-Other_comment	Qual-FocGroup	Qual-SemiStr	Mixed	Aim	Dates	InclExclCriteria	RecruitmentMethods	DataCollection
2	Quant-CrossSect						To examine sociodemographic correlates of recent condomless anal sex and HIV testing among young MSM in Beirut in 2012 and 2017 among young men who have sex with men in Beirut in 2012 and 2017.	2012 study dates not given<br><br>07/2016-03/2017	Being a cisgender male, age 18–29 years, fluent in English or Arabic, residing in greater Beirut, and having had oral or anal sex with a man in the past 12 months. The same criteria were used in the 2012 study, with the exception of age as it enrolled men age 18 and above; therefore, for this analysis, we used only data from participants aged 18–29 years in the 2012 study	Recruited using long-chain peer referral sampling. Recruitment began with a small number of eligible persons who were identified through recommendations from community organizations working with MSM and study consultants. All participants, including seeds, received recruitment coupons to recruit members of their social network, resulting in multiple waves of participants.	The same survey measures were used in both studies and were administered in English or Arabic, depending on the preference of the participant, with computer-assisted interview software, and on average took about 1 h to administer. The survey was developed in English and translated into Arabic using standard translation and back translation methods. Participants were given the option of completing the survey on their own or having the interviewer administer the survey

Refid	Participants	Participants_Youth	Age_mean	Gender_Percentage	Pop_Largest	SampleOther	Outcome	Comparator	RiskFactors	Intervention	MainFindings	Notes
2	The sample recruited in 2017 consisted of 226 MSM. 2012 study (N = 164)	N=390	age range=18-29: 2012 sample mean (sd) age=23.4 (3.0); 2017 sample mean (sd) age weighted=23.5 (3.4	100% male	100% MSM	Low income: 2012 sample=78% 2017 sample=78.8%	Outcomes: 1) any condomless anal sex with men in the past 3 months regardless of partner HIV status, (2) any condomless anal sex in the past 3 months with men of unknown or positive HIV status, (3) condomless sex during last anal sex encounter with a man, and (4) any HIV testing within the past 6 months	Comparisons of the sexual risk behavior and HIV/STI testing characteristics of the 2012 and 2017 study samples	Factors positively associated with having condomless anal sex regardless of partner's HIV status: Having current employment, having any university education, being in a committed relationship, and having low income; each of which increased the odds of having condomless anal sex by two- to nearly fourfold.	N/A	Data collected from MSM in Beirut in 2012, and 5 years later in 2017, suggest that while high levels of condomless anal sex continue to highlight the need for HIV prevention efforts to remain a public health priority, such high-risk sexual behaviors may be decreasing and HIV testing increasing. In the 2017 sample, any recent condomless anal sex with partners whose HIV status was positive or unknown was associated with employment and Christian religious afliation. Low income was the sole correlate of having recently tested for HIV. These findings suggest a temporal trend toward increased HIV	

Refid	Author	Title	Abstract	User	Level	Author	Date	Jurisd	PubType	PubType_comment	Quant-CaseContr	Quant-CrossSect	Quant-Other	Quant-Other_comment	Qual-FocGroup	Qual-SemiStr	Mixed	Aim	Dates	InclExclCriteria	RecruitmentMethods	DataCollection	Participants	Participants_Youth	Age_mean	Gender_Percentage	Pop_Largest	SampleOther	Outcome	Comparator	RiskFactors	Intervention	MainFindings	Notes
			HIV testing using the 2017 sample, and whether these behaviors differed between the samples. Compared to the 2012 sample, the 2017 sample was about half as likely to report any condomless anal sex in the past 3 months with partners whose HIV status was positive or unknown (14% vs. 23%; OR [95%CI] 0.56 [0.32, 0.98]), and nearly 2.5 times as likely to report HIV testing in the past 6 months (48% vs. 27%; OR [95%CI] 2.44 [1.46, 4.10]). In the 2017 sample, any recent condomless anal sex with partners whose HIV status was positive or unknown was associated with																														protective behaviors among YMSM in Beirut over the past 5 years	

Refid	Author	Title	Abstract	User	Level	Author	Date	Jurisd	PubType	PubType_comment	Quant-CaseContr	Quant-CrossSect	Quant-Other	Quant-Other_comment	Qual-FocGroup	Qual-SemiStr	Mixed	Aim	Dates	InclExclCriteria	RecruitmentMethods	DataCollection	Participants	Participants_Youth	Age_mean	Gender_Percentage	Pop_Largest	SampleOther	Outcome	Comparator	RiskFactors	Intervention	MainFindings	Notes
			employment and Christian religious affiliation. Low income was the sole correlate of having recently tested for HIV. These findings suggest a temporal trend toward increased HIV protective behaviors among YMSM in Beirut over the past 5 years. Further inroads could be secured if HIV prevention programming targeted economic influences.																															
58	S. Hosseini Hooshyar, M. Karamouzian, A. Mirzazadeh, A. A. Haghdoost, H. Sharifi, M. Shokoohi	Condom Use and its Associated Factors Among Iranian Youth: Results From a Population-Based Study	BACKGROUND : Given the young structure of Iran's population and the fact that extramarital sexual relationships are both prohibited by legislation and shunned by society and religion, examining condom use practices among	mohammad.alakel	5	Hooshyar et al.,	2018	Iran	PubPeer			Quant-CrossSect						To explore condom use and its correlates among Iranian young adults.	01/2013 to 03/2013	Not reported	A total of 4950 participants were recruited through a non-random multistage sampling approach	-Data were collected through a self-administered questionnaire where the respondents completed the survey on their own and passed it to trained gender-matched interviewers.<br>-13 provinces, including Iran's capital Tehran, and 12 other	N= 4950	N= 632	The mean age (SD) of the included participants was 24.4 (3.1); it was 24.5 (3.1) and 24.2 (3.0) for men and women respectively (P value = .376).<br>-Range: 19-29	More than three quarters (n = 488; 77.1%) were men and the rest were women.	The youth population having extramarital sex	-70% of the participants were selected from the capital city and 30% from non-capital cities<br>-Most participants had a job in the government sector or ran their own job (n = 303; 48.0%), had more than a diploma (n = 230; 39.5%	To assess condom use at last sex, participants were asked a single question: "Did you use a condom the last time you had penetrative sex?" This variable was coded as "yes" if they reported using a condom at last sex; otherwise it was	-Comparing condom use by gender: Men reported significantly higher condom use at last sex than women (38.5% vs. 25.7%).	-.A considerable proportion of the participants started having sex <19 years old (n = 170; 41.3%). Mean (SD) age at first sex for men and women was 19.0 (3.3) and 20.2 (3.2), respectively (P = .001)*<br>-Of the whole sample, 40.0% reported having	N/A	-Only one third of sexually active young adults reported condom use at last sex.<br>-Young women were found to be less likely to use a condom at last sex.<br>-Young people who knew more about condoms were more likely to have used it at last sex.	

Refid	Author	Title	Abstract	User	Level	Author	Date	Jurisd	PubType	PubType_comment	Quant-CaseContr	Quant-CrossSect	Quant-Other	Quant-Other_comment	Qual-FocGroup	Qual-SemiStr	Mixed	Aim	Dates	InclExclCriteria	RecruitmentMethods	DataCollection	Participants	Participants_Youth	Age_mean	Gender_Percentage	Pop_Largest	SampleOther	Outcome	Comparator	RiskFactors	Intervention	MainFindings	Notes
			Iranian youth is highly important. The aim of this study was to explore condom use and its correlates among Iranian young adults. METHODS: In a sample of 3,045 individuals aged 19-29 who were recruited from a nation-wide study, we analyzed data from 633 participants who reported a history of extramarital sex. Subjects were asked about their condom use practices during their last penetrative sex. Data were collected through a self-administered questionnaire where the respondents completed the survey on their own and passed it to trained gender-matched interviewers. Multivariable regression models were																			provinces were selected for data collection						AS/BS degree and n = 277; 43.9% more than BS degree), and were currently unmarried (n = 447; 71.6%).	coded as “no.” Reasons for not using a condom were also asked from those who reported not using a condom. -We included the following two main variables in our analysis. I) the age at first sex was inquired about and coded as a binary variable (?18 vs. >18 years old); II) whether or not the participant had ever had sex while high on drugs or alcohol.		sex while high on drugs or alcohol.		-Sex under the influence of alcohol or drugs was frequent and associated with reduced condom use among young adults.	

Refid	Author	Title	Abstract	User	Level	Author	Date	Jurisd	PubType	PubType_comment	Quant-CaseContr	Quant-CrossSect	Quant-Other	Quant-Other_comment	Qual-FocGroup	Qual-SemiStr	Mixed	Aim	Dates	InclExclCriteria	RecruitmentMethods	DataCollection	Participants	Participants_Youth	Age_mean	Gender_Percentage	Pop_Largest	SampleOther	Outcome	Comparator	RiskFactors	Intervention	MainFindings	Notes
			constructed to report adjusted odds ratios (AOR) along with 95% CI. RESULTS: Of the 633 participants, 222 (35.1%) reported condom use at last sex. Men reported significantly higher condom use than women (38.5% vs. 25.7%). Having a stable job (AOR = 1.86, 95% CI: 1.01, 3.43), higher knowledge of condom use (AOR = 1.57, 95% CI: 1.03, 2.37) and sexual transmission of HIV (AOR = 1.83, 95% CI: 1.18, 2.85) were positively associated with condom use at last sex. Conversely, experience of sex under the influence of substances (AOR = 0.66, 95% CI: 0.45, 0.94) was																																

Refid	Author	Title	Abstract	User	Level	Author	Date	Jurisd	PubType	PubType_comment	Quant-CaseContr	Quant-CrossSect	Quant-Other	Quant-Other_comment	Qual-FocGroup	Qual-SemiStr	Mixed	Aim	Dates	InclExclCriteria	RecruitmentMethods	DataCollection	Participants	Participants_Youth	Age_mean	Gender_Percentage	Pop_Largest	SampleOther	Outcome	Comparator	RiskFactors	Intervention	MainFindings	Notes
			significantly associated with reduced odds of condom use at last sex. CONCLUSION: This study shows that only one out of every three young adults reported using condoms at last sex. While educational programs are helpful, multi-sectoral approaches (eg, individual-, community-, and structural-level interventions) are required to change sexual behaviours towards safe sex practices and reinforce negotiating condom use among youth.																															
67	F. M. Elamouri, P. M. Musumari, T. Techasrivichien, A. Farjallah, S. Elfandi, O. F. Alsharif, H. Benothman, S. P. Suguimoto, M. Ono-Kihara,	"Now drugs in Libya are much cheaper than food": A qualitative study on substance use among young Libyans in post-revolution	BACKGROUND: Libya is facing a rapidly growing epidemic of illicit drug use and HIV. This situation is fueled by a complex array	mohammad.alakel	5	Elamouri	2018	Tripoli, Libya	PubPeer							Qual-SemiStr		To examine if young people in Libya are vulnerable groups to both HIV and illicit drug use	09/2015-10/2015	Two groups of young Libyans aged 14–18 years from community-based informal re-education and rehabilitation centers or from high schools in	We used maximum variation, purposive sampling strategy to recruit participants.	- Transcripts of audio and hand-written records were translated into English for data analysis.<br>- Three investigators independently coded the data	31 participants were recruited	N=31	The median age of participants was 16 years.<br>Minimum age is: 14<br>Maximum age is: 18	The total sample consisted of 6 females and 25 males (81% male).	52% prisoners/residents of rehab centers.	All 16 prisoners reported ever using drugs.	Interviews covered topics related to drug use and HIV-related risky behaviors; particularly, (1) knowledge of the risk of injection and	N/A	Risk factors for drug use included peer influence, the increased availability and affordability of drugs, disruption of social life and	N/A	The increased availability and affordability of drugs, provided the frame within which other factors, such as peer influence, insufficient knowledge of	Focuses on knowledge/perception related to drug use and HIV, but also touches on experience with injection and noninjection drug use which

Refid	Author	Title	Abstract	User	Level	Author	Date	Jurisd	PubType	PubType_comment	Quant-CaseContr	Quant-CrossSect	Quant-Other	Quant-Other_comment	Qual-FocGroup	Qual-SemiStr	Mixed	Aim	Dates	InclExclCriteria	RecruitmentMethods	DataCollection	Participants	Participants_Youth	Age_mean	Gender_Percentage	Pop_Largest	SampleOther	Outcome	Comparator	RiskFactors	Intervention	MainFindings	Notes
	M. Kihara	Tripoli, Libya	of factors, mainly the consequences of the political and military turmoil of the Arab Spring. Although it is extensively documented in other settings that young people are one of the most vulnerable groups to both HIV and illicit drug use, no study has explored this issue among young people in Libya. The current study addresses this research gap. METHODS: This study is a qualitative study using in-depth interviews guided by a semi-structured questionnaire. We used a maximum variation, purposive sampling strategy to recruit male and female participants, aged 14-18																	Tripoli.		transcripts and generated the initial codes.							non-injection drug use, (2) experience with injection and noninjection drug use, (3) perception of factors that lead to substance use, (4) knowledge related to HIV prevention, and (5) perception of factors that prevent the use of injection and non-injection drugs among young Libyans.		healthy recreational activities, and the distress and casualties of the war.<br>Risk factors for HIV were insufficient knowledge related to HIV transmission and unsafe injection practices, such as sharing needles and syringes.<br>Protective factors were religious beliefs and practices, good parent-child connectedness, and high self-esteem and future aspiration.		substance use, and HIV, operated to increase the vulnerability of young people to drugs and HIV, while religious beliefs and parent-child connectedness acted as protective factors.	may be relevant.

Refid	Author	Title	Abstract	User	Level	Author	Date	Jurisd	PubType	PubType_comment	Quant-CaseContr	Quant-CrossSect	Quant-Other	Quant-Other_comment	Qual-FocGroup	Qual-SemiStr	Mixed	Aim	Dates	InclExclCriteria	RecruitmentMethods	DataCollection	Participants	Participants_Youth	Age_mean	Gender_Percentage	Pop_Largest	SampleOther	Outcome	Comparator	RiskFactors	Intervention	MainFindings	Notes
			years, from schools, prisons, and community-based informal re-education and rehabilitation centers in Tripoli, Libya. RESULTS: In total, 31 participants were recruited: 6 females and 25 males. Sixteen participants were prisoners and residents of community-based informal re-education and rehabilitation centers, and 15 were recruited in schools. Risk factors for drug use included peer influence, the increased availability and affordability of drugs, disruption of social life and healthy recreational activities, and the distress and casualties of the war. Protective factors were religious beliefs																															

Refid	Author	Title	Abstract	User	Level	Author	Date	Jurisd	PubType	PubType_comment	Quant-CaseContr	Quant-CrossSect	Quant-Other	Quant-Other_comment	Qual-FocGroup	Qual-SemiStr	Mixed	Aim	Dates	InclExclCriteria	RecruitmentMethods	DataCollection	Participants	Participants_Youth	Age_mean	Gender_Percentage	Pop_Largest	SampleOther	Outcome	Comparator	RiskFactors	Intervention	MainFindings	Notes
			and practices, good parent-child connectedness, and high self-esteem and future aspiration. Risk factors for HIV were insufficient knowledge related to HIV transmission and unsafe injection practices, such as sharing needles and syringes. CONCLUSION: We found individual, interpersonal, family, and structural-level factors that interplayed to shape the vulnerability of young people to drug use and HIV infection in Tripoli, Libya. Structural factors, including the increased availability and affordability of drugs, provided the frame within which other factors, such as peer influence,																															

Refid	Author	Title	Abstract	User	Level	Author	Date	Jurisd	PubType	PubType_comment	Quant-CaseContr	Quant-CrossSect	Quant-Other	Quant-Other_comment	Qual-FocGroup	Qual-SemiStr	Mixed	Aim	Dates	InclExclCriteria	RecruitmentMethods	DataCollection	Participants	Participants_Youth	Age_mean	Gender_Percentage	Pop_Largest	SampleOther	Outcome	Comparator	RiskFactors	Intervention	MainFindings	Notes
			insufficient knowledge of substance use, and HIV, operated to increase the vulnerability of young people to drugs and HIV, while religious beliefs and parent-child connectedness acted as protective factors. Multisectoral efforts and studies to quantitatively evaluate the magnitude and distribution of these problems are urgently needed.																															
174	H. Sharifi, A. Mirzazadeh, A. Noroozi, B. D. Marshall, A. Farhoudian, P. Higgs, M. Vameghi, F. Mohhamadi Shahboulaghi, M. Qorbani, O. Massah, B. Armoon, M. Noroozi	Patterns of HIV Risks and Related Factors among People Who Inject Drugs in Kermanshah, Iran: A Latent Class Analysis	The objective of this study was to explore patterns of drug use and sexual risk behaviors among people who inject drugs (PWID) in Iran. We surveyed 500 PWID in Kermanshah concerning demographic characteristics, sexual risk behaviors, and	mohammad.alakel	5	Sharifi	2017	Kermanshah, Iran	PubPeer			Quant-CrossSect						To explore patterns of drug use and sexual risk behaviors among people who inject drugs in Iran.	09/ 2014 till 12/2014	Aged over 18 years, had reported injecting drugs in the past month, had been residing in Kermanshah for a minimum of one month, were able to speak and comprehend Farsi enough to respond to the survey questions, and	Using snowball sampling techniques, we recruited 500 PWID in Kermanshah.	A cross-sectional survey. The questionnaire included modules on socio-demographic characteristics, sexual behaviors, HIV testing, participation in harm reduction programs, frequency of injection, injection-related	500	220	Participants ranged in age from 18 to 68 years (median = 28 years; SD = 7.6.85 years).	Not reported.	100% PWID	90% of participants were low income	The questionnaire included modules on socio-demographic characteristics, sexual behaviors, HIV testing, participation in harm reduction programs, frequency of injection, injection-related risk behaviors such as sharing	Compared lowest risk class to highest risk class members; moderate risk class compared to high risk class	Positively associated with risk behaviours: Low income, education, and homelessness produce unsafe sexual and injecting behaviors as result of a need for drugs, housing, and economic resources. Negatively associated: use	N/A	Homeless people who inject drugs were more likely to have multiple HIV risks and were most likely to belong to the high-risk subgroup. People who inject drugs and who used methamphetamine were more likely to belong to members of the	

Refid	Author	Title	Abstract	User	Level	Author	Date	Jurisd	PubType	PubType_comment	Quant-CaseContr	Quant-CrossSect	Quant-Other	Quant-Other_comment	Qual-FocGroup	Qual-SemiStr	Mixed	Aim	Dates	InclExclCriteria	RecruitmentMethods	DataCollection	Participants	Participants_Youth	Age_mean	Gender_Percentage	Pop_Largest	SampleOther	Outcome	Comparator	RiskFactors	Intervention	MainFindings	Notes
			drug-related risk behaviors in the month prior to study. We used latent class analysis (LCA) to establish a baseline model of risk profiles and to identify the optimal number of latent classes, and we used ordinal regression to identify factors associated with class membership. Three classes of multiple HIV risk were identified. The probability of membership in the high-risk class was 0.33, compared to 0.26 and 0.40 for the low- and moderate-risk classes, respectively. Compared to members in the lowest-risk class (reference group), the highest-risk class members had higher odds of being homeless (OR = 4.5, CI: 1.44-8.22; p =																	were able to provide informed consent.		risk behaviors							of syringes/needles, and number of sex partners they had over the 12 months prior to the interview. Receptive syringe sharing (RSS) was defined as injecting with a syringe after someone else has already used it. The unprotected sex variable was derived from survey questions which asked participants about engaging in sex without using a condom in the last 12 months (with both commercial sex workers and casual partners).		of needle and syringe programs.		highest-risk class than members of the lowest-risk class. Interventions should focus on people who inject drugs who are homeless, with low income and low education.	

Refid	Author	Title	Abstract	User	Level	Author	Date	Jurisd	PubType	PubType_comment	Quant-CaseContr	Quant-CrossSect	Quant-Other	Quant-Other_comment	Qual-FocGroup	Qual-SemiStr	Mixed	Aim	Dates	InclExclCriteria	RecruitmentMethods	DataCollection	Participants	Participants_Youth	Age_mean	Gender_Percentage	Pop_Largest	SampleOther	Outcome	Comparator	RiskFactors	Intervention	MainFindings	Notes
			0.001) in the past 12 months. Members of the high-risk class had lower odds of regularly visiting a needle and syringe exchange program as compared to the lowest-risk class members (AOR = 0.42, CI: 0.2-0.81; p = 0.01). Findings show the sexual and drug-related HIV risk clusters among PWID in Iran, and emphasize the importance of developing targeted prevention and harm reduction programs for all domains of risk behaviors, both sexual and drug use related.																															
182	M. Shokoohi, M. Karamouzian, A. Mirzazadeh, A. Haghdoost, A. A. Rafierad, A. Sedaghat, H. Sharifi	HIV Knowledge, Attitudes, and Practices of Young People in Iran: Findings of a National Population-Based Survey in 2013	INTRODUCTION: The evidence is mixed on the HIV knowledge, attitude, and practices of youth in Iran. The aim of the current study was to assess knowledge,	mohammad.alakel	5	Shokoohi	2016	13 provinces in Iran	PubPeer			Quant-CrossSect						To assess knowledge, attitudes, and practices of Iranian youth towards HIV through a national survey	01/2013 to 03/2013	young men and women aged 15–29 from 13 out of 31 provinces in Iran	multistage cluster sampling	Data were collected using a questionnaire	N=4950	N=4950	-Range: 15–29<br>-The mean (standard deviation (SD)) age was 21.8 (5.6) years	2456 men and 2412 women Hence, 50.45% Male	100% Young Persians	-Most participants were single (70.7%), had some levels of university educations (58.4%), and around 7.3% reported to be unemployed	-Had ever tested for HIV<br>-Had ever had extramarital sex<br>-Had ever injected drugs	Comparing knowledge, attitude and risk factors among males and females	-While younger participants reported lower condom use practices, there was no significant difference between the two age groups (P-value = 0.177)	N/A	The findings showed that Iranian youth and young adults have relatively insufficient overall knowledge and negative attitudes about	

Refid	Author	Title	Abstract	User	Level	Author	Date	Jurisd	PubType	PubType_comment	Quant-CaseContr	Quant-CrossSect	Quant-Other	Quant-Other_comment	Qual-FocGroup	Qual-SemiStr	Mixed	Aim	Dates	InclExclCriteria	RecruitmentMethods	DataCollection	Participants	Participants_Youth	Age_mean	Gender_Percentage	Pop_Largest	SampleOther	Outcome	Comparator	RiskFactors	Intervention	MainFindings	Notes
			attitudes, and practices of Iranian youth towards HIV through a national survey. MATERIALS AND METHODS: Through a cross-sectional study with multistage cluster sampling, we administered a pilot-tested standard questionnaire to assess the levels of HIV knowledge, attitudes and practices of individuals aged 15-29 years old. Participants were recruited from 13 provinces in Iran and consisted of 2456 men and 2412 women. RESULTS: Only 37.3% of the participants had a high knowledge score. Most participants knew the main routes of HIV transmission; however, misconceptions																												-About 40% had ever used alcohol or drugs during or before sex (39.8% male vs. 40.3% female)<br>-Only 13.1% had ever tested for HIV<br>-20.8% had ever had extramarital sex (31.7% male vs. 9.6% female) and and 27.6% had never or rarely used condoms<br>-1.8% ever injected drugs (2.9% male vs. and 0.7% female)<br>-Among sexually active subjects in this age group, only 21.8% (26.1% male vs. 7.1% female) were consistent condom users<br>-Among those aged 19–29 years old, the main source of HIV information was mass media (69.1%)<br>-Main reasons for non-condom use were inaccessibility (38.4%),		HIV and PLHIV. oreover, despite youth’s knowledge about reduced risk of HIV transmission via safe sex practices, less than one-third of them had used a condom in their last sexual encounter, and only 21.8% were consistent condom users. Moreover, around 30% of the participants perceived consistent condom use as unnecessary.	

Refid	Author	Title	Abstract	User	Level	Author	Date	Jurisd	PubType	PubType_comment	Quant-CaseContr	Quant-CrossSect	Quant-Other	Quant-Other_comment	Qual-FocGroup	Qual-SemiStr	Mixed	Aim	Dates	InclExclCriteria	RecruitmentMethods	DataCollection	Participants	Participants_Youth	Age_mean	Gender_Percentage	Pop_Largest	SampleOther	Outcome	Comparator	RiskFactors	Intervention	MainFindings	Notes
			existed about the transmission of HIV through mosquito bites across all age groups (31.7% correct response). Positive levels of attitude wereobserved among 20.7% of the participants. Most participants believed that people living with HIV (PLHIV) should be supported (88.3%) while only 46.3% were ready to share a table with them. Among those aged 19-29 years old, the main source of HIV information was mass media (69.1%), only 13.1% had ever tested for HIV, around 20.8% had ever had extramarital sex (31.7% male vs. 9.6% female),1.8% ever injected drugs (2.9% male vs. and 0.7% female).																												unnecessity (32.3%), and disinterest (28.8%)			

Refid	Author	Title	Abstract	User	Level	Author	Date	Jurisd	PubType	PubType_comment	Quant-CaseContr	Quant-CrossSect	Quant-Other	Quant-Other_comment	Qual-FocGroup	Qual-SemiStr	Mixed	Aim	Dates	InclExclCriteria	RecruitmentMethods	DataCollection	Participants	Participants_Youth	Age_mean	Gender_Percentage	Pop_Largest	SampleOther	Outcome	Comparator	RiskFactors	Intervention	MainFindings	Notes
			Among sexually active subjects in this age group, only 21.8% (26.1% male vs. 7.1% female) were consistent condom users. CONCLUSIONS: The findings showed that Iranian youth and young adults have relatively insufficient overall knowledge and negative attitudes about HIV and PLHIV. Novel strategies involving schools and youth's networks could be employed to deliver a culturally sensitive sexual health program.																															
190	R. L. Kaplan, J. McGowan, G. J. Wagner	HIV prevalence and demographic determinants of condomless receptive anal intercourse among trans feminine individuals in Beirut, Lebanon	INTRODUCTION: Growing evidence suggests increased HIV incidence in the Middle East and North Africa among "key populations." To date,	mohammad.alakel	5	Kaplan	2016	Beirut, Lebanon	PubPeer			Quant-CrossSect						To assess demographic correlates of risk behaviour and the prevalence of HIV among trans feminine individuals in Lebanon	05/ 2012 to 12/2012	Eligibility criteria of having been assigned male sex at birth and identifying as a woman or trans feminine, being 18 years or older, being fluent in Arabic or English, and	Long-chain referral sampling (snowball sampling) was used.	Data were collected using interviewer-administered questionnaires.	N= 53	Approx. 75% aged 30 or younger. (approx. n=40)	The median age = 22 years, with a range of 18 to 58 years.	100% Transwomen	100% Transwomen	-Most participants (89%) had been born in Lebanon and just over half (54%) of the sample identified as Muslim. -40% of participants	-Age of sexual debut, incidence of insertive anal sex with a man within the last three months, incidence of sex with a woman within the last three months, current	Demographic characteristics compared by condomless receptive anal intercourse (CRAI) in the last three months	-The majority of participants (66%) reported engaging in sex for income -40% of participants had never been tested for HIV -An overwhelming	N/A	-There is a high prevalence of HIV risk behaviour, particularly sex work and condomless receptive anal intercourse (CRAI), among the trans	Related to ref id 193

Refid	Author	Title	Abstract	User	Level	Author	Date	Jurisd	PubType	PubType_comment	Quant-CaseContr	Quant-CrossSect	Quant-Other	Quant-Other_comment	Qual-FocGroup	Qual-SemiStr	Mixed	Aim	Dates	InclExclCriteria	RecruitmentMethods	DataCollection	Participants	Participants_Youth	Age_mean	Gender_Percentage	Pop_Largest	SampleOther	Outcome	Comparator	RiskFactors	Intervention	MainFindings	Notes
			epidemiological data have not accurately included and measured HIV prevalence and risk among trans feminine individuals in the region. Through the lens of the Gender Affirmation Framework, we assessed demographic correlates of risk behaviour and the prevalence of HIV among trans feminine individuals in Lebanon. METHODS: Long-chain referral sampling was used to recruit 53 participants for completion of a behavioural survey and optional free rapid HIV tests. Data were collected using i nterviewer-administered questionnaires. A multivariable logistic regression model was used																	residing in the greater Beirut area.								identified as heterosexual and 17% identified as gay.<br>-43% had other responses including ‘‘bisexual,’’ ‘‘other,’’ and ‘‘don’t know/uncertain.’’<br>-Only three participants had graduated from university and over half (57%) had not completed high school.	engagement in sex work (for income of any kind) and relationship status<br>-Risky sexual behaviour: one or more instance of condomless receptive anal intercourse, history of HIV testing within last 6 months, within 12 months, experiences with discrimination, stigma, and trauma.		majority (98%) of participants reported having experienced a form of discrimination due to their transgender identity or gender presentation in the last year<br>-Two thirds (67%) of the sample reported family not being at all supportive of their transgender identity.<br>-Half (49%) of participants reported having been sexually abused or assaulted in their lifetime<br>-Being completely ‘‘open’’/ ‘‘out’’ as transgender at work or school was associated with almost six times the odds of having had condomless receptive anal intercourse * (p=0.04)*<br>-Sex work was correlated with CRAI but was		feminine population in Lebanon<br>-66%of the sample reported current sex work; sex work was correlated with CRAI but was not significant in multivariate analysis. In regression analysis, ‘‘ope nness’’/‘‘outn ess’’ about transgender identity at work or school was significantly associated with CRAI. Surprisingly, a history of sexual abuse/assault was negatively correlated with CRAI, suggesting the need for further inquiry.	

Refid	Author	Title	Abstract	User	Level	Author	Date	Jurisd	PubType	PubType_comment	Quant-CaseContr	Quant-CrossSect	Quant-Other	Quant-Other_comment	Qual-FocGroup	Qual-SemiStr	Mixed	Aim	Dates	InclExclCriteria	RecruitmentMethods	DataCollection	Participants	Participants_Youth	Age_mean	Gender_Percentage	Pop_Largest	SampleOther	Outcome	Comparator	RiskFactors	Intervention	MainFindings	Notes
			to identify demographic determinants of HIV risk behaviour. RESULTS: Fifty-seven percent of participants reported condomless receptive anal intercourse (CRAI) with male partner(s) in the last three months, 40% of whom reported not knowing the HIV status of the partner(s). Of the participants tested for HIV as part of the study or via self-report, four (10%) were HIV positive; 13 declined HIV testing. Forty percent of the sample had no prior history of HIV testing. A history of trauma such as sexual abuse/assault was reported by almost half of the participants (49%). Sixty-eight percent reported																											not significant in multivariate analysis.			

Refid	Author	Title	Abstract	User	Level	Author	Date	Jurisd	PubType	PubType_comment	Quant-CaseContr	Quant-CrossSect	Quant-Other	Quant-Other_comment	Qual-FocGroup	Qual-SemiStr	Mixed	Aim	Dates	InclExclCriteria	RecruitmentMethods	DataCollection	Participants	Participants_Youth	Age_mean	Gender_Percentage	Pop_Largest	SampleOther	Outcome	Comparator	RiskFactors	Intervention	MainFindings	Notes
			experiencing physical violence and 32% police arrest, because of gender identity or presentation. A staggering 98% reported having experienced gender identity or gender presentation-related discrimination. Sixty-six percent of the sample reported current sex work; sex work was correlated with CRAI but was not significant in multivariate analysis. In regression analysis, "openness"/"outness" about transgender identity at work or school was significantly associated with CRAI. Surprisingly, a history of sexual abuse/assault was negatively correlated with CRAI, suggesting the need for further inquiry.																															

Refid	Author	Title	Abstract	User	Level	Author	Date	Jurisd	PubType	PubType_comment	Quant-CaseContr	Quant-CrossSect	Quant-Other	Quant-Other_comment	Qual-FocGroup	Qual-SemiStr	Mixed	Aim	Dates	InclExclCriteria	RecruitmentMethods	DataCollection	Participants	Participants_Youth	Age_mean	Gender_Percentage	Pop_Largest	SampleOther	Outcome	Comparator	RiskFactors	Intervention	MainFindings	Notes
			CONCLUSIONS: The results of this study provide implications for how to address sexual health among trans feminine individuals in Lebanon and the greater Middle East and North Africa region.																															
193	G. J. Wagner, J. Tohme, M. Hoover, S. Frost, A. Ober, D. Khouri, M. Iguchi, J. Mokhbat	HIV prevalence and demographic determinants of unprotected anal sex and HIV testing among men who have sex with men in Beirut, Lebanon	The limited epidemiological data in Lebanon suggest that HIV incident cases are predominantly among men who have sex with men (MSM). We assessed the prevalence of HIV and demographic correlates of condom use and HIV testing among MSM in Beirut. Respond ent-driven sampling was used to recruit 213 participants for completion of a behavioral survey and an optional free	mohammad.ala kel	5	Wagner	2014	Beirut, Lebanon	PubPeer			Quant-CrossSect						To assess the prevalence of HIV and demographic correlates of condom use and HIV testing among MSM in Beirut.	05/2012 to 12/2012	Being biologically male and male-identified, age 18 years or older, fluent in English or Arabic, residing in greater Beirut, and having had oral or anal sex with a man in the past 12 months.	Respondent-driven sampling was used.	A survey was administered in English or Arabic with com puter-assisted interview software.	N= 213	Nearly half (47%) were under age 25 years. (approx. n=100)	Age range: 18-52, mean age = 26.2	100% Males	100% MSM	-Two-thirds (67%) self-identified as gay, and most were employed (72%) and had some university education (65%).<br>-Most were born in Lebanon (70%), Iraq (N = 37; 17%) and Syria (N = 11; 5%), many of whom lived with networks of other MSM.<br>-Muslim (32%) and Christian (35%); the remaining third were mostly atheist or having no formal religious affiliation.<br>-Ninety-one	-The number of sex partners in the past 3 months and the number of partners of each of three partner types: lover or primary partner (man/woman with whom you felt emotionally involved in a committed relationship);<br>-One-night stand or “hook-up” (man/ woman with whom you had sex only once and had no emotional attachment to); and “other” partners (man/woman with whom you	N/A	-Nearly half (N = 100; 49%) reported at least one “hook-up” or “one night stand” and 45% (N = 91) described having at least one “other” type of sex partner in the past 3 months<br>-Thirty-three (16%) men reported transactional sex with men in the past 3 months<br>-118 who had receptive anal sex, the mean number of such sex acts over the past 3 months was 12.8, of which	N/A	-.The interviewers observed that the participants who refused the HIV test tended to report high-risk behaviors in the survey and seemed to be uncomfortable when it came time to considering the HIV test, perhaps from fear of testing HIV-positive<br>-The study showed a predominance of unprotected insertive and receptive anal sex.<br>- Men in committed relationships	Related to ref id 190

Refid	Author	Title	Abstract	User	Level	Author	Date	Jurisd	PubType	PubType_comment	Quant-CaseContr	Quant-CrossSect	Quant-Other	Quant-Other_comment	Qual-FocGroup	Qual-SemiStr	Mixed	Aim	Dates	InclExclCriteria	RecruitmentMethods	DataCollection	Participants	Participants_Youth	Age_mean	Gender_Percentage	Pop_Largest	SampleOther	Outcome	Comparator	RiskFactors	Intervention	MainFindings	Notes
			rapid HIV test. Multivariate regression analysis was used to examine demographic correlates of unprotected anal sex and any history of HIV testing. Nearly half (47 %) were under age 25 years and 67 % self-identified as gay. Nearly two-thirds (64 %) reported any unprotected anal intercourse (UAI) with men in the prior 3 months, including 23 % who had unprotected anal intercourse with men whose HIV status was positive or unknown (UAIPU) to the participant. Three men (1.5 % of 198 participants tested) were HIV-positive; 62 % had any history of HIV testing prior to the study and testing was less																							participants (43%) reported currently being in a committed relationship.	had sex, but who was neither your lover nor one-night stand, such as a "fuck-buddy" or "friend with benefits").		an average of 56% were unprotected (i.e., no condom used)<br>-Men who had unprotected anal sex with partners of unknown or positive HIV status were much less likely to have ever tested for HIV compared to the rest of the sample (33% vs. 71%; p < .001)*<br>-Having at least some university education (O.R. = 2.79; p-value <0.05*) and being in a committed relationship (O.R. =2.81 <0.01*) were associated with having nearly thrice the odds of having any unprotected anal sex with men when partner HIV status was not accounted for<br>-Men in a committed relationship were 85% less		were almost 3 times more likely to engage in unprotected anal sex; however, men in relationships were much less likely to have unprotected sex with men whose HIV status was unknown.<br>Nearly three-quarters of the men in relationships reported that their partner was their only sex partner in the past 3 months, and members of a couple are more likely to know each other's HIV status.<br>-Those with any prior history of HIV testing were more likely to be in a relationship and have any university education.	

Refid	Author	Title	Abstract	User	Level	Author	Date	Jurisd	PubType	PubType_comment	Quant-CaseContr	Quant-CrossSect	Quant-Other	Quant-Other_comment	Qual-FocGroup	Qual-SemiStr	Mixed	Aim	Dates	InclExclCriteria	RecruitmentMethods	DataCollection	Participants	Participants_Youth	Age_mean	Gender_Percentage	Pop_Largest	SampleOther	Outcome	Comparator	RiskFactors	Intervention	MainFindings	Notes
			common among those engaging in UAIPU compared to others (33 % vs. 71 %). In regression analysis, men in a relationship had higher odds of having UAI but lower odds of UAIPU and any university education was associated with having UAI; those with any prior history of HIV testing were more likely to be in a relationship and have any university education. HIV prevention efforts for MSM need to account for the influence of relationship dynamics and promotion of testing needs to target high-risk MSM.																											likely to engage in unprotected anal sex with unknown status partners compared to the rest of the sample (O.R. = 0.15; p-value <0.001*)			
206	H. Sajjadi, Z. Jorjoran Shushtari, M. Shati, Y. Salimi, M. Dejman, M. Vameghi, S. Karimi, Z. Mahmoodi	An indirect estimation of the population size of students with high-risk behaviors in select universities of	BACKGROUND : Network scale-up is one of the most important indirect methods of estimating the size of clandestine	mohammad.alakel	5	Sajjadi	2018	Tehran and Alborz, Iran	PubPeer			Quant-CrossSect						To indirectly estimate the population size of students with high-risk behaviors in select universities of	Between spring and winter 2015	Having been a student for at least one year in two universities at Tehran or Alborz medical university and being of Iranian	Visited the select university campuses and used convenience sampling to select their participants.	Data were collected using a checklist. All the data about the high-risk behaviors of the students' close friends were	801	N= 253 (age 20<)<br>N= 381 (20-24)	The mean (standard deviation) age of the participants was 22.41(3.14) years.	61.8% male	56.5% reported heterosexual extramarital intercourse	61.2% of participants had a bachelor's or master's degree.	Tramadol Use, Alcohol Consumption, Opium Use, Cannabis Use, Extramarital Heterosexual Intercourse,	The likelihood of having at least one close friend with high-risk behaviours was compared across demographic	The likelihood of having a close friend who used tramadol (Pmadol (P<0.05)* consumed alcohol	N/A	Because of their higher sense of accountability, medical students experience greater stress than other	

Refid	Author	Title	Abstract	User	Level	Author	Date	Jurisd	PubType	PubType_comment	Quant-CaseContr	Quant-CrossSect	Quant-Other	Quant-Other_comment	Qual-FocGroup	Qual-SemiStr	Mixed	Aim	Dates	InclExclCriteria	RecruitmentMethods	DataCollection	Participants	Participants_Youth	Age_mean	Gender_Percentage	Pop_Largest	SampleOther	Outcome	Comparator	RiskFactors	Intervention	MainFindings	Notes
		medical sciences: A network scale-up study	populations and people with high-risk behaviors. The present study is an indirect estimation of the population size of students with high-risk behaviors in select universities of medical sciences. METHODS: A total of 801 students from two University of Medical Sciences at Tehran and Alborz University of Medical Sciences were selected through convenience sampling. Six subgroups of high-risk behaviors were examined in the study, including Tramadol use, cannabis use, opium use, alcohol consumption, extramarital heterosexual intercourse, and heterosexual															medical sciences.		nationality		obtained in an anonymous form.							Heterosexual Intercourse in Return for money	factors (ex. gender, residence, marital status)	$(P<0.01)$* and had heterosexual intercourse $(P<0.05)$* was significantly higher in the social network of the students who shared homes with other students compared to those who lived in the dormitory. Married students compared to single students had a smaller chance of having at least one close friend who consumed alcohol and had heterosexual intercourse $(P<0.05)$*. Compared to men, women had a smaller chance of having at least one close friend who used tramadol, had extramarital sex or engaged in extramarital heterosexual intercourse in return for money or no money $(P<0.001)$*.		students, and this pressure could be a reason for their greater inclination to drugs. Students who share homes with each other are more likely to belong to social networks of people with high-risk behaviors and drug abuse compared to those who live with their families. The highest frequency of friends in participants' social networks pertained to close friends with extramarital heterosexual intercourse (5.53%) and alcohol consumers (4.92%) and the lowest frequency to close friends who used opium (0.33%). Alcohol consumption and extramarital heterosexual intercourse are very common	

Refid	Author	Title	Abstract	User	Level	Author	Date	Jurisd	PubType	PubType_comment	Quant-CaseContr	Quant-CrossSect	Quant-Other	Quant-Other_comment	Qual-FocGroup	Qual-SemiStr	Mixed	Aim	Dates	InclExclCriteria	RecruitmentMethods	DataCollection	Participants	Participants_Youth	Age_mean	Gender_Percentage	Pop_Largest	SampleOther	Outcome	Comparator	RiskFactors	Intervention	MainFindings	Notes
			intercourse in return for money. To estimate the social network size in the study population, each participant was asked to name their close student friends from the two select universities. Data were collected using a checklist designed for this purpose. RESULTS: The participants' mean number of close friends from the selected medical universities was C = 8.14 (CI: 7.54-8.75). Within these social networks, friends with extramarital heterosexual intercourse (5.53%) and friends who consumed alcohol (4.92%) had the highest frequency, and friends who used opium (0.33%) had the																														among students. Special HIV prevention programs are therefore necessary for this age group.	

Refid	Author	Title	Abstract	User	Level	Author	Date	Jurisd	PubType	PubType_comment	Quant-CaseContr	Quant-CrossSect	Quant-Other	Quant-Other_comment	Qual-FocGroup	Qual-SemiStr	Mixed	Aim	Dates	InclExclCriteria	RecruitmentMethods	DataCollection	Participants	Participants_Youth	Age_mean	Gender_Percentage	Pop_Largest	SampleOther	Outcome	Comparator	RiskFactors	Intervention	MainFindings	Notes
			lowest frequency. The variables of age, gender, marital status, type of residence and academic degree were significantly related to the likelihood of having close friends with certain high-risk behaviors (P<0.001). CONCLUSION: According to the results obtained, alcohol consumption and extramarital heterosexual intercourse are very common among students. Special HIV prevention programs are therefore necessary for this age group.																															
225	F. Khalajabadi Farahani, M. M. Akhondi, M. Shirzad, A. Azin	Hiv/Sti Risk-Taking Sexual Behaviours and Risk Perception among Male University Students in Tehran: Implications for Hiv Prevention	Recent evidence indicates a rising trend in premarital sexual activity among young people in Iran. However, little is known about the	mohammad.alakel	5	Farhani	2018	Tehran, Iran	PubPeer			Quant-CrossSect						Assess HIV/STI-related sexual risk-taking behaviours (correlates and determinants) and HIV/STI risk perception among male university	10/ 2013 to 03/ 2014	Male university students in Tehran selected from both government and private universities	Respondents were selected using two-stage stratified cluster sampling.	Data was collected using a survey. The survey instrument was a pilot-tested, structured, self-administered anonymous questionnaire.	N= 1357	N= 601 (Age <20)<br>N= 349 (Age 20-23)<br>N total = 950	Mean = 22 years (standard deviation = 2.76)<br>age range= 17-38	100% males	13.4% MSM	100% students	-Inconsistent condom use at sexual intercourse over the past 30 days, number of sexual partners ever had in their lifetime<br>-HIV risk	Inconsistent condom use compared across: (age, type of university, age at sexual debut, Number of life partners, HIV knowledge) and	-Risk behaviour: low condom use was more common among government university students: Only 57% used a condom at their last intercourse,	N/A	-Early sexual debut (<18 years), studying in a private university, ever watching pornography and work experience were found to be	

Refid	Author	Title	Abstract	User	Level	Author	Date	Jurisd	PubType	PubType_comment	Quant-CaseContr	Quant-CrossSect	Quant-Other	Quant-Other_comment	Qual-FocGroup	Qual-SemiStr	Mixed	Aim	Dates	InclExclCriteria	RecruitmentMethods	DataCollection	Participants	Participants_Youth	Age_mean	Gender_Percentage	Pop_Largest	SampleOther	Outcome	Comparator	RiskFactors	Intervention	MainFindings	Notes
		among Youth	extent to which young people's sexual behaviours expose them to HIV and STI risks. This study aimed to assess HIV/STI-related sexual risk-taking behaviours (correlates and determinants) and HIV/STI risk perception among male university students in Tehran. A representative sample of male university students (N=1322) studying in government and private Tehran universities completed an anonymous questionnaire survey in 2013-14. Respondents were selected using two-stage stratified cluster sampling. About 35% of respondents had ever had premarital sex (n=462). The															students in Tehran											perception: How much were you worried about HIV infection over the last 12 months? - How much are you worried about STIs in the future?'	having multiple lifetime partners compared across: (type of university, work experience, age at sexual debut, ever watched pornography)	and this was significantly greater among men from private than government universities (63.4% vs 44.3%, p< 0.001)*<br><br>- Risk behaviour: multiple partners: The likelihood of having multiple partners among males with work experience was nearly 2.6 times greater than the corresponding rate among males with no work experience (adjusted OR = 2.58, p <0.01).* The likelihood of having multiple sex partners was 3.47 times greater among those who initiated their first sex when they were younger than 18 years, than among subjects who initiated sex when they were 20 years of age and older		significant predictors of having multiple sexual partners.<br>- This study also showed that inconsistent condom use was directly associated with having only one partner.<br>- This study revealed that inconsistent condom use among men with poor HIV knowledge (HIV mode of transmission) was 2.57 times higher than among men with good HIV knowledge.<br>-Younger age at sexual debut, having one lifetime sexual partner and poor HIV knowledge were significant predictors of inconsistent condom use over the preceding month.	

Refid	Author	Title	Abstract	User	Level	Author	Date	Jurisd	PubType	PubType_comment	Quant-CaseContr	Quant-CrossSect	Quant-Other	Quant-Other_comment	Qual-FocGroup	Qual-SemiStr	Mixed	Aim	Dates	InclExclCriteria	RecruitmentMethods	DataCollection	Participants	Participants_Youth	Age_mean	Gender_Percentage	Pop_Largest	SampleOther	Outcome	Comparator	RiskFactors	Intervention	MainFindings	Notes
			majority (about 85%) of the sexually experienced students reported having multiple sexual partners in their lifetime. More than half (54%) reported inconsistent condom use over the previous month. Despite this exposure to HIV/STI risk, the respondents had a very low level of HIV/STI risk perception. Only 6.5% were highly concerned about contracting HIV over the previous year, and an even lower percentage (3.4%) were concerned about contracting STIs in the near future. Early sexual debut (<18 years), studying in a private university, ever watching																										(adjusted OR = 3.47, p< 0.001)*. The probability of having multiple sex partners in their lifetime among those who had ever watched pornographic movies was 3.75 times the corresponding rate among those who had never watched pornography (OR = 3.75, p <0.01)*			

Refid	Author	Title	Abstract	User	Level	Author	Date	Jurisd	PubType	PubType_comment	Quant-CaseContr	Quant-CrossSect	Quant-Other	Quant-Other_comment	Qual-FocGroup	Qual-SemiStr	Mixed	Aim	Dates	InclExclCriteria	RecruitmentMethods	DataCollection	Participants	Participants_Youth	Age_mean	Gender_Percentage	Pop_Largest	SampleOther	Outcome	Comparator	RiskFactors	Intervention	MainFindings	Notes
			pornography and work experience were found to be significant predictors of having multiple sexual partners. Younger age at sexual debut, having one lifetime sexual partner and poor HIV knowledge were significant predictors of inconsistent condom use over the preceding month. HIV prevention programmes among Iranian youth need to focus on the postponement of first sex and enhancement of HIV/STI knowledge in the light of increasing access of young people to pornography.																															
248	M. Mirzaee, Y. Jahani, H. Sharifi	Reasons for Inconsistent Condom Use Found as Answers to a Multiple	BACKGROUND : The use of condoms is important for preventing Sexually	nicole.andruszkiewicz	5	Mirzaee et al.,	2012	Iran	PubPeer			Quant-CrossSect						To assess the reasons for inconsistent condom use in Iranian adults	2013	All adult youths between 19 and 29 yr, living in Iran, who had ever had extramarital	Multistage cluster sampling.	A questionnaire that consisted of demographic items, knowledge, attitude, and	N=495	N=495	52.9% (19-24); 47.1% (25-29)	74.1% Male	100% students	66.3% single 48.9% university student or graduate	-Knowledge, attitude, and practices (condom use, drug use, alcohol use)	-Compared demographic variables according to reasons for inconsistent	-The frequently selected reason for inconsistent condom use in adults aged between 19 and	N/A	(77.96%) did not use condoms with regularity. The reason frequently chosen for	

Refid	Author	Title	Abstract	User	Level	Author	Date	Jurisd	PubType	PubType_comment	Quant-CaseContr	Quant-CrossSect	Quant-Other	Quant-Other_comment	Qual-FocGroup	Qual-SemiStr	Mixed	Aim	Dates	InclExclCriteria	RecruitmentMethods	DataCollection	Participants	Participants_Youth	Age_mean	Gender_Percentage	Pop_Largest	SampleOther	Outcome	Comparator	RiskFactors	Intervention	MainFindings	Notes
		Response Question: A National Survey of Iranian Adults, 2013	Transmitted Infections (STIs). However, the prevalence of condom use is not satisfactory. The aim of this study was to assess the reasons for inconsistent condom use in Iranian adults. STUDY DESIGN: Cross-sectional study. METHODS: Data were gathered through multi-stage sampling. Participants were enrolled from 13 provinces in Iran aged between 19 and 29 years. They had ever-extramarital sex and did not use condoms regularly in their sexual contacts, asked the reasons for inconsistent condom use. RESULTS: We had 3,246 adults, from which 635 (19.5%) had																	sex—which means in lifetime having sexual contact outside formal marriage—and were not using condoms with regularity		practice towards HIV.							towards HIV -Reasons for inconsistent condom use	condom use.	24 yr and 25 and 29 yr was 'inaccessibility of condoms' (49.6%) and (48.9%). --A high percentage of females thought condom use was not necessary (49.2%), but the most important reason for not using condoms in males was that condoms were not accessible (55.9%) (P<0.0001)* -Men reported the reasons for inconsistent condom use more than females in the choices: 'inaccessibility of condoms' and 'do not like the use of condoms'		inconsistent condom use was 'that it is not accessible' (49.3%). The differences between the categories of some variables, according to the choice of reasons for inconsistent condom use, were significant (P<0.05): age, gender, knowledge of HIV, attitude towards HIV, knowing infected HIV person and alcohol or stimulant(s) used before sexual contact. According to multivariate multilevel logistic, the effect of gender was significant on most of reasons for inconsistent condom use (P<0.05).	

Refid	Author	Title	Abstract	User	Level	Author	Date	Jurisd	PubType	PubType_comment	Quant-CaseContr	Quant-CrossSect	Quant-Other	Quant-Other_comment	Qual-FocGroup	Qual-SemiStr	Mixed	Aim	Dates	InclExclCriteria	RecruitmentMethods	DataCollection	Participants	Participants_Youth	Age_mean	Gender_Percentage	Pop_Largest	SampleOther	Outcome	Comparator	RiskFactors	Intervention	MainFindings	Notes
			ever-extramarital sex. Among them, 495 (77.96%) did not use condoms with regularity. The reason frequently chosen for inconsistent condom use was 'that it is not accessible' (49.3%). The differences between the categories of some variables, according to the choice of reasons for inconsistent condom use, were significant (P<0.05): age, gender, knowledge of HIV, attitude towards HIV, knowing infected HIV person and alcohol or stimulant(s) used before sexual contact. According to multivariate multilevel logistic, the effect of gender was significant on most of																																

Refid	Author	Title	Abstract	User	Level	Author	Date	Jurisd	PubType	PubType_comment	Quant-CaseContr	Quant-CrossSect	Quant-Other	Quant-Other_comment	Qual-FocGroup	Qual-SemiStr	Mixed	Aim	Dates	InclExclCriteria	RecruitmentMethods	DataCollection	Participants	Participants_Youth	Age_mean	Gender_Percentage	Pop_Largest	SampleOther	Outcome	Comparator	RiskFactors	Intervention	MainFindings	Notes
			reasons for inconsistent condom use (P <0.05). CONCLUSIONS: Most of the reasons that were selected for inconsistent condom use were inaccessibility and not knowing that its use is essential. Hence, it is important to improve the knowledge of adults regarding STI/HIV and extend the locations of condom distribution. We should try to promote the culture of condom use as a routine.																															
279	R. Heimer, R. Barbour, D. Khouri, F. W. Crawford, F. Shebl, E. Aaraj, K. Khoshnood	HIV Risk, Prevalence, and Access to Care Among Men Who Have Sex with Men in Lebanon	Little is known about HIV prevalence and risk among men who have sex with men (MSM) in much of the Middle East, including Lebanon. Recent national-level surveillance has	mohammad.alakel	5	Heimer	2017	Beirut, Lebanon	PubPeer			Quant-CrossSect						To examine HIV Risk, Prevalence, and Access to Care Among Men Who Have Sex with Men in Lebanon	10/2014 to 02/2015	Eligibility criteria for participation included being male and 18 years of age or older, self-report of sex with another man in 6 months before enrollment, and being selected as a seed or having a valid	MSM were recruited by respondent-driven sampling	The interview questionnaire was designed to obtain information on participants' sociodemographic situation, sexual behaviors, alcohol and drug use, health, HIV testing and	N= 292	. -Approx. 75% aged 30 or younger (approx. n=219)	-Mean – SD 27.1<br>-Median (IQR) - 25 (21, 30)	100% Male	100% MSM	-71% was born in Lebanon and a quarter was born in Syria<br>-90% single<br>-60% employed or earning income	-Ever had vaginal or anal sex with a woman<br>-Ever had anal sex with a man<br>-HIV prevalence (self-reported or voluntary test), HIV risk behaviors, psychological factors	-Compared factors associated with prevalent HIV infection between HIV+ and HIV- men who have sex with men.	-More than one in five MSM (n = 61, 21.6%) reported engaging in group sex during the year before interview<br>-Nearly half (n = 30, 49.2%) never used a condom and only 10 (16.4%)	N/A	The main findings from our study of MSM in Lebanon are (1) a higher HIV-1 prevalence than in any previous study, (2) the existence of a large number of Syrian-born recently arrived	

Refid	Author	Title	Abstract	User	Level	Author	Date	Jurisd	PubType	PubType_comment	Quant-CaseContr	Quant-CrossSect	Quant-Other	Quant-Other_comment	Qual-FocGroup	Qual-SemiStr	Mixed	Aim	Dates	InclExclCriteria	RecruitmentMethods	DataCollection	Participants	Participants_Youth	Age_mean	Gender_Percentage	Pop_Largest	SampleOther	Outcome	Comparator	RiskFactors	Intervention	MainFindings	Notes
			suggested an increase in HIV prevalence concentrated among men in Lebanon. We undertook a biobehavioral study to provide direct evidence for the spread of HIV. MSM were recruited by res pondent-driven sampling, interviewed, and offered HIV testing anonymously at sites located in Beirut, Lebanon, from October 2014 through February 2015. The interview questionnaire was designed to obtain information on participants' soci odemographic situation, sexual behaviors, alcohol and drug use, health, HIV testing and care, and experiences of stigma and discrimination. Individuals not reporting an HIV diagnosis were																	study coupon		care, and experiences of stigma and discrimination.							associated with prevalent infection among MSM.<br>-Secondary outcomes included factors associated with receiving HIV care among those MSM who reported being HIV positive and differences between Lebanese-born and foreign-born MSM.		used a condom for all intercourse<br>-Alcohol and drug use data were available for 48 of the 61 group sex events. Alcohol was available at 45 (93.8%), stimulants such as methamphetamine were available at 7 (14.6%), club drugs were available at 9 (18.8%), and multiple substances were available at 16 (33.3%) events<br>-Online searching for partners, using either a mobile app or the Internet, was reported by 173 (59.7%) MSM. Among the 70% who reported multiple partners in the 6-month survey window, 70% used electronic tools to find partners.<br>-Only 5 (1.7%) had ever		MSM, and (3) HIV risk behaviors concentrated among HIV-negative MSM that are centered around the use of social media for partner acquisition and participation in group sexual encounters.	

Refid	Author	Title	Abstract	User	Level	Author	Date	Jurisd	PubType	PubType_comment	Quant-CaseContr	Quant-CrossSect	Quant-Other	Quant-Other_comment	Qual-FocGroup	Qual-SemiStr	Mixed	Aim	Dates	InclExclCriteria	RecruitmentMethods	DataCollection	Participants	Participants_Youth	Age_mean	Gender_Percentage	Pop_Largest	SampleOther	Outcome	Comparator	RiskFactors	Intervention	MainFindings	Notes
			offered optional, anonymous HIV testing. Among the 292 MSM recruited, we identified 36 cases of HIV (12.3%). A quarter of the MSM were born in Syria and recently arrived in Lebanon. Condom use was uncommon; 65% reported condomless sex with other men. Group sex encounters were reported by 22% of participants. Among the 32 individuals already aware of their infection, 30 were in treatment and receiving antiretroviral therapy. HIV prevalence was substantially increased over past estimates. Efforts to control future increases will have to focus on reducing specific risk behaviors and experience of																												injected drugs and only one had done so in the 6 months before interview. -111 participants (40%) reported having sex while using drug or high in the last year			

Refid	Author	Title	Abstract	User	Level	Author	Date	Jurisd	PubType	PubType_comment	Quant-CaseContr	Quant-CrossSect	Quant-Other	Quant-Other_comment	Qual-FocGroup	Qual-SemiStr	Mixed	Aim	Dates	InclExclCriteria	RecruitmentMethods	DataCollection	Participants	Participants_Youth	Age_mean	Gender_Percentage	Pop_Largest	SampleOther	Outcome	Comparator	RiskFactors	Intervention	MainFindings	Notes
			stigma and abuse, especially among Syrian refugees.																															
343	M. Maarefvand, B. Kassaie, H. Ghiasvand, R. Abolfathi Gharibdoosti, J. Khubchandani	Sexual and Drug Use Risk Behaviors of Internal Long Distance Truck Drivers in Iran	BACKGROUND: Long Distance Truck Drivers (LDTDs) and their sexual health risk behaviors have been associated with greater prevalence of sexually transmitted Infections (STIs), HIV and hepatitis virus transmission. However, there is no information about Iranian LDTDs' high-risk behaviors. The aim of this investigation was to estimate the prevalence of high-risk behaviors in Iranian LDTDs. METHODS: This cross-sectional study was conducted in Jun 2014 with LDTDs from Tehran Province of Iran. LDTDs were recruited via convenience	mohammad.alakel	5	Maarefvand	2016	Tehran, Iran	PubPeer			Quant-CrossSect						To estimate the prevalence of high-risk behaviors in Iranian long distance truck drivers	Data was collected for a week in Jun 2014	Long-distance truck drivers were eligible for the study if they spent at least 3 days away from home in a week	Long-distance truck drivers were selected via a convenience sample.	Demographic and behavioral in-formation were collected by same-sex interviewer using semi-structured questionnaire with eligible Long-distance truck drivers in Tehran Province trucks parking lot.	N=349 long-distance truck drivers	N= 114 (age = 19-32)	The mean age of 36.91 yr (range, 19–65 yr)	Not reported	14 participants (4%) reported having had same sex background (does not identify if all are MSM)	33.8% reported drug use history	Days away from home in week because of working travels, female sexual partners (wife and extramarital sexual relations (including paid and non-paid partners)) and number of sexual contacts in last month), male sexual partners and number of homosexual contacts in last month, know homosexual colleagues, number of condom use during sexual contacts in last month, drug abuse background (including drug abuse duration, types of abused drugs, intravenous drug injection, needle sharing, imprisonment related to drug	Comparing extramarital relations without a condom across demographics (age, education, marital status, days away from home)	Age was associated with participation in risk behaviours. Younger long distance truck drivers had more condom use (protective behaviours) but also had more extramarital partners (high risk behaviours) than older drivers:<br><br>Condom use: Younger long-distance truck drivers reported more condom use with their partners (r=-0.170, P?0.001)* and condom use in their extramarital sex contacts (including with sex workers) (r=-0.176, P?0.001)*. Long-distance truck drivers who reported more contacts with	N/A	Iranian long-distance truck drivers have specific risk factors for unhealthy sexual behaviors. HIV and STI prevention program among long distance truck drivers needs to focus more on those who are 33-40 yr old and live in provinces other than developing provinces. More efforts (e.g., counseling and awareness raising among truckers about being at risk of acquiring STI and HIV, etc.) are required to make truckers motivated and able to use condom each time they have sex with nonregular partners.	

Refid	Author	Title	Abstract	User	Level	Author	Date	Jurisd	PubType	PubType_comment	Quant-CaseContr	Quant-CrossSect	Quant-Other	Quant-Other_comment	Qual-FocGroup	Qual-SemiStr	Mixed	Aim	Dates	InclExclCriteria	RecruitmentMethods	DataCollection	Participants	Participants_Youth	Age_mean	Gender_Percentage	Pop_Largest	SampleOther	Outcome	Comparator	RiskFactors	Intervention	MainFindings	Notes
			sampling and given a 43-item reliable and valid questionnaire to assess sexual health risk behaviors and demographic and background characteristics of study participants. RESULTS: A total of 349 LDTDs with the mean age of 36.91 yr (range, 19-65 yr) participated in the study. The average duration of staying away from home for participants was 5 d (SD=+/-1). Majority of the LDTDs were married (82.2 %) and had more than 5 yr (inclusive) of formal education (95.7%). Younger LDTDs reported more condom use with their partners (r=-0.170, P<=0.001), more																										abuse or drug dealing, and treatment)		sex workers were more likely to use condom (r=0.566, P?0.001)*. Long-distance truck drivers who spent more days away from home were more likely not to use condom (r=-0.141, P?0.001)*.<br><br># of partners: Younger drivers have more extra-marital sexual contacts (r=-0.157, P?0.001)*, Money for sex: More young drivers pay for sex (r=-0.110, P?0.005)*			

Refid	Author	Title	Abstract	User	Level	Author	Date	Jurisd	PubType	PubType_comment	Quant-CaseContr	Quant-CrossSect	Quant-Other	Quant-Other_comment	Qual-FocGroup	Qual-SemiStr	Mixed	Aim	Dates	InclExclCriteria	RecruitmentMethods	DataCollection	Participants	Participants_Youth	Age_mean	Gender_Percentage	Pop_Largest	SampleOther	Outcome	Comparator	RiskFactors	Intervention	MainFindings	Notes
			extramarital sexual contacts (r=-0.157, P<=0.001), more pay for sex (r=-0.110, P<=0.005) and condom use in their extra-marital sex contacts (including with sex workers) (r=-0.176, P<=0.001). CONCLUSION: Iranian LDTDs have specific risk factors for unhealthy sexual behaviors. Prevention efforts must emphasize on specific high-risk groups.																															
355	M. Noroozi, E. A. Merghati Khoei, F. Taleghani, M. Tavakoli, A. Gholami	How does a group of Iranian youth conceptualize their risky sexual experiences?	BACKGROUND: There are restrictions as well as lawful and religious prohibition for sexual relationships in Iranian society. OBJECTIVES: This study explores the varied experiential reality of Iranian youth and their	nicole.andruszkiewicz	5	Noroozi et al.,	2015	Isfahan, Iran	PubPeer							Qual-SemiStr		To explore the varied experiential reality of Iranian youth risky sexual experiences.	01/2012-02/2013	Single men and women aged 18-24 living in Isfahan.	Potential participants were recruited from the university campus, parks, and gyms.	Face-to-face in-depth semi-structured interviews were used.	N=30	N=30	Mean=21.9 Range=(18-24)	57% Female	100% students	Not reported	Questions about the beginning of sexual relations, sexual activities, and the youth perceptions and beliefs about risky sex, HIV/AIDS and STIs, protective behaviors.	N/A	-Males did not not use condoms consistently because they had not seen themselves at risk of STIs or HIV as well as their belief about the decrease of sexual pleasure. -Mostly females are not interested in	N/A	Women seldom asked for condom use due to limited knowledge about STIs-HIV/AIDS and unpleasant experiences with condoms. Men had limited knowledge about various sexual modes of HIV and STIs transmission	

Refid	Author	Title	Abstract	User	Level	Author	Date	Jurisd	PubType	PubType_comment	Quant-CaseContr	Quant-CrossSect	Quant-Other	Quant-Other_comment	Qual-FocGroup	Qual-SemiStr	Mixed	Aim	Dates	InclExclCriteria	RecruitmentMethods	DataCollection	Participants	Participants_Youth	Age_mean	Gender_Percentage	Pop_Largest	SampleOther	Outcome	Comparator	RiskFactors	Intervention	MainFindings	Notes
			risky sexual experiences. PATIENTS AND METHODS: Data collection is based on semi-structured interviews with 30 single young men and women. Observation of interactions of youth in different settings has been another source of information. Interviews were microscopically content analyzed. RESULTS: The accounts of risky sexual experiences included gendered behavioral, cultural, and cognitive factors. In the re-contextualizing process, these accounts were categorized by two main themes: "gendered sexual script" and "cultural scenarios of interpersonal interactions".																												condom usage in oral sex. This is because condoms taste like rubber or an indication of decreased sexual pleasure for males; and females are also not interested in the rubber taste. -Participants said that most males do not use condom in anal sex either with their steady or casual partners.		and did not use condoms consistently because they had not seen themselves at risk of STIs or HIV as well as their belief about the decrease of sexual pleasure.	

Refid	Author	Title	Abstract	User	Level	Author	Date	Jurisd	PubType	PubType_comment	Quant-CaseContr	Quant-CrossSect	Quant-Other	Quant-Other_comment	Qual-FocGroup	Qual-SemiStr	Mixed	Aim	Dates	InclExclCriteria	RecruitmentMethods	DataCollection	Participants	Participants_Youth	Age_mean	Gender_Percentage	Pop_Largest	SampleOther	Outcome	Comparator	RiskFactors	Intervention	MainFindings	Notes
			The notion of "male power" was evident in the responses provided by the participants. Women seldom asked for condom use due to limited knowledge about STIs-HIV/AIDS and unpleasant experiences with condoms. Men had limited knowledge about various sexual modes of HIV and STIs transmission and did not use condoms consistently because they had not seen themselves at risk of STIs or HIV as well as their belief about the decrease of sexual pleasure. CONCLUSIONS: Rising youth awareness, creating a positive attitude, and appropriate knowledge about safe sex practices are seminal. It is																																	

Refid	Author	Title	Abstract	User	Level	Author	Date	Jurisd	PubType	PubType_comment	Quant-CaseContr	Quant-CrossSect	Quant-Other	Quant-Other_comment	Qual-FocGroup	Qual-SemiStr	Mixed	Aim	Dates	InclExclCriteria	RecruitmentMethods	DataCollection	Participants	Participants_Youth	Age_mean	Gender_Percentage	Pop_Largest	SampleOther	Outcome	Comparator	RiskFactors	Intervention	MainFindings	Notes
			also vital to ensure access to gender specific sexual and reproductive health services for youths, empower women, and strengthen their sexual assertiveness as well as the elimination of all forms of gender-based inequalities.																															
358	M. R. Hedayati-Moghaddam, I. Eftekharzadeh-Mashhadi, F. Fathimoghadam, S. J. Pourafzali	Sexual and Reproductive Behaviors among Undergraduate University Students in Mashhad, a City in Northeast of Iran	BACKGROUND: The incidence of sexual transmitted infections (STIs) and HIV/AIDS is globally higher in young people. This study evaluated the prevalence of sexual reproductive behaviors among undergraduate students of Mashhad, Iran. METHODS: The study was conducted on 605 students in twelve non-medical faculties of a	nicole.andruszkiewicz	5	Hedayati-Moghaddam et al.,	2014	Mashahd, Iran	PubPeer			Quant-CrossSect						To evaluate the prevalence of sexual reproductive behaviors among undergraduate students of Mashhad, Iran.	05/2008-06/2008	Undergraduate student at the Unviersity of Mashhad under the age of 25.	In each faculty, the students were classified based on four different admission years and one study field was randomly chosen from each admission year.	Using an anonymous self-administered questionnaire.	N=605	N=605	Mean (SD)= 20.8 (1.5); Range= 18-25	71.4% female.	100% students.	85.3% single.	Lifetime and current history of sexual contacts, age at first sex, number of partners, using condoms during the last sexual contact and history of AIDS education at the university during the last year.	N/A	-The lifetime prevalence of sexual relationship in males was significantly higher than females (32.9% vs. 7.6%, p<0.001)*.<br>-Students with a history of sexual contact were older than other ones (21.7±1.5 vs. 20.8±1.5 years; p<0.001)*.<br>-Only 26/64 (40.6%) of students stated using condom in their last sexual relation.	N/A	-The mean age of first sexual experience was 23.7 years old (95% CI, 23.4-24.0) with a significant difference between both sexes (p<0.001).<br>-35% (28/80) of the students including 21 males and 7 females declared to have a same-sex experience.<br>- In single sexually experienced students, the mean age at first sex was	

Refid	Author	Title	Abstract	User	Level	Author	Date	Jurisd	PubType	PubType_comment	Quant-CaseContr	Quant-CrossSect	Quant-Other	Quant-Other_comment	Qual-FocGroup	Qual-SemiStr	Mixed	Aim	Dates	InclExclCriteria	RecruitmentMethods	DataCollection	Participants	Participants_Youth	Age_mean	Gender_Percentage	Pop_Largest	SampleOther	Outcome	Comparator	RiskFactors	Intervention	MainFindings	Notes
			great university of Mashhad. A self-administered questionnaire was completed on demographic information, sexual contact in the lifetime and during the last three months, and age of first sex. Kaplan-Meier statistic was used to calculate the mean age of initiation of sex. A p < 0.05 was considered statistically significant. RESULTS: After exclusion of individuals over 25 years of age, among 590 students with a mean age of 20.8+/-1.5 years included in the analysis, 71.4% were female and 85.3% were single. Prevalence of at least one sexual contact in life was 15.1% and 35.3% of single sexually experienced																														17.6±3.3 years, 24% started sexual activity at <15 years, 34.3% had at least 3 partners and only 40.6% stated using condom in their last sex.	

Refid	Author	Title	Abstract	User	Level	Author	Date	Jurisd	PubType	PubType_comment	Quant-CaseContr	Quant-CrossSect	Quant-Other	Quant-Other_comment	Qual-FocGroup	Qual-SemiStr	Mixed	Aim	Dates	InclExclCriteria	RecruitmentMethods	DataCollection	Participants	Participants_Youth	Age_mean	Gender_Percentage	Pop_Largest	SampleOther	Outcome	Comparator	RiskFactors	Intervention	MainFindings	Notes
			students reported to have sex in the last three months. The lifetime prevalence of sexual relationship in males was significantly higher than females (32.9% vs. 7.6%, p < 0.001). The mean age of first sexual experience was 23.7 years with a significant difference between both sexes (p < 0.001). In single sexually experienced students, the mean age at first sex was 17.6+/-3.3 years, 24% started sexual activity at <15 years, 34.3% had at least 3 partners and only 40.6% stated using condom in their last sex. CONCLUSION: Although very small proportion of females reported																															

Refid	Author	Title	Abstract	User	Level	Author	Date	Jurisd	PubType	PubType_comment	Quant-CaseContr	Quant-CrossSect	Quant-Other	Quant-Other_comment	Qual-FocGroup	Qual-SemiStr	Mixed	Aim	Dates	InclExclCriteria	RecruitmentMethods	DataCollection	Participants	Participants_Youth	Age_mean	Gender_Percentage	Pop_Largest	SampleOther	Outcome	Comparator	RiskFactors	Intervention	MainFindings	Notes
			premarital sex, a significant minority of male students experienced sexual and risky behaviors. Therefore, the use of educational programs on related issues to reduce the risk of STIs/HIV among youth including university students seems to be a necessity.																															
379	M. Shokoohi, M. R. Baneshi, A. A. Haghdoost	Size Estimation of Groups at High Risk of HIV/AIDS using Network Scale Up in Kerman, Iran	OBJECTIVE: To estimate the size of groups at high risk of HIV, Network Scale UP (NSU), an indirect method, was used. METHODS: 500 Kermanian male aged 18 to 45 were recruited. 8 groups at high risk of HIV were defined: Users of opium, unknown drug, ecstasy, and alcohol; intra-venous drug users (IDUs; males who have extra-marital	mohammad.alakel	5	Shokoohi	2012	Kerman, Iran	PubPeer			Quant-CrossSect						To provide an estimate for the size of groups at high risk of HIV/AIDS in Kerman city, focusing on adult males.	Not reported	Males between 18 and 45 years old who lived in Kerman at least in the past 5 years	4 trained interviewers were approached for 500 participants through an adaptive purposive sampling. Samples were selected from college campuses, city streets and in work places.	Interviewers filled the questionnaires in face to face interviews.	N= 500	66.67% aged between 18 and 25 (n=333)	500 Kermanian males aged 18 to 45	100% Males	13.7% reported alcohol use; 13% reported opium use	50% of the subjects had an academic education	Definition for the 8 sub-populations was at least 1 use of drugs or 1 sexual contact in the last year. We defined C as the size of active social network, which means the number of male between 18 and 45 year old acquaintances (colleagues, relatives, friends) that each person knows. Estimated sizes of groups at risk	N/A	Based on the results of the probability method, the size of males with risky behaviors was considerable, particularly in using illegal drugs (opium users 13.1%, ecstasy 4.3%, alcohol users 13.7%, and IDU 1.2%). Nevertheless, the size of men who have sex with female sex workers, men who have sex with men, and	N/A	The frequency of drug use and extra-marital sexual contact among young and mid-age males in Kerman was relatively high. Based on the probability method, 13.7% of males used alcohol at least once in last year; the corresponding percent for opium was 13.1%. In addition, 12% has extra-marital sex in	

Refid	Author	Title	Abstract	User	Level	Author	Date	Jurisd	PubType	PubType_comment	Quant-CaseContr	Quant-CrossSect	Quant-Other	Quant-Other_comment	Qual-FocGroup	Qual-SemiStr	Mixed	Aim	Dates	InclExclCriteria	RecruitmentMethods	DataCollection	Participants	Participants_Youth	Age_mean	Gender_Percentage	Pop_Largest	SampleOther	Outcome	Comparator	RiskFactors	Intervention	MainFindings	Notes
			sex with females (MSF); male who have sex with female sex workers (MFSW); and male who have sex with other male (MSMs). We asked respondents whether they know anybody (probability method), and if yes, how many people (frequency method) in our target groups. RESULTS: Estimates derived in the probability method were higher than the frequency method. Based on the probability method, 13.7% (95% CI: 11.3%, 16.1%) of males used alcohol at least once in last year; the corresponding percent for opium was 13.1% (95% CI: 10.9%, 15.3%). In addition, 12% has extra-marital sex in																										were completed using a probability method (if they know anyone) and a frequency method (how many people they know).		men who have extra-marital sex with females was also noticeable (7%, 2.5%, and 12%).		last year while 7% had sex with a female sex worker.	

Refid	Author	Title	Abstract	User	Level	Author	Date	Jurisd	PubType	PubType_comment	Quant-CaseContr	Quant-CrossSect	Quant-Other	Quant-Other_comment	Qual-FocGroup	Qual-SemiStr	Mixed	Aim	Dates	InclExclCriteria	RecruitmentMethods	DataCollection	Participants	Participants_Youth	Age_mean	Gender_Percentage	Pop_Largest	SampleOther	Outcome	Comparator	RiskFactors	Intervention	MainFindings	Notes
			last year (95% CI: 10%, 14%); while 7% (95% CI: 5.8%, 8.2%) had sex with a female sex worker. CONCLUSION: We showed that drug use is more common among young and mid-age males; although their sexual contacts were also considerable. These percentages show that special preventive program is needed to control an HIV transmission. Estimates derived from probability method were comparable with data from external sources. The underestimation in frequency method might be due to the fact that respondents are not aware of sensitive characteristics																															

Refid	Author	Title	Abstract	User	Level	Author	Date	Jurisd	PubType	PubType_comment	Quant-CaseContr	Quant-CrossSect	Quant-Other	Quant-Other_comment	Qual-FocGroup	Qual-SemiStr	Mixed	Aim	Dates	InclExclCriteria	RecruitmentMethods	DataCollection	Participants	Participants_Youth	Age_mean	Gender_Percentage	Pop_Largest	SampleOther	Outcome	Comparator	RiskFactors	Intervention	MainFindings	Notes
			of all those in their network and underreporting is likely to occur.																															
412	I. Maatouk	Sexual behavior and condom use among a population of men who have sex with men attending a der matology-sexually transmitted infection clinic in Beirut-Lebanon		nicole.andruszkiewicz	5	Maatouk et al.,	2016	Beirut, Lebanon	PubPeer			Quant-CrossSect						To examine high risk behavior among men who have sex with men in Beirut, Lebanon.	June 2015	Men who have sex with men that attended the dermatology-sexually transmitted infection clinic.	MSM who attended the clinic for a medical consultation were invited.	A questionnaire was used for the study.	N=31	N=28 (90%)	not reported (range=0-35)	100% male	100% MSM	68% had a previous STI	Numbers of sexual partners, condom use, use of cell phone dating applications, use of alcohol and/or drugs with sex, previous sexually transmitted infections including HIV infection, frequency of testing for sexually transmitted infections and subjective assessment of risk behaviour.	N/A	-Eighteen (58.1%) participants had unprotected anal sex.<br>-Most of our patients (45.2%) fell in the category of 2–5 partners for the last 6 months.<br>-Alcohol and/or drugs before/during sex (denied in 58.1%).	N/A	-41.9% used alcohol and/or drugs before/during sex .<br>-41.9% used a condom every time in the last 10 times they had intercourse.<br>-16.1% never used a condom every time in the last 10 times they had intercourse.	
459	B. Honarvar, F. Salehi, R. Barfi, Z. Asadi, H. Honarvar, N. Odoomi, N. Arefi, K. B. Lankarani	Attitudes Toward and Experience of Singles with Premarital Sex: A Population-Based Study in Shiraz, Southern Iran	The population of Iran is young and millions of youths are at risk for unprotected sexual relationships and their consequences. This questionnaire-based study was conducted in Shiraz, southern Iran.	mohammad.alakel	5	Honarvar et al	2016	Shiraz, Southern Iran	PubPeer			Quant-CrossSect						To examine the attitudes of singles in Iran towards premarital sex and to assess their own experiences and risky behaviors.	02/ 2012 to 11/ 2013	Respondents were selected if they were single, lived in that zone (where they were being approached), and agreed to participate	Multistage and convenient sampling was used. Respondents from each municipal zone in Shiraz were approached in public to take part in an open-ended questionnaire.	To collect data interviewers completed the anonymous, coded questionnaires based on the answers of the interviewees which were scored.	A total of N= 1076 participants	Of 1076, 50 (4.6%) belonged to the age group of 14–17 years, 885 (82.2%) were aged between 18 and 29 years.<br>Total number (14-29)= 935	-The mean age of 24±5.8 years participated in this study.<br>-Age range = 14-71	634 males (58.9%)	41.9% of participants self-identified as heterosexual	317 (71.4%) claimed strong or very strong religious belief, 673 (62.4%) had university-level education, 235 (21.8%) did not have a job	Pattern of consensual sexual relationships: (homosexuality, bisexuality, or heterosexuality), age at first sexual contact, number of partners. Risky behaviours: cigarette smoking, alcohol	Compared demographics, risk behaviors, and knowledge about STIs and HIV between those who had premarital sex versus those who did not.	-Median age at first pre-marital sex was 18 years, and the mean age was 17.5±4.5 years.<br>-The mean age at first sexual contact was 17 years for males, and 19 for females ($p<0.001$)*<br>-The mean age at first sexual	N/A	- This study revealed that two-thirds of the singles in our study sample who engaged in premarital sex did not use a condom consistently.<br>-Gender, level of education, having a job, religious beliefs, smoking,	

Refid	Author	Title	Abstract	User	Level	Author	Date	Jurisd	PubType	PubType_comment	Quant-CaseContr	Quant-CrossSect	Quant-Other	Quant-Other_comment	Qual-FocGroup	Qual-SemiStr	Mixed	Aim	Dates	InclExclCriteria	RecruitmentMethods	DataCollection	Participants	Participants_Youth	Age_mean	Gender_Percentage	Pop_Largest	SampleOther	Outcome	Comparator	RiskFactors	Intervention	MainFindings	Notes
			Singles were asked about premarital sex (PMS) and sexual health issues. A total of 1076 participants (634 males, 58.9%) with a mean age of 24 +/- 5.8 years participated in this study. One out of 2 singles reported PMS and 1 out of 2 singles with PMS reported multiple partners. Median age at first sexual contact was 18 years. Of all singles, 452 (41.9%) were heterosexual, 61 (5.6%) were bisexual, 366 (33.9%) were alcohol users, 252 (23.3%) were smokers, 57 (5.2%) were opium users, and 392 (36.3%) did not know about preventive methods for HIV. Of 528 singles who had PMS, 126																										consumption, opium use, premarital sex and knowledge about AIDS and other STIs.		contact was 17.9±4.4 years for heterosexual contact, 16±2.8 years for homosexual contact and 14.7±4.4 years for bisexual contact (p<.001)*<br>-In 452 heterosexual respondents, the number of males (213 of 367; 58%) reported multiple partners more than females (16 of 84; 19%) (p<.001)*<br>-Regression analysis showed that alcohol use was the strongest predictor of pre-marital sex in singles followed by lack of religious beliefs		alcohol use, opium use, knowledge about HIV/STDs, and condom use were significantly related to pre-marital sex. Alcohol use was the strongest predictor of pre-marital sex in singles, followed by lack of religious beliefs.	

Refid	Author	Title	Abstract	User	Level	Author	Date	Jurisd	PubType	PubType_comment	Quant-CaseContr	Quant-CrossSect	Quant-Other	Quant-Other_comment	Qual-FocGroup	Qual-SemiStr	Mixed	Aim	Dates	InclExclCriteria	RecruitmentMethods	DataCollection	Participants	Participants_Youth	Age_mean	Gender_Percentage	Pop_Largest	SampleOther	Outcome	Comparator	RiskFactors	Intervention	MainFindings	Notes
			(23.8%) never used a condom, 223 (42.2%) used it inconsistently, and 59 (11.1%) used it mainly against sexually transmitted diseases. In the regression analysis, alcohol use was the strongest associated factor of PMS in singles (OR 4.9, 95% CI 3.3-7.4), followed by lack of religious beliefs (OR 2.3, 95% CI 1.4-3.8). As a result, the PMS situation in our setting is cause for alarm and to protect singles against the risks associated with PMS, a multidisciplinary intervention including improving access to sexual behavioral counseling centers, education about sexual health and especially condom use,																															

Refid	Author	Title	Abstract	User	Level	Author	Date	Jurisd	PubType	PubType_comment	Quant-CaseContr	Quant-CrossSect	Quant-Other	Quant-Other_comment	Qual-FocGroup	Qual-SemiStr	Mixed	Aim	Dates	InclExclCriteria	RecruitmentMethods	DataCollection	Participants	Participants_Youth	Age_mean	Gender_Percentage	Pop_Largest	SampleOther	Outcome	Comparator	RiskFactors	Intervention	MainFindings	Notes
			abstinence from alcohol use, and commitment to religious values is urgently needed to be established by health policymakers.																															
471	S. Esmaeilzadeh, H. Allahverdipour, B. Fathi, S. Shirzadi	Risk Perception of HIV/AIDS and Low Self-Control Trait: Explaining Preventative Behaviors Among Iranian University Students	BACKGROUND : In spite of developed countries there are progressive trend about HIV/AIDS and its' aspects of transmission in the low socio-economic societies. The aim of this was to explain the youth's behavior in adopting HIV/AIDS related preventive behaviors in a sample of Iranian university students by emphasizing on fear appeals approaches alongside examining the role of self-control trait for explaining adoption on danger or fear	mohammad.alakel	5	Esmaeilzadeh et al	2015	Jolfa, Iran	PubPeer			Quant-CrossSect						To explain the youth's behavior in adopting HIV/AIDS-related preventive behaviors in a sample of Iranian university students by emphasizing on fear appeals approaches (to arouse fear in order to divert behavior through the threat of impending danger or harm.)	Only the year "2013" is provided	All <28 year old students who registered for full-term courses and had no history of mental disorders.	156 randomly selected non-medical students from among two universities of Jolfa and Hadishahr in north-west of Iran	Using self-reported questionnaires given to 217 students (61 questionnaires were discarded due to incomplete responses) total 156 completed questionnaires	N= 156	N= 156	The mean± SD of 22.15 ± 2.07 years	64.7% male	32.6% reported having unsafe sex	28.8% had a history of smoking	History of high risk behaviors included having multi-sex partners, having unsafe sex, refusal of proposed sexual behavior, tattoos, smoking, alcohol consumption, drug use, and company of friends who were addicted to drug use. Self-control was measured by a modified set of 5 items scale based on a self-control scale coded from 1 to 7.	Compared self-perceptions among participants as predictors of multi-sex partners and compared self-control and EPPM theoretical variables as predictors of alcohol use	Barrier to risk behavior: High self-efficacy was a strong predictor of condom use in the high risk sexual relationships (P-value =0.03)* High self-control decreased changes of having a multiple sex partners by 13% (P-value = 0.04)*<br><br>Facilitates risk behavior: 22.5% of the respondents with low self-control and 4.7% with high self-control stated that they had multi-sex partners (p-value = 0.002)*, 23.1% of the low self-control students had	N/A	31.3% of participants were in the fear control process versus 68.7% in danger control about HIV/AIDS . There was a presence of multi-sex partners and amphetamine consumption among the participants. Low self-control trait and low perceived susceptibility were significantly related to having a history of multi-sex partners while a high level of self-efficacy significantly increased the probability of condom use.	

Refid	Author	Title	Abstract	User	Level	Author	Date	Jurisd	PubType	PubType_comment	Quant-CaseContr	Quant-CrossSect	Quant-Other	Quant-Other_comment	Qual-FocGroup	Qual-SemiStr	Mixed	Aim	Dates	InclExclCriteria	RecruitmentMethods	DataCollection	Participants	Participants_Youth	Age_mean	Gender_Percentage	Pop_Largest	SampleOther	Outcome	Comparator	RiskFactors	Intervention	MainFindings	Notes
			control processes based on Extended Parallel Process Model (EPPM). METHODS: A sample of 156 randomly selected university students in Jolfa, Iran was recruited in a predictive cross-sectional study by application of a researcher-designed questionnaire through self-report data collection manner. Sexual high risk behaviors, the EPPM variables, self-control trait, and general self-efficacy were measured as theoretical framework. RESULTS: Findings indicated that 31.3% of participants were in the fear control process versus 68.7% in danger control about HIV/AIDS																											previous history of accompanying drug addicted friends versus 8.6% among the high self-control students (p-value =.032)*. Previous history of alcohol consumption among students with low and high self-control was 33.3% and 13.2% respectively (p-value = 0.011)*.			

Refid	Author	Title	Abstract	User	Level	Author	Date	Jurisd	PubType	PubType_comment	Quant-CaseContr	Quant-CrossSect	Quant-Other	Quant-Other_comment	Qual-FocGroup	Qual-SemiStr	Mixed	Aim	Dates	InclExclCriteria	RecruitmentMethods	DataCollection	Participants	Participants_Youth	Age_mean	Gender_Percentage	Pop_Largest	SampleOther	Outcome	Comparator	RiskFactors	Intervention	MainFindings	Notes
			and also the presence of multi-sex partners and amphetamine consumption amongst the participants. Low self-control trait and low perceived susceptibility significantly were related to having a history of multi-sex partners while high level of self-efficacy significantly increased the probability of condom use. CONCLUSION: Findings of the study were indicative of the protective role of high level of self-control, perceived susceptibility and self-efficacy factors on youth's high-risk behaviors and their preventative skills as well.																																
510	A. Salehi, M. Naghshvarian, M. Marzban, K. Bagheri	Prevalence of HIV, HCV, and High-Risk Behaviors for	OBJECTIVES: Drop In Centers (DICs) are organizations in	mohammad.alakel	5	Salehi	2015	Shiraz, Iran	PubPeer				Quant-Other	Retrospective Record Review				To estimate the prevalence of human immuno deficiency virus	04/2006 to 04/2011	-Exclusion criteria: 17 from the Voluntary Counseling and	N/A. Data extracted from patient records.	-To collect relevant information about their age,	N= 1,327 people	62.2% younger than 35 years (n=825)	-The mean age of the males was 33.75 years and that of the	79.5% Male	62.3% sex workers 61% IDU	56.6% unemployed 30.3% Homeless	Outcomes included risk factors for HIV: shared injection,	-Compared soci odemographic factors associated with	-A history of unsafe sex in men and women was	N/A	-The findings of this study indicated that the majority of	

Refid	Author	Title	Abstract	User	Level	Author	Date	Jurisd	PubType	PubType_comment	Quant-CaseContr	Quant-CrossSect	Quant-Other	Quant-Other_comment	Qual-FocGroup	Qual-SemiStr	Mixed	Aim	Dates	InclExclCriteria	RecruitmentMethods	DataCollection	Participants	Participants_Youth	Age_mean	Gender_Percentage	Pop_Largest	SampleOther	Outcome	Comparator	RiskFactors	Intervention	MainFindings	Notes
	Lankarani	Substance Users in Drop in Centers in Southern Iran	which health and social services are delivered to drug users on the basis of harm-reduction strategies. The aim of this epidemiological study was to estimate the prevalence of human immuno deficiency virus (HIV) and hepatitis C virus (HCV) infection at these centers over a 6-year period. METHODS: All of the DICs (7 in total) visited were located in Shiraz, the capital city of Fars province, southern Iran. Every patient record in these centers was studied between April 2006 and April 2011. Complementary information on the frequency of HIV was collected from the Voluntary Counseling and Testing services. A															(HIV) and hepatitis C virus (HCV) at these centers over 6 years.		Testing (VCT), were eliminated because of duplication in each of the 2 data sources. Also, 12 participants were excluded because they resided outside Shiraz<br>-Inclusion: Every patient in the 7 drop-in centers in Shiraz.		sex, level of education, imprisonment records, marital status, etc, complementary information on the frequency of HIV among this group of people was collected from the Voluntary Counseling and Testing (VCT) centers of Shiraz University of Medical Sciences.<br>-Data also extracted from patient records form drop-in centers.			females was 32.20 years			57% Single	history of imprisonment unsafe sex, housing, education, HIV & HCV prevalence, types and methods of substance use.	the risk of HIV and HCV infections.	present in 15.0% and 14.5% subjects, respectively<br>-93.3% of the subjects had used drugs when they were registered in Drop In Centers (DIC)<br>-The mean age of the first substance use was 20.24 ± 6.11 years (range: 7-59 years). The mean age of the first injection among the IDUs was 26.5 ± 7.3 years, (range: 11-66 years)<br>-71.4% of drug users had used heroin<br>-222 (82.5%) of HIV infected participants had reported shared injections.<br>-Shared injection*, history of imprisonment*, maleness*, unsafe sex*, inadequate housing*, and low education* were risk factors for HIV infection (all statistically		drug users are middle aged<br>- Shared injection, history of imprisonment, maleness, unsafe sex, inadequate housing, and low education were risk factors for HIV infection.	

Refid	Author	Title	Abstract	User	Level	Author	Date	Jurisd	PubType	PubType_comment	Quant-CaseContr	Quant-CrossSect	Quant-Other	Quant-Other_comment	Qual-FocGroup	Qual-SemiStr	Mixed	Aim	Dates	InclExclCriteria	RecruitmentMethods	DataCollection	Participants	Participants_Youth	Age_mean	Gender_Percentage	Pop_Largest	SampleOther	Outcome	Comparator	RiskFactors	Intervention	MainFindings	Notes
			logistic regression model was applied to data analysis. The study explored risk factors associated with HIV and HCV infections, which were primarily high-risk sexual behavior. RESULTS: This study examined 1,327 people, 79.5% of whom were males. The mean age of first substance use and the first injection among intravenous drug users (IDUs) were 20.24 +/- 6.11 and 26.5 +/- 7.3 years, respectively. In total, 13.5% and 20.2% of the subjects who registered at the DICs were infected with HCV and HIV, respectively. Shared injection, history of imprisonment, maleness, unsafe sex,																												significant).			

Refid	Author	Title	Abstract	User	Level	Author	Date	Jurisd	PubType	PubType_comment	Quant-CaseContr	Quant-CrossSect	Quant-Other	Quant-Other_comment	Qual-FocGroup	Qual-SemiStr	Mixed	Aim	Dates	InclExclCriteria	RecruitmentMethods	DataCollection	Participants	Participants_Youth	Age_mean	Gender_Percentage	Pop_Largest	SampleOther	Outcome	Comparator	RiskFactors	Intervention	MainFindings	Notes
			inadequate housing, and low education were risk factors for HIV infection. Shared injection, unsafe sex, history of imprisonment, and maleness were predisposing factors for HCV infection. A history of imprisonment and substance use upon registration at the DIC were significant risk factors for female sex workers. CONCLUSIONS: Our study emphasized the implementation of preventive measures like education and needle exchange program as harm-reduction strategies. Drop in Centers are important for the management of health problems, including HIV infection and																															

Refid	Author	Title	Abstract	User	Level	Author	Date	Jurisd	PubType	PubType_comment	Quant-CaseContr	Quant-CrossSect	Quant-Other	Quant-Other_comment	Qual-FocGroup	Qual-SemiStr	Mixed	Aim	Dates	InclExclCriteria	RecruitmentMethods	DataCollection	Participants	Participants_Youth	Age_mean	Gender_Percentage	Pop_Largest	SampleOther	Outcome	Comparator	RiskFactors	Intervention	MainFindings	Notes
			social problems such as crime. Periodic epidemiological studies on DICs are necessary to monitor and modulate the services delivered by these centers.																															
540	S. G. Massad, R. Karam, R. Brown, P. Glick, M. Shaheen, S. Linnemayr, U. Khammash	Perceptions of sexual risk behavior among Palestinian youth in the West Bank: a qualitative investigation	BACKGROUND: Young people in the Middle East and North Africa (MENA) are profoundly affected by violence, high unemployment, and economic hardship. Experiences of community-level violence and personal trauma increase the likelihood that young people will engage in risky behaviors that include smoking, drug use, and unsafe sex. Little is known about the sexual behavior of young people in the region, particularly in the occupied Palestinian territory (oPt).	mohammad.alakel	5	Massad	2014	Jerusalem, Palestine	PubPeer						Qual-FocGroup	Qual-SemiStr		To gain an insight into the perceived prevalence and patterns of sexual behavior among Palestinian youth.	Not reported	Young Palestinians aged 16-24 in the West Bank.	The sample was selected using a combination of purposive and convenience sampling.	-In the individual interviews, participants were asked to discuss their own behaviors.<br>-Focus group and individual interviews conducted with semi-structured interview protocols.	N=83	N=83	Median = 20, Age range: 16-24	51% Male	100% Palestinian Youth	64% were students<br>14% were employed<br>99% single	Participating in sexual activities, type of sexual activities, locations where youth engage in sexual activities, protection used while engaging in sexual activities, reasons youth engage in sexual activities outside the frame of marriage, and perceived positive and negative consequences of engaging in sex outside marriage. The interviews also asked respondents about their own engagement in risk behaviors.	N/A	-"The proportion of youth engaging in different types of sexual activity is between 30% and 40%, and this is because we are a conservative society".<br>-Most of the study participants perceived the engagement of young adults in sexual activities out of wedlock to be prevalent, despite living in a conservative society.<br>- Vaginal intercourse outside marriage was perceived to be less common than oral and anal sex due to fear of the	N/A	-The findings suggest that sexual activities outside marriage may be more common than is currently assumed.<br>-The most reported sexual activity was non-penetrative sex: oral and anal intercourse, and virtual sex. Some young people had sexual intercourse with sex workers; they went to brothels in Israel and to brothels operating clandestinely in the West Bank, including East Jerusalem. Most respondents were of the opinion that	

Refid	Author	Title	Abstract	User	Level	Author	Date	Jurisd	PubType	PubType_comment	Quant-CaseContr	Quant-CrossSect	Quant-Other	Quant-Other_comment	Qual-FocGroup	Qual-SemiStr	Mixed	Aim	Dates	InclExclCriteria	RecruitmentMethods	DataCollection	Participants	Participants_Youth	Age_mean	Gender_Percentage	Pop_Largest	SampleOther	Outcome	Comparator	RiskFactors	Intervention	MainFindings	Notes
			Our aim in this study was to gain an insight into the perceived prevalence and patterns of sexual behavior among Palestinian youth. METHODS: The study was based on ten focus groups and 17 in-depth interviews with young people aged 16-24 years as part of the formative phase of a cross-sectional representative study of risk behaviors in the West Bank, including Jerusalem, in 2012. The sample was selected using a combination of purposive and convenience sampling. Qualitative analysis was used to code detailed notes of focus groups and interviews. RESULTS: Based on																											potential consequences; it was most common among married women, for whom there is no fear of losing virginity.<br>- Many participants were not well-informed about condoms, especially girls, youths do not use condoms when engaging in sex, especially when unplanned, or because some do not know how to use it. Youths sometimes use condoms if they go to brothels, where the use of a condom is mandatory.		young people did not usually use protection during sexual intercourse.	

Refid	Author	Title	Abstract	User	Level	Author	Date	Jurisd	PubType	PubType_comment	Quant-CaseContr	Quant-CrossSect	Quant-Other	Quant-Other_comment	Qual-FocGroup	Qual-SemiStr	Mixed	Aim	Dates	InclExclCriteria	RecruitmentMethods	DataCollection	Participants	Participants_Youth	Age_mean	Gender_Percentage	Pop_Largest	SampleOther	Outcome	Comparator	RiskFactors	Intervention	MainFindings	Notes
			participants' reports, different types of sexual activity outside marriage were not uncommon, even in conservative communities. The most reported sexual activity was non-penetrative sex: oral and anal intercourse, and virtual sex. Some young people had sexual intercourse with sex workers; they went to brothels in Israel and to brothels operating clandestinely in the West Bank, including East Jerusalem. Most respondents were of the opinion that young people did not usually use protection during sexual intercourse. Many reported that youth engage in different types of sexual activity outside marriage for																															

Refid	Author	Title	Abstract	User	Level	Author	Date	Jurisd	PubType	PubType_comment	Quant-CaseContr	Quant-CrossSect	Quant-Other	Quant-Other_comment	Qual-FocGroup	Qual-SemiStr	Mixed	Aim	Dates	InclExclCriteria	RecruitmentMethods	DataCollection	Participants	Participants_Youth	Age_mean	Gender_Percentage	Pop_Largest	SampleOther	Outcome	Comparator	RiskFactors	Intervention	MainFindings	Notes
			several reasons: to challenge the culture, financial constraints and inability to marry, basic human need, personal pleasure, suppression, to kill boredom, and to prove manhood. CONCLUSIONS: In contrast with the conservative social context of the occupied Palestinian territory (oPt), the findings suggest that sexual activities outside marriage may be more common than is currently assumed. Sexual behavior in the oPt is a concern because of the low awareness of the potential health consequences. The results draw attention to the need to incorporate sexual reproductive health into the																															

Refid	Author	Title	Abstract	User	Level	Author	Date	Jurisd	PubType	PubType_comment	Quant-CaseContr	Quant-CrossSect	Quant-Other	Quant-Other_comment	Qual-FocGroup	Qual-SemiStr	Mixed	Aim	Dates	InclExclCriteria	RecruitmentMethods	DataCollection	Participants	Participants_Youth	Age_mean	Gender_Percentage	Pop_Largest	SampleOther	Outcome	Comparator	RiskFactors	Intervention	MainFindings	Notes
			national agenda and ensure that it is included in the programs of national institutions.																															
560	L. A. Ghandour, F. Mouhanna, R. Yasmine, F. El Kak	Factors associated with alcohol and/or drug use at sexual debut among sexually active university students: cross-sectional findings from Lebanon	BACKGROUND : Sexual activity accompanied by substance use can impair youth decision-making and enhance risk-taking behaviors. Less is known, however, about the sexual values, perceptions and subsequent sexual practices of youth whose sexual debut occurs while using alcohol/drugs. METHODS: A cross-sectional anonymous online survey was conducted in April-August 2012 among undergraduate and graduate university students (aged 18 to 30) attending the 4th largest private university in	nicole.andruszkiewicz	5	Ghandour et al.,	2014	Beirut, Lebanon	PubPeer			Quant-CrossSect						To examine sexual values, perceptions and subsequent sexual practices of youth living in in Beirut whose sexual debut occurs while using alcohol/drugs.	04/2012-08/2012	Undergraduate and graduate students registered in the selected large private university during Spring 2013.	All undergraduate and graduate students registered in the selected large private university during Spring 2013.	A self-administered questionnaire used was developed in English over a 5 month period.	N=983	N=983	Range= (18-30)	53.8% Male	100% students	73% with an undergraduate degree	-first time having any sexual intercourse (oral, vaginal or anal sex) and had been drinking or using drugs<br>-students sexual behaviors: familiarity with first sexual partner, level of consent at sexual debut, number of lifetime sexual partners, among other risky practices	-Comparing sexual debut while using drugs or alcohol and youth sexual perceptions/values.	-adjusting for sociodemographics, sexually active students who agreed that 'only two people who trust each other should have sexual relations' were half as likely to have used alcohol/drugs during first-time sex<br>-sexually active students who perceived that consumption of drugs or alcohol before sex took place 'a lot' (25% of the sample) versus 'little or never' were 4.76 times as likely to use alcohol/drugs themselves at sexual debut, controlling for so cio-demographics	N/A	-Students who used alcohol/drugs at sexual debut were twice as likely to have: their first oral and vaginal sex with an unfamiliar partner controlling for sex, nationality, current relationship status, living abroad after the age of 12, and spirituality.<br>-Students who had sex the first time while using alcohol/drugs were three times as likely to report having had 11 or more subsequent sexual partners versus one or two; and almost twice as likely to ever engage in something sexual they did not want to do.<br>-Perceived peer	

Refid	Author	Title	Abstract	User	Level	Author	Date	Jurisd	PubType	PubType_comment	Quant-CaseContr	Quant-CrossSect	Quant-Other	Quant-Other_comment	Qual-FocGroup	Qual-SemiStr	Mixed	Aim	Dates	InclExclCriteria	RecruitmentMethods	DataCollection	Participants	Participants_Youth	Age_mean	Gender_Percentage	Pop_Largest	SampleOther	Outcome	Comparator	RiskFactors	Intervention	MainFindings	Notes
			Beirut. Pearson's Chi-square and regression models were run using Stata/IC 10.0. RESULTS: 940 university students had engaged in oral, anal and/or vaginal sex, of whom 10% admitted to having had consumed alcohol or taken drugs at sexual debut, a behavior that was more common in the males, less religious, non-Arabs, students living alone or who had lived abroad. Students who used alcohol/drugs at sexual debut were twice as likely to have: their first oral and vaginal sex with an unfamiliar partner [odds ratio (OR) = 2.6, 95% confidence interval (CI): (1.6, 4.2) and																														pressure to have sex by a certain age and perceived peer norms to consume alcohol/drugs before sex were also strong correlates of having sex for the first time while using alcohol and/or drugs.	

Refid	Author	Title	Abstract	User	Level	Author	Date	Jurisd	PubType	PubType_comment	Quant-CaseContr	Quant-CrossSect	Quant-Other	Quant-Other_comment	Qual-FocGroup	Qual-SemiStr	Mixed	Aim	Dates	InclExclCriteria	RecruitmentMethods	DataCollection	Participants	Participants_Youth	Age_mean	Gender_Percentage	Pop_Largest	SampleOther	Outcome	Comparator	RiskFactors	Intervention	MainFindings	Notes
			OR = 2.1 (1.2, 3.5), respectively], controlling for sex, nationality, current relationship status, living abroad after the age of 12, and spirituality. Students who had sex the first time while using alcohol/drugs were three times as likely to report having had 11 or more subsequent sexual partners versus one or two [OR = 3.0 (1.5-6.0)]; and almost twice as likely to ever engage in something sexual they did not want to do [OR = 1.7 (1.1, 2.8)]. Perceived peer pressure to have sex by a certain age [OR = 1.8 (1.1, 2.9)], and perceived peer norms to consume alcohol/drugs before sex [OR = 4.8 (2.3, 9.9)] were also strong correlates of																																

Refid	Author	Title	Abstract	User	Level	Author	Date	Jurisd	PubType	PubType_comment	Quant-CaseContr	Quant-CrossSect	Quant-Other	Quant-Other_comment	Qual-FocGroup	Qual-SemiStr	Mixed	Aim	Dates	InclExclCriteria	RecruitmentMethods	DataCollection	Participants	Participants_Youth	Age_mean	Gender_Percentage	Pop_Largest	SampleOther	Outcome	Comparator	RiskFactors	Intervention	MainFindings	Notes
			having sex for the first time while using alcohol and/or drugs. CONCLUSIONS: Findings stress the importance of sexuality education for youth, and the need to begin understanding the true interplay--beyond association--between youth sexual practices and substance use behaviors from a broader public health perspective.																															
574	A. Alkaiyat, C. Schaetti, M. Liswi, M. G. Weiss	Condom use and HIV testing among men who have sex with men in Jordan	INTRODUCTION: To identify sociocultural determinants of self-reported condom use and HIV testing and examine variables related to accessibility, motivation and obstacles among men who have sex with men (MSM) in Jordan. DESIGN: Cross-sectional study among MSM who were	mohammad.alakel	5	Alkaiyat et al	2014	Amman, Aqaba, Irbid and Zarqa, Jordan	PubPeer			Quant-CrossSect						To identify sociocultural determinants of self-reported condom use and HIV testing and examine variables related to accessibility, motivation, and obstacles among men who have sex with men (MSM) in Jordan.	2011	Not reported. Recruited MSM known to the NGO.	Respondents were recruited from four main cities of Jordan (Amman, Aqaba, Irbid and Zarqa) through a partnership with an NGO called ‘‘Friends of PLWHA"	Among MSM known to the NGO, all potentially eligible men were approached by a member of their staff. Categorical and narrative data were written down during the interviews. Interviews were also voice-recorded if respondents permitted it.	Out of 112 MSM who were contacted, 97 consented to be interviewed. N= 97	N = 73 (aged between 17-25)	N=73 (age 17-25); N= 24 (age >25) Minimum age: 17	100% Males	100% MSM	94.8% Muslim; 92.8% single; 55.7% student	Sample characteristics, HIV/AIDS-related knowledge, risk perception, access to services and sources and obstacles for condom use and HIV testing were reported as frequencies. Two dichotomous outcome variables: condom use and HIV testing.	-Compared sample characteristics to condom use and HIV testing among participants.<br>-Compared HIV/AIDS-related knowledge, risk perception and support to condom use and HIV testing among participants.	Adjusted analysis for education level revealed that acknowledging MSM as a high-risk group and seeking advice from a medical doctor were significantly positively correlated with condom use at last intercourse (p<0.05)*<br><br>-Positively associated with	N/A	-An important finding from our study is that access-related stigma was an obstacle to condom use that was exclusively reported by young and unmarried MSM. Although risk perception was high, knowledge of available treatments and ART was low. Stigma was also	

Refid	Author	Title	Abstract	User	Level	Author	Date	Jurisd	PubType	PubType_comment	Quant-CaseContr	Quant-CrossSect	Quant-Other	Quant-Other_comment	Qual-FocGroup	Qual-SemiStr	Mixed	Aim	Dates	InclExclCriteria	RecruitmentMethods	DataCollection	Participants	Participants_Youth	Age_mean	Gender_Percentage	Pop_Largest	SampleOther	Outcome	Comparator	RiskFactors	Intervention	MainFindings	Notes
			identified through services of a local non-governmental organization (NGO). METHODS: Respondents were studied with a semi-structured interview based on the Explanatory Model Interview Catalogue (EMIC) framework. The vignette-based EMIC interview considered locally relevant HIV/AIDS-related knowledge, risk perception and perceived causes, as well as awareness of services and sources of support. RESULTS: Of the 97 respondents, 27% reported that they used a condom at last intercourse; 38% had been tested at least once for HIV. Positive determinants of																									Categories of perceived causes of HIV were coded and analysed.		using a condom use were higher education level, acknowledging MSM as a high-risk group, seeking advice from a medical doctor and the perceived causes of HIV ‘‘sex with prostitutes’’ and ‘‘sex with animals.’’ Awareness of available treatment was a positive determinant of HIV testing. Blood transfusion as a perceived cause of HIV and asking advice from friends were negative determinants.<br><br>-21% of MSM reported stigma as an obstacle, mainly because they felt ashamed to ask for or buy condoms. Among those who reported stigma as an obstacle, 90% were single and		reported as a major obstacle to HIV testing in our study.<br>-Jordanian MSM seem to be aware of the risk of HIV infection and effective prevention methods, and they are willing to be tested for HIV.	

Refid	Author	Title	Abstract	User	Level	Author	Date	Jurisd	PubType	PubType_comment	Quant-CaseContr	Quant-CrossSect	Quant-Other	Quant-Other_comment	Qual-FocGroup	Qual-SemiStr	Mixed	Aim	Dates	InclExclCriteria	RecruitmentMethods	DataCollection	Participants	Participants_Youth	Age_mean	Gender_Percentage	Pop_Largest	SampleOther	Outcome	Comparator	RiskFactors	Intervention	MainFindings	Notes
			condom use were higher education level, acknowledging MSM as a high-risk group, seeking advice from a medical doctor and the perceived causes "sex with prostitutes" and "sex with animals." Awareness of available treatment was a positive determinant of HIV testing. Blood transfusion as a perceived cause and asking advice from friends were negative determinants. CONCLUSIONS: Jordanian MSM seem to be aware of the risk of HIV infection and effective prevention methods, and they are willing to be tested for HIV. Our findings addressed the importance of the sexual																												younger than 25 years.			

Refid	Author	Title	Abstract	User	Level	Author	Date	Jurisd	PubType	PubType_comment	Quant-CaseContr	Quant-CrossSect	Quant-Other	Quant-Other_comment	Qual-FocGroup	Qual-SemiStr	Mixed	Aim	Dates	InclExclCriteria	RecruitmentMethods	DataCollection	Participants	Participants_Youth	Age_mean	Gender_Percentage	Pop_Largest	SampleOther	Outcome	Comparator	RiskFactors	Intervention	MainFindings	Notes
			meaning of HIV/AIDS on the control of HIV/AIDS among MSM. More effective engagement of NGOs and MSM in the prevention and control of HIV/AIDS is needed, enlisting the support of medical doctors and community health workers. Peer education should be strategically strengthened. Political commitment is needed to mitigate social stigma.																															
599	A. Mirzazadeh, F. Emmanuel, F. Gharamah, A. H. Al-Suhaibi, H. Setayesh, W. McFarland, A. A. Haghdoost	HIV prevalence and related risk behaviors in men who have sex with men, Yemen 2011	Men who have sex with men (MSM) are at growing risk of HIV infection in many parts of the world; however, the epidemic has not been well explored among this population in most Arab countries. To estimate the prevalence of	mohammad.alakel	5	Mirzazadeh	2014	Aden and Al-Hudaydah, Yemen	PubPeer			Quant-CrossSect						To estimate the prevalence of HIV and related risk behaviors among MSM in Yemen	08/ 2011 to 12/2011	Men aged at least 18 years who acknowledged engaging in male–male sex in the 6 months before the interview; were self-identified as gay, bisexual, or heterosexual; and were living in Aden or Al-Hudaydah.	-MSM were recruited at venues previously identified (NGOs)	Collected by interviewers using a structured questionnaire	N = 261	N= 166 (younger than 25)	-Age in years (mean) 23.8<br>-95% CI = (17.8–29.8)	100% Male	100% MSM	-50% had no education beyond primary school<br>-22.6% reported selling sex as main source of income	Outcomes: sexual risk behaviors by partner type (number, insertive or receptive role, age of initial anal sexual intercourse, oral sex, condom use, reasons for nonuse, ways of finding partners, sexual contact with women);	Not applicable	-Protective factors: One-fourth (25.8%) had tested for HIV in the last year and received results; 27.8 % had comprehensive knowledge about HIV; 20.0 % reported condom use at last anal sex<br>-Facilitates risk behaviour: 31.4	N/A	-HIV is concentrated among MSM in Yemen and that the potential is high for further spread.<br>-HIV prevalence was 5.9 %.<br>-Drug use was relatively low among participants. Consistent condom use was low,	

Refid	Author	Title	Abstract	User	Level	Author	Date	Jurisd	PubType	PubType_comment	Quant-CaseContr	Quant-CrossSect	Quant-Other	Quant-Other_comment	Qual-FocGroup	Qual-SemiStr	Mixed	Aim	Dates	InclExclCriteria	RecruitmentMethods	DataCollection	Participants	Participants_Youth	Age_mean	Gender_Percentage	Pop_Largest	SampleOther	Outcome	Comparator	RiskFactors	Intervention	MainFindings	Notes
			HIV and related risk behaviors among MSM in Yemen, we recruited 261 adult MSM from the port cities of Aden and Al-Hudaydah through venue- and facility-based sampling. Behavioral data were collected with a face-to-face questionnaire, and HIV status was determined by serological testing. HIV prevalence was 5.9 % (95 % CI 4.8-7.3). One-fourth (25.8 %, 95 % CI 20.7-31.5) had tested for HIV in the last year and received results; 27.8 % (95 % CI 22.5-33.7) had comprehensive knowledge about HIV; 20.0 % (95 % CI 15.8-25.0) reported condom use at last anal sex; and 31.4 % (95 % CI 25.9-37.3) reported that																										substance use (including injection drug use, sex while using, sex with PWID, exchanging sex for drugs); knowledge and history of sexually transmitted diseases; knowledge of and attitudes about HIV/AIDS		% reported that they or their sexual partner had a sexually transmitted disease symptom. Injecting drugs in the last year was reported by 0.8 %.		ranging from 6.0 % with female partners to 10.1 % with casual partners.<br>-Nearly all MSM had heard of AIDS. However, only 27.8 % of participants had comprehensive knowledge of HIV.<br>-Most (89.6 %) MSM had heard of STDs. STD symptoms in the last 12 months were reported by 26.9 % of participants, most of whom (67.1 %) did not seek treatment.	

Refid	Author	Title	Abstract	User	Level	Author	Date	Jurisd	PubType	PubType_comment	Quant-CaseContr	Quant-CrossSect	Quant-Other	Quant-Other_comment	Qual-FocGroup	Qual-SemiStr	Mixed	Aim	Dates	InclExclCriteria	RecruitmentMethods	DataCollection	Participants	Participants_Youth	Age_mean	Gender_Percentage	Pop_Largest	SampleOther	Outcome	Comparator	RiskFactors	Intervention	MainFindings	Notes
			they or their sexual partner had a sexually transmitted disease symptom. Injecting drugs in the last year was reported by 0.8 % (95 % CI 0.1-9.2). Multiple risk behaviors, low HIV knowledge, few preventive behaviors, and HIV prevalence greater than 5 % denote a concentrated and potentially expanding HIV epidemic among MSM in Yemen. No time should be lost in intervening to prevent further expansion of the epidemic to levels already seen among MSM outside the Middle East.																															
610	L. Sajadi, A. Mirzazadeh, S. Navadeh, M. Osooli, R. Khajehkazemi, M. M. Gouya, N. Fahimfar, O. Zamani, A. A. Haghdoost	HIV prevalence and related risk behaviours among female sex workers in Iran: results of the national biobehavioural survey, 2010	OBJECTIVES: To determine the prevalence of HIV and related behavioural risks among Iranian female sex workers	mohammad.alakel	5	Sajadi et al	2013	Iran	PubPeer			Quant-CrossSect						To determine the prevalence of HIV and related behavioural risks among Iranian female sex workers via the first national	04/2010 to 07/2010	Female sex workers age ?18 years, selling sex for money, drugs, or goods in the last 12 months, and a history of practising sex	Facility-based sampling from 21 sites in 14 cities in Iran. The recruiting sites were a combination of NGOs, STD clinics, and drop-	We collected dried blood samples and conducted face-to-face interviews using a standardized questionnaire.	N = 872	N = 210 (aged 18 to 24)	-The mean age of the respondents was 31.8 years (SD 9.1, median 30 years).<br>-Minimum age: 18<br>-N = 210	100% Female	100% Female Sex workers	-63.5% of female sex workers don't have another source of income<br>-73.8% of Female sex workers ever	History of sex work, and related sex and drug use risks was based on previous questionnaires. Additional indicators	Compared HIV prevalence by several indicators of risk behaviour.	The prevalence of HIV infection was significantly associated with having a history of drug injection and having another source of income in	N/A	-The prevalence of HIV was considerable among FSW particularly those who had a history of drug injection<br>- Female sex	

Refid	Author	Title	Abstract	User	Level	Author	Date	Jurisd	PubType	PubType_comment	Quant-CaseContr	Quant-CrossSect	Quant-Other	Quant-Other_comment	Qual-FocGroup	Qual-SemiStr	Mixed	Aim	Dates	InclExclCriteria	RecruitmentMethods	DataCollection	Participants	Participants_Youth	Age_mean	Gender_Percentage	Pop_Largest	SampleOther	Outcome	Comparator	RiskFactors	Intervention	MainFindings	Notes
			(FSW) via the first national biobehavioural surveillance survey. METHODS: In 2010, 1005 FSW were approached and 872 recruited using facility-based sampling from 21 sites in 14 cities in Iran. We collected dried blood samples and conducted face-to-face interviews using a standardised questionnaire. Data were weighted based on the response rate and adjusted for the clustering effect of the sampling site. Adjustment was performed by weighting based on the sampling fraction of each site using a prior estimate of its total size of the FSW population. RESULTS: The prevalence of HIV infection (95% CI) was															biobehavioural surveillance survey.		work for at least 6 months	in centres				(23.8%) for age range between 18-24<br>-N = 362 (41.7%) for age range between 25-34<br>-N = 292 (34.5%) for age 35=>			used drugs<br>-79.5% of female sex workers answered "No" to ever injected drugs (among those who had a history of any drug use)<br>-49% single	included were engagement in temporary marriage (Sighe), local venues that FSW use for either finding clients or having sex, history of being in prison and knowledge of available sites for HIV testing. Also asked about STIs and treatment seeking patterns.		addition to sex work. Female sex workers who injected drugs were more than three times as likely to be infected as those who did not inject drugs.		workers who injected drugs were more than three times as likely to be infected as those who did not inject drugs; this suggests that a considerable fraction of HIV infections among these female sex workers were acquired through drug injection.<br>-Female sex worker who had another source of income beyond selling sex were more likely to be HIV positive.<br>-We found 30 HIV-positive samples. The overall prevalence of HIV infection was 4.5%. Any drug use was reported by 73.8% of participants, and among this subgroup, 20.5% had a history of injection drug use.	

Refid	Author	Title	Abstract	User	Level	Author	Date	Jurisd	PubType	PubType_comment	Quant-CaseContr	Quant-CrossSect	Quant-Other	Quant-Other_comment	Qual-FocGroup	Qual-SemiStr	Mixed	Aim	Dates	InclExclCriteria	RecruitmentMethods	DataCollection	Participants	Participants_Youth	Age_mean	Gender_Percentage	Pop_Largest	SampleOther	Outcome	Comparator	RiskFactors	Intervention	MainFindings	Notes
			4.5% (2.4 to 8.3) overall, 4.8% (2.2 to 9.8) among those who had reported a history of drug use and 11.2% (5.4 to 21.5) among those who had a history of injection drug use. The frequencies of condom use in the last sexual act with paying clients and non-paying partners were 57.1% and 36.3%, respectively. Any drug use was reported by 73.8% of participants, and among this subgroup, 20.5% had a history of injection drug use. CONCLUSIONS: The prevalence of HIV was considerable among FSW particularly those who had a history of drug injection. A combination of																															

Refid	Author	Title	Abstract	User	Level	Author	Date	Jurisd	PubType	PubType_comment	Quant-CaseContr	Quant-CrossSect	Quant-Other	Quant-Other_comment	Qual-FocGroup	Qual-SemiStr	Mixed	Aim	Dates	InclExclCriteria	RecruitmentMethods	DataCollection	Participants	Participants_Youth	Age_mean	Gender_Percentage	Pop_Largest	SampleOther	Outcome	Comparator	RiskFactors	Intervention	MainFindings	Notes
			prevention efforts addressing unsafe sex and injection are needed to prevent further transmission of HIV infection.																															
616	R. Khajehkazemi, M. Osooli, L. Sajadi, M. Karamouzian, A. Sedaghat, N. Fahimfar, A. Safaie, E. Mostafavi, A. A. Haghdoost	HIV prevalence and risk behaviours among people who inject drugs in Iran: the 2010 National Surveillance Survey	OBJECTIVES: To assess the prevalence of HIV and related risk behaviours among people who inject drugs (PWID) in Iran. METHODS: We conducted a national cross-sectional bio-behavioural surveillance survey between March and July 2010, interviewing male PWID from a geographically dispersed sample through a facility-based sampling method. RESULTS: We recruited 2480, and tested 2290 PWID. The overall prevalence of HIV was 15.2% (95% CI 9.7% to 23.1%). Among	mohammad.alakel	5	Khajehkazemi et al	2013	Iran	PubPeer			Quant-CrossSect						To assess the prevalence of HIV and related risk behaviours among people who inject drugs in Iran.	03/ 2010 to 07/2010	Men ?18 years of age, who had injected drugs at least once during the past 12 months.	Recruited by convenience sampling at the center regardless of their known or unknown HIV status and with verbal consent	A structured questionnaire was used to collect data. Provincial supervisors were trained to recruit, interview, and collect dried blood samples (DBS) based on a standard protocol.	A total of 2518 PWID were asked to participate in this study.<br>27 individuals did not meet the eligibility criteria, 11 refused to provide informed consent<br>Out of 2480 PWID, 2417 provided informed consent for HIV testing<br>Unable to complete the HIV test on 127 dried blood samples.<br>(N=2290 tested)	N= approx. 318	The mean (SD) age at the time of the study = 34.6 (8.9)<br>- N= 13.9% for age range between 18-25<br>- N= 46.6% for age ranged 26-35<br>- N= 39.5 for age 36=>	100% Male	100% IUD	47.3% single; 65.2% have high school education; 87.75% have a temp job or are unemployed	Data collected on key indicators of injection and sexual risk behaviours: age at first drug use, having at least one injection in the last month, sharing injection tools with others in the last month , condom use at last sexual intercourse.	HIV prevalence compared across sociodemographic characteristics and risk behaviours	-The prevalence of HIV was significantly positively associated with age.<br>-Having above high school education, and permanent job were protective factors.	N/A	-PWID are still the most affected high-risk group by HIV in Iran, with a prevalence of about 15%.<br>-Unsafe injection and sex are still common among Iranian people who inject drugs<br>-Among subgroups, unemployed PWID had the highest prevalence of HIV (20.1%), and those with above high school education had the lowest (4.6%). Among those who had injected drugs over the last month, 36.9% had used a non-sterile needle. Over the past 12 months	

Refid	Author	Title	Abstract	User	Level	Author	Date	Jurisd	PubType	PubType_comment	Quant-CaseContr	Quant-CrossSect	Quant-Other	Quant-Other_comment	Qual-FocGroup	Qual-SemiStr	Mixed	Aim	Dates	InclExclCriteria	RecruitmentMethods	DataCollection	Participants	Participants_Youth	Age_mean	Gender_Percentage	Pop_Largest	SampleOther	Outcome	Comparator	RiskFactors	Intervention	MainFindings	Notes
			those who had injected drugs over the last month, 36.9% had used a non-sterile needle, and 12.6% had practiced shared injection. Over the past 12 months preceding the interview, 30.4% had sold sex for money, drugs, goods or a favour. In the multivariate analysis, the prevalence of HIV had a positive association with age, while having above high school education, and permanent job were protective. CONCLUSIONS: Unsafe injection, and sexual risk behaviours are still frequent and the prevalence of HIV among PWID remains high. Intensified efforts are needed to prevent the further spread of HIV among																														preceding the interview, 30.4% had sold sex for money, drugs, goods or a favour.	

Refid	Author	Title	Abstract	User	Level	Author	Date	Jurisd	PubType	PubType_comment	Quant-CaseContr	Quant-CrossSect	Quant-Other	Quant-Other_comment	Qual-FocGroup	Qual-SemiStr	Mixed	Aim	Dates	InclExclCriteria	RecruitmentMethods	DataCollection	Participants	Participants_Youth	Age_mean	Gender_Percentage	Pop_Largest	SampleOther	Outcome	Comparator	RiskFactors	Intervention	MainFindings	Notes
			Iranian PWID and their sexual partners.																															
627	J. J. Valadez, S. Berendes, C. Jeffery, J. Thomson, H. Ben Othman, L. Danon, A. A. Turki, R. Saffialden, L. Mirzoyan	Filling the Knowledge Gap: Measuring HIV Prevalence and Risk Factors among Men Who Have Sex with Men and Female Sex Workers in Tripoli, Libya	BACKGROUND: Publications on Libya's HIV epidemic mostly examined the victims of the tragic nosocomial HIV outbreak in the 1990s and the related dispute about the detention of foreign medical workers. The dispute resolution in 2003 included an agreement with the European Union on humanitarian cooperation and the development of Libya's first National HIV Strategy. As part of this we conducted Libya's first bio-behavioural survey among men having sex with men (MSM) and female sex workers (FSW). METHODS: Using responde nt-driven	mohammad.alakel	5	Valadez	2013	Tripoli, Libya	PubPeer			Quant-CrossSect						To estimate the prevalence of HIV, hepatitis B virus (HBV), hepatitis C virus (HCV), and related risk factors among MSM and FSW in Tripoli, Libya	09/2010-02/2011	MSM were eligible to join the study if they were born male, and had anal sex with another man in the last six months.<br>FSW were eligible if they were born female, and had earned all or part of their income through the exchange of money for vaginal or anal sex with more than one client in the last six months. In addition, participants of both groups had to be 15 years old	the peer-driven chain-referral method was used to recruit participants	Eligible participants underwent a face-to-face interview. The interviews were guided by a structured questionnaire.	We recruited a total of 227 eligible MSM and 69 FSW.<br>N total = 296	N of MSM between age 15-29 = 204<br>N of FSW between age 15-29 = 37<br>N total = 241	N/A	77% male	MSM and FSW	52.1 % of MSM and 66.4% of FSW have no source of income<br>45.1% of MSM and 81,3% of FSW have less than high education level completed	questions on socio-demographics, sexual and other risk behaviours, HIV-related knowledge, access to services for HIV prevention and care, and other HIV-related factors.<br>-Having had .3 anal sex partners during the past 6 months<br>-Having had group sex at some point in the past<br>-Having had used a condom during last anal intercourse<br>-Ever having used lubricants<br>-MSM having had anal intercourse with a commercial partner in the past six months<br>-Having had used a condom at last commercial intercourse	comparing Hepatitic C infection, HIV/ Hepatitis B co-infection, and HIV/ Hepatitis C co-infection among MSM and FSW	-For MSM the median age of first anal intercourse with a man was 17 years.<br>-An estimated 58.1% of MSM had their first sexual intercourse with males before reaching 18 years, and 13.8% had a forced sexual debut<br>-44.2% of respondents reported having had >3 anal sex partners during the past 6 months, and 16.9% had group sex at some point in the past.<br>-Only 21% of MSM used a condom during the last anal intercourse. More than half of the respondents (54.3%) reported ever having used lubricants, of	N/A	Sexual risk factors among MSM included a high number of anal sex partners and low use of condoms and the recommended waterbased lubricants. In addition, a substantial proportion of MSM had group sex and commercial sex. Sexual risk factors among FSW included a high number of sex partners, and a condom use rate that was higher than among MSM, but still insufficient to ensure consistent protection.<br>The study did not show any cases of HIV/HBV co-infection but revealed an HIV/HCV coinfection of	

Refid	Author	Title	Abstract	User	Level	Author	Date	Jurisd	PubType	PubType_comment	Quant-CaseContr	Quant-CrossSect	Quant-Other	Quant-Other_comment	Qual-FocGroup	Qual-SemiStr	Mixed	Aim	Dates	InclExclCriteria	RecruitmentMethods	DataCollection	Participants	Participants_Youth	Age_mean	Gender_Percentage	Pop_Largest	SampleOther	Outcome	Comparator	RiskFactors	Intervention	MainFindings	Notes
			sampling, we conducted a cross-sectional study to estimate the prevalence of HIV, hepatitis B virus (HBV), hepatitis C virus (HCV), and related risk factors among 227 MSM and 69 FSW in Tripoli (FSW recruitment ended prematurely due to the political events in 2011). RESULTS: For MSM we estimated an HIV prevalence of 3.1%, HBV prevalence of 2.9%, and HCV prevalence of 7.3%, and for FSW an HIV prevalence of 15.7%, HBV prevalence of 0%, and HCV prevalence of 5.2%. We detected high levels of risk behaviours, poor HIV-related knowledge, high stigma and lack of prevention programmes.																										-MSM having had sexual intercourse with a female partner in the past<br>-MSM having had intercourse with a woman in the last six months<br>-FSW having had been forced to have sex during the past year<br>-FSW having had 10–50 sex partners during the past six months<br>-FSW having had consistent condom use during sex with one-time clients		which only 0.6% were the recommended water-based lubricants.<br>-26.5% of MSM had anal intercourse with a commercial partner in the past six months, but only 19.4% of these used a condom at last commercial intercourse.<br>-More than two thirds (68.5%) of MSM also had sexual intercourse with a female partner in the past, and more than half (53%) had intercourse with a woman in the last six months.<br>-The median reported age of first intercourse with a woman was 18 years.<br>-38.5% of MSM reported risky sex with both men and women during the past six months. Almost one third (29.5%) of respondents had used non-		2.1% for MSM and 3.7% for FSW.	

Refid	Author	Title	Abstract	User	Level	Author	Date	Jurisd	PubType	PubType_comment	Quant-CaseContr	Quant-CrossSect	Quant-Other	Quant-Other_comment	Qual-FocGroup	Qual-SemiStr	Mixed	Aim	Dates	InclExclCriteria	RecruitmentMethods	DataCollection	Participants	Participants_Youth	Age_mean	Gender_Percentage	Pop_Largest	SampleOther	Outcome	Comparator	RiskFactors	Intervention	MainFindings	Notes
			These results must be interpreted in the context of the political situation which prohibited reaching an ideal sample size for FSW. CONCLUSION: There is urgent need to implement an effective National HIV Strategy informed by the results of this research. The risk of transmission within different risk groups and to the general population may be high given the recent military events that led to increased violence, migration, and the disruption of essential HIV-related services.																												injecting drugs in the past six months, such as Hashish or Tranquilizers, and 4% reported having ever injected drugs.<br>-Only 5.7% of MSM felt they were at high risk for HIV infection.<br>-18.2% of FSW reported having been forced to have sex during the past year.<br>-42.8% of FSW had 10–50 sex partners during the past six months and 30.5% had 50 or more.<br>-63.4% of FSW reported consistent condom use during sex with one-time clients, and 56.8% during sex with regular clients.<br>27.8% of FSW experienced STI symptoms in the past year.			
665	A. Mirzazadeh, A. A. Haghdoost, S. Nedjat, S.	Accuracy of HIV-related risk behaviors reported by	We quantified discrepancies in reported behaviors of	mohammad.alakel	5	Mirzazadeh	2013	Tehran, Iran	PubPeer							Qual-SemiStr		To provide a quantified gauge of reporting biases	05/ 2011 to 10/ 2011	Female sex workers aged 18–65 years and selling sex	Recruited a convenience sample of FSWs from non-	In-depth interview responses were recorded by	N = 63	23.8% of female sex workers were under 25 (n=15)	A total of 63 FSW age 18–44 years, mean 28.5 (27.0–29.9)	100% females	100% female sex workers	-96.8% ever used drugs<br>-81% were ever married	- Arrested and incarcerated in the last 12 months, ever	Comparing outcomes between the face to face	On average, in the face to face interviews the women reported	N/A		FSW significantly under-reported number of	

Refid	Author	Title	Abstract	User	Level	Author	Date	Jurisd	PubType	PubType_comment	Quant-CaseContr	Quant-CrossSect	Quant-Other	Quant-Other_comment	Qual-FocGroup	Qual-SemiStr	Mixed	Aim	Dates	InclExclCriteria	RecruitmentMethods	DataCollection	Participants	Participants_Youth	Age_mean	Gender_Percentage	Pop_Largest	SampleOther	Outcome	Comparator	RiskFactors	Intervention	MainFindings	Notes
	Navadeh, W. McFarland, K. Mohammad	female sex workers, Iran: a method to quantify measurement bias in marginalized populations	female sex workers (FSW) by comparing 63 face-to-face interviews (FTFI) to in-depth interviews (IDI), with corroboration of the directions and magnitudes of reporting by a panel of psychologists who work with FSW. Sensitivities, specificities, positive and negative predictive values (PPV and NPV) were assessed for FTFI responses using IDI as a "gold standard". Sensitivities were lowest in reporting symptoms of sexually transmitted infections (63.9 %), finding sex partners in venues (52.4 %) and not receiving HIV test results (66.7 %). Specificities (all >83 %) and PPVs (all >74.0															in female sex worker's behaviors to understand true HIV risk in marginalized populations in Iran.		in the last 6 months were eligible.	governmental organizations and public health facilities serving sex workers in two cities	short notes and later transferred and aligned to the face to face interview questionnaire			(95 % CI)			-49.2% had high school education<br>-49.2% arrested in last 12 months	use of drugs, history of genital ulcer or discharge in the last 12 months, non-condom use at last sex act with a client and being associated with a venue (e.g., home or shelter) where persons find commercial sex.<br>-Sexual contacts, last 7 days<br>-Non-condom use acts, last 7 days<br>-Age at first sex for money, drugs or shelter<br>-Clients, last 7 days<br>-Days in last week with sex with client<br>-Monthly income through commercial sex<br>-Income through last commercial sex act	interviews vs. the in-depth interviews	3.4 sexual contacts with their clients in last 7 days, with a mean of 1.9 times without condom use. The first sex act for money, drugs or shelter was at the age of 21 years. In the last 7 days, FSW reported an average of just over 3 clients over an average of approximately 3 days. They earned $277.80 USD in a month by selling sex, with the last act averaging $21.90<br>-Comparing the face to face interviews to the in depth interviews, the FSW reported 1.5 fewer sexual contacts (P<0.001), 0.4 fewer non-condom use sexual acts (P = 0.04), 0.8 fewer clients (P = 0.01), and 0.9 fewer days exchanging sex (P = 0.01) in the		clients, sexual contacts and non-condom use sex acts with clients and number of days engaging in sex work in the preceding week..	

Refid	Author	Title	Abstract	User	Level	Author	Date	Jurisd	PubType	PubType_comment	Quant-CaseContr	Quant-CrossSect	Quant-Other	Quant-Other_comment	Qual-FocGroup	Qual-SemiStr	Mixed	Aim	Dates	InclExclCriteria	RecruitmentMethods	DataCollection	Participants	Participants_Youth	Age_mean	Gender_Percentage	Pop_Largest	SampleOther	Outcome	Comparator	RiskFactors	Intervention	MainFindings	Notes
			%) were higher than NPV. FSW significantly under-reported number of clients, sexual contacts and non-condom use sex acts with clients and number of days engaging in sex work in the preceding week. This study provides a quantified gauge of reporting biases in FSW behaviors. Such estimates and methods help better understand true HIV risk in marginalized populations and calibrate survey estimates accordingly.																												last week.			
684	G. J. Wagner, F. M. Aunon, R. L. Kaplan, Y. Rana, D. Khouri, J. Tohme, J. Mokhbat	A qualitative exploration of sexual risk and HIV testing behaviors among men who have sex with men in Beirut, Lebanon	Men who have sex with men (MSM) may account for most new HIV infections in Lebanon, yet little is known about the factors that influence sexual risk behavior	mohammad.alakel	5	Wagner et al.,	2012	Beirut, Lebanon	PubPeer								Mixed	To explore psychosocial processes that influence sexual risk behavior and HIV testing among men who have sex with men	Fall 2011 (Month not specified)	Inclusion criteria: male, age 18 years or older, and reported engaging in any sexual activity with another male in the past year.	Participants were referred by members of our community advisory board and collaborating non-governmental organizations that provide services to sexual	-Using a semi-structured interview guide, interviews were digitally recorded and conducted in the language preferred by the participant (Arabic, French or English)	N= 31	-N= 16 were aged 18–25 years<br>-N= 15 above age 25	Mean age was 28.4 years (SD = 10.5; range: 18–65 years)	100% Male	77% gay and 23% bisexual MSM	87% had at least some college education, and most participants were either employed (55%) or attending university (35%), religious affiliation (71%)	the interview covered the following topic areas: 1) comfort with and disclosure of sexual orientation, 2) experiences of support and/or stigma in response to	N/A	-With regard to anal sex, 15 reported inconsistent condom use, while 11 reported always using condoms.<br>-7 men (participants) had never been tested for HIV	N/A	- Half reported not using condoms consistently and one quarter had not been HIV-tested.<br>-Respondents who were very comfortable with their sexual orientation and	

Refid	Author	Title	Abstract	User	Level	Author	Date	Jurisd	PubType	PubType_comment	Quant-CaseContr	Quant-CrossSect	Quant-Other	Quant-Other_comment	Qual-FocGroup	Qual-SemiStr	Mixed	Aim	Dates	InclExclCriteria	RecruitmentMethods	DataCollection	Participants	Participants_Youth	Age_mean	Gender_Percentage	Pop_Largest	SampleOther	Outcome	Comparator	RiskFactors	Intervention	MainFindings	Notes
			and HIV testing in this population. Qualitative interviews were conducted with 31 MSM living in Beirut, and content analysis was used to identify emergent themes. Mean age of the participants was 28.4 years, and all identified as either gay (77%) or bisexual (23%). Half reported not using condoms consistently and one quarter had not been HIV-tested. Many described not using condoms with a regular partner in the context of a meaningful relationship, mutual HIV testing, and a desire to not use condoms, suggesting that trust, commitment and intimacy play a role in condom use decisions.																		minorities; some participants also referred their friends.								disclosure, 3) sexual behavior including condom use, 4) HIV testing, and 5) discussion of HIV status, risk and condom use with sex partners.		-Many of the men (10 of 24) who had been tested for HIV stated they were very comfortable with their sexual orientation compared to just one of the 7 untested men.<br>-Two thirds (n = 16) of the 24 men who had been tested for HIV were using condoms consistently compared to none of the 7 men who had never been tested<br>- Over half of the sample (n = 17) reported that they did not use condoms during sex with partners with whom they were either in a relationship or who were regular sex partners<br>- Two-thirds (n = 9) of the 13 participants who were both uncomfortable with their sexual orientation and		who had disclosed their sexuality to family and parents tended to be more likely to use condoms consistently and be tested for HIV.<br>- Condoms were more likely to be used with casual partners, partners believed to be HIV positive and with partners met online. Fear of infection motivated many to get HIV tested and use condoms, but also led some to avoid HIV testing in fear of disease and social stigma if found to be infected.	

Refid	Author	Title	Abstract	User	Level	Author	Date	Jurisd	PubType	PubType_comment	Quant-CaseContr	Quant-CrossSect	Quant-Other	Quant-Other_comment	Qual-FocGroup	Qual-SemiStr	Mixed	Aim	Dates	InclExclCriteria	RecruitmentMethods	DataCollection	Participants	Participants_Youth	Age_mean	Gender_Percentage	Pop_Largest	SampleOther	Outcome	Comparator	RiskFactors	Intervention	MainFindings	Notes
			Condoms were more likely to be used with casual partners, partners believed to be HIV-positive, and with partners met online where men found it easier to candidly discuss HIV risk. Fear of infection motivated many to get HIV tested and use condoms, but such affect also led some to avoid HIV testing in fear of disease and social stigma if found to be infected. Respondents who were very comfortable with their sexual orientation and who had disclosed their sexuality to family and parents tended to be more likely to use condoms consistently and be tested for HIV. These findings indicate that similar																												had not disclosed to either parent reported inconsistent condom use compared to just one-third (n = 6) of the other 18 men in the sample.			

Refid	Author	Title	Abstract	User	Level	Author	Date	Jurisd	PubType	PubType_comment	Quant-CaseContr	Quant-CrossSect	Quant-Other	Quant-Other_comment	Qual-FocGroup	Qual-SemiStr	Mixed	Aim	Dates	InclExclCriteria	RecruitmentMethods	DataCollection	Participants	Participants_Youth	Age_mean	Gender_Percentage	Pop_Largest	SampleOther	Outcome	Comparator	RiskFactors	Intervention	MainFindings	Notes
			factors influence the condom use and HIV testing of MSM in Beirut as those observed in studies elsewhere of MSM; hence, prevention efforts in Lebanon can likely benefit from lessons learned and interventions developed in other regions, particularly for younger, gay-identified men. Further research is needed to determine how prevention efforts may need to be tailored to address the needs of men who are less integrated into or do not identify with the gay community.																															
688	A. S. Ismael, J. M. Sabir Zangana	Knowledge, attitudes and practice of condom use among males aged (15-49) years in Erbil	BACKGROUND AND OBJECTIVES: Globally, condom is an important method of family	mohammad.alakel	5	Ismael	2012	Erbil, Iraq	PubPeer			Quant-CrossSect						To assess knowledge, attitudes and practices in addition to socio-demographic factors of	03/ 2010 to 03/ 2011	The target population was adult males aged 15-49 years which are chosen from different	Participants were recruitedfrom 15 randomly selected primary health care centers (PHCs)	Closed-ended self-administered questionnaire was used	N= 600	N= 333 (40 + 126 + 167)	Range 15-49	100% Males	99 % of single men report non condom use	-90% of participants live in Urban areas<br>-64% married<br>-69.5% non-employed<br>-11.8% were	The respondents were distributed according to their age, district location, place of residence,	N/A	-The rate of condom use among 600 respondents was 12%<br>-88% never used condoms	N/A	The study showed that the rate of condom use was low among the studied sample (only 12%). This	

Refid	Author	Title	Abstract	User	Level	Author	Date	Jurisd	PubType	PubType_comment	Quant-CaseContr	Quant-CrossSect	Quant-Other	Quant-Other_comment	Qual-FocGroup	Qual-SemiStr	Mixed	Aim	Dates	InclExclCriteria	RecruitmentMethods	DataCollection	Participants	Participants_Youth	Age_mean	Gender_Percentage	Pop_Largest	SampleOther	Outcome	Comparator	RiskFactors	Intervention	MainFindings	Notes
		Governorate	planning and prevention of sexually transmitted infections especially human immune deficiency virus HIV/ acquired immune deficiency syndrome AIDS. Family planning saves lives of women and children and improves the quality of life. This study was conducted to assess knowledge, attitudes and practices in addition to socio-demographic factors of condom use among males in Erbil governorate. SUBJECTS AND METHOD: A cross sectional study conducted on randomly selected sample of 600 males aged 15-49 years from 3 districts of Erbil governorate of Iraqi Kurdistan															condom use among males in Erbil governorate		educational and occupational groups	which belongs to three randomly selected districts of Erbil governorate of Iraqi Kurdistan region							illiterate	marital status, occupation and educational level of the respondents and their wives, number of living children, crowding index, possession of car and type of housing		in their life<br>-The main reason for condom use among 12% of condom users was for family planning 91.7% and 2.8% used a condom for prevention of STIs<br>-Refuse condom use by 44.5% of respondents<br>-the highest level of condom use was among respondents of age group of 35-39 years, which was significantly higher than another age group (x2 =16.643, P = 0.005)*<br>-Among 12% condom users, 13% were living in urban, 3.3% were living in rural areas. The place of residence of respondents has a statistically significant association with condom use (x2 = 4.742, p= 0.016)*		was due to lack of knowledge about the proper and effective use of condoms, low perception of risk of HIV and other STIs, misperception about harmful effects of condoms and the use of other family planning methods by respondents and their female sexual partner	

Refid	Author	Title	Abstract	User	Level	Author	Date	Jurisd	PubType	PubType_comment	Quant-CaseContr	Quant-CrossSect	Quant-Other	Quant-Other_comment	Qual-FocGroup	Qual-SemiStr	Mixed	Aim	Dates	InclExclCriteria	RecruitmentMethods	DataCollection	Participants	Participants_Youth	Age_mean	Gender_Percentage	Pop_Largest	SampleOther	Outcome	Comparator	RiskFactors	Intervention	MainFindings	Notes
			region by using multistage cluster sampling method to assess their knowledge, attitudes and practice of condom use. RESULTS: Only 12% of respondents had ever used condoms. The main reason for condom use was for family planning in about 91.7%. About a quarter of respondents reported knowing how to use condom correctly. Condoms were considered by respondents as an effective method of contraception and prevention of sexually transmitted infections 33.2% and 28.3% respectively. While 30.3% of them believed that condom use had some harmful effects. The main reason to non																												-Among (12%) condom users, the lowest percentage of condom use were among illiterate, while the highest percentage of condom were among respondents with diploma, university and high education (x2 = 26.440, p<0.001)*			

Refid	Author	Title	Abstract	User	Level	Author	Date	Jurisd	PubType	PubType_comment	Quant-CaseContr	Quant-CrossSect	Quant-Other	Quant-Other_comment	Qual-FocGroup	Qual-SemiStr	Mixed	Aim	Dates	InclExclCriteria	RecruitmentMethods	DataCollection	Participants	Participants_Youth	Age_mean	Gender_Percentage	Pop_Largest	SampleOther	Outcome	Comparator	RiskFactors	Intervention	MainFindings	Notes
			condom use was lack of need in 45.5%, fertility related reasons in 17% and the use of other methods by the female partner 13.6%. Although 64% of respondents heard about AIDS /HIV and 71.7% about STIS in general, only few felt that they are at risk of STIs 9.5% and HIV infection 8.5%. CONCLUSION: The study showed that the rate of condom use was low among the studied sample. This was due to lack of knowledge about proper and effective use of condoms, low perception of risk of HIV and other STIs, misperception about harmful effects of condoms and the use of other family planning methods by respondents																																

Refid	Author	Title	Abstract	User	Level	Author	Date	Jurisd	PubType	PubType_comment	Quant-CaseContr	Quant-CrossSect	Quant-Other	Quant-Other_comment	Qual-FocGroup	Qual-SemiStr	Mixed	Aim	Dates	InclExclCriteria	RecruitmentMethods	DataCollection	Participants	Participants_Youth	Age_mean	Gender_Percentage	Pop_Largest	SampleOther	Outcome	Comparator	RiskFactors	Intervention	MainFindings	Notes
			and their female sexual partner.																															
793	B. Al-Iryani, K. Al-Sakkaf, H. Basaleem, G. Kok, B. van den Borne	Process evaluation of a three-year com munity-based peer education intervention for HIV prevention among Yemeni young people	This community process evaluation highlights key enabling factors that facilitated the implementation of a community peer education program for youth HIV prevention in four poor and vulnerable areas of Aden, Yemen. It also explains the implementation process and provides a deeper understanding of the impact evaluation outcomes, which revealed improved HIV knowledge and risk perception and decreased stigma and risky behavior. This process evaluation was based on qualitative methodologies, where five focus group discussions and	mohammad.ala kel	5	Al-Iryani	2010	Aden, Yemen	PubPeer						Qual-FocGroup	Qual-SemiStr		To evaluate and highlight key enabling factors of a community peer education program for youth HIV prevention in four poor and vulnerable areas of Aden, Yemen.	02/ 2009 Control survey- 2005 Intervention 2005-2008 Survey of students targeted and not targeted- 2008 Focus groups and interviews- 02/2009	Community peer educators, community focal points, targeted young people, and local councils.	Not reported	-In-depth interviews and focus group discussions were facilitated by nine data collectors and were recorded, transcribed and coded<br>-Some quantitative results were used from the q uasi-experimental evaluation, which included a random sample of 700 young people from the four co mmunities.(see ref ID 776)	N= 52	Not reported. “Community focal points, although the majority were young people, were also older residents from targeted communities who participated and are known and respected by people in the neighborhood.”	not reported	52% Female	Not reported	Not reported	Qualitative themes: community acceptance and participation; community mobilization; capacity building through life skills education, outreach peer education activities	Not applicable	-High rates of illiteracy, poverty, unemployment, mobility, and gender disparities place the predominantly young Yemeni nation at risk of HIV infection.<br>-Discussions re. knowledge and use of condoms, HIV risk and perceptions.	Not applicable (this was not the intervention study)	-Local council members highly appreciated the community peer education intervention because it helped to raise the HIV risk perception among community members.<br>-Controversy about male condoms (young males in the communities are more familiar and open to talk about condoms than females) still exist.<br>-Several targeted people had mentioned that the sessions influenced their behavior, while others considered the sessions boring, and others considered the “dose” of education not enough to convince them to change their	Related to study Ref Id:776

Refid	Author	Title	Abstract	User	Level	Author	Date	Jurisd	PubType	PubType_comment	Quant-CaseContr	Quant-CrossSect	Quant-Other	Quant-Other_comment	Qual-FocGroup	Qual-SemiStr	Mixed	Aim	Dates	InclExclCriteria	RecruitmentMethods	DataCollection	Participants	Participants_Youth	Age_mean	Gender_Percentage	Pop_Largest	SampleOther	Outcome	Comparator	RiskFactors	Intervention	MainFindings	Notes
			15 in-depth interviews were conducted among 52 participants: community peer educators, community focal points, targeted young people, and local councils. The results revealed that contributing factors to implementing the peer education program had been community participation, mobilization of targeted communities, and capacity building of all those included in the intervention. Existing commu nity-based organizations like the Social Service Centre played a key role in building trust with the targeted communities and linking HIV peer education intervention to other existing community																														risky behavior.<br>-FSWs all agreed that the peer education sessions were beneficial and informative, but there was little impact on their high-risk behavior<br>-Participant behaviour change: “I had been greatly impressed with the peer educators’ talk. I reviewed my behaviours in light of their explanation about risky behaviours and decided to change”	

Refid	Author	Title	Abstract	User	Level	Author	Date	Jurisd	PubType	PubType_comment	Quant-CaseContr	Quant-CrossSect	Quant-Other	Quant-Other_comment	Qual-FocGroup	Qual-SemiStr	Mixed	Aim	Dates	InclExclCriteria	RecruitmentMethods	DataCollection	Participants	Participants_Youth	Age_mean	Gender_Percentage	Pop_Largest	SampleOther	Outcome	Comparator	RiskFactors	Intervention	MainFindings	Notes
			services. This process evaluation could provide lessons learned for replicating similar youth peer education programs in conservative communities.																															
815	K. H. Nada, D. A. Suliman el	Violence, abuse, alcohol and drug use, and sexual behaviors in street children of Greater Cairo and Alexandria, Egypt	OBJECTIVES: To measure the prevalence of HIV/AIDS risk behaviors and related factors in a large, probability-based sample of boys and girls aged 12-17 years living on the streets of Egypt's largest urban centers of Greater Cairo and Alexandria. METHODS: Time-location sampling (TLS) was used to recruit a cross-sectional sample of street children. Procedures entailed using key informants and field observation to create a sampling frame	mohammad.alakel	5	Nada	2010	Greater Cairo and Alexandria, Egypt	PubPeer			Quant-CrossSect						To measure the prevalence of HIV/AIDS risk behaviors and related factors in a large, probability-based sample of boys and girls aged 12–17 years living on the streets of Egypt's largest urban centers.	summer of 2007	-The target population of our survey was street children, boys and girls, aged 12–17 years living in Greater Cairo and Alexandria.<br>-Excluded street children who were using the boarding facilities of nong overnmental organizations, as they were deemed systematically different from other street children not receiving such services.	-Time-location sampling (TLS) was used to recruit a cross-sectional sample of street children.	-A questionnaire was developed over a six month period of participant observation, and in-depth interviews with street children to help identify the topics to be included, how sensitive they were, terminologies, and possible responses.	N=857<br>(463) in Greater Cairo and (394) in Alexandria	N=443 (51.6%)<br>- In Greater Cairo, number of boys aged between 15 and 17 was 186 and the number of girls was 55<br>- In Alexandria, the number of boys aged between 15 and 17 was 183 and the number of girls was 19	Not reported	85% Males	-One-fifth (20%) of boys aged 15–17-year-old in Greater Cairo and more than one-third (37%) in Alexandria reported having sex with another male.<br>-62% had ever used drugs	93% faced harassment or abuse<br>67% of older adolescents were sexually active	Outcomes: Events of life on the street including violence and physical abuse, contact with family, knowledge of HIV/AIDS, drug/alcohol use, risky sexual behaviours, older age group asked: age of sexual debut, number of partners, same-sex partners, condom use, commercial sex, and forced sex.	Frequencies compared across age groups and cities.	Over one-third of the children (35%) reported current alcohol consumption, with use being more common among boys than among girls and more common among older than younger children. Ever use of drugs was reported by nearly two-thirds (62%) of all street-children, and the use of drugs at the time of data collection was reported by just over half (51%).<br>-Over half (53%) of female street children aged 15–17 years in Greater Cairo and 90% of those in	N/A	-Majority of street children have experienced some form of harassment or abuse, had ever used drugs and were sexually active. Many street children experienced more than one risk. Overlaps with populations at highest risk for HIV were substantial, namely men who have sex with men, commercial sex workers, and injection drug users.	

Refid	Author	Title	Abstract	User	Level	Author	Date	Jurisd	PubType	PubType_comment	Quant-CaseContr	Quant-CrossSect	Quant-Other	Quant-Other_comment	Qual-FocGroup	Qual-SemiStr	Mixed	Aim	Dates	InclExclCriteria	RecruitmentMethods	DataCollection	Participants	Participants_Youth	Age_mean	Gender_Percentage	Pop_Largest	SampleOther	Outcome	Comparator	RiskFactors	Intervention	MainFindings	Notes
			of locations at predetermined time intervals of the day, where street children congregate in the two cities, selecting a random sample of time-locations from the complete list, and intercepting children in the selected time-locations to assess eligibility and conduct interviews. Interviews gathered basic demographic information, life events on the street (including violence, abuse, forced sex), sexual and drug use behaviors, and HIV/AIDS knowledge. RESULTS: A total of 857 street children were enrolled in the two cities, with an age, sex, and time-location composition matching the sampling frame. The majority of these children																												Alexandria had a history of sexual abuse.<br>-The mean age at first intercourse with someone of the opposite sex was 14 years. Multiple sex partners in the preceding 12 months was reported by more than half (54%) of these older children. Also, more than half (52%) reported never using a condom, with only 20% reporting having used them consistently.			

Refid	Author	Title	Abstract	User	Level	Author	Date	Jurisd	PubType	PubType_comment	Quant-CaseContr	Quant-CrossSect	Quant-Other	Quant-Other_comment	Qual-FocGroup	Qual-SemiStr	Mixed	Aim	Dates	InclExclCriteria	RecruitmentMethods	DataCollection	Participants	Participants_Youth	Age_mean	Gender_Percentage	Pop_Largest	SampleOther	Outcome	Comparator	RiskFactors	Intervention	MainFindings	Notes
			had faced harassment or abuse (93%) typically by police and other street children, had used drugs (62%), and, among the older adolescents, were sexually active (67%). Among the sexually active 15-17-year-olds, most reported multiple partners (54%) and never using condoms (52%). Most girls (53% in Greater Cairo and 90% in Alexandria) had experienced sexual abuse. The majority of street children experienced more than one of these risks. Overlaps with populations at highest risk for HIV were substantial, namely men who have sex with men, commercial sex workers, and injection drug users. CONCLUSION:																															

Refid	Author	Title	Abstract	User	Level	Author	Date	Jurisd	PubType	PubType_comment	Quant-CaseContr	Quant-CrossSect	Quant-Other	Quant-Other_comment	Qual-FocGroup	Qual-SemiStr	Mixed	Aim	Dates	InclExclCriteria	RecruitmentMethods	DataCollection	Participants	Participants_Youth	Age_mean	Gender_Percentage	Pop_Largest	SampleOther	Outcome	Comparator	RiskFactors	Intervention	MainFindings	Notes
			Our study using a randomized TLS approach produced a rigorous, diverse, probability-based sample of street children and documented very high levels of multiple concurrent risks. Our findings strongly advocate for multiple services including those addressing HIV and STI prevention and care, substance use, shelters, and sensitization of authorities to the plight of street children in Egypt.																															
817	M. Hosseini, S. SeyedAlinaghi, P. Kheirandish, G. Esmaeli Javid, H. Shirzad, N. Karami, M. Jahani, M. Seyed Ahmadian, F. Payvarmehr, M. Mohraz, H.	Prevalence and correlates of co-infection with human immuno deficiency virus and hepatitis C virus in male injection drug users in Iran	OBJECTIVE: Aim of the study was to evaluate the HIV and hepatitis C virus (HCV) coinfection and associated risk behaviors among Injection Drug Users in Detention,	mohammad.alakel	5	Hosseini et al.,	2010	Tehran, Iran	PubPeer			Quant-CrossSect						To evaluate the HIV and hepatitis C virus coinfection and associated risk behaviors among injection drug users in Tehran, Iran.	02/2006	Not reported	- Participants included men who were arrested by police during a predetermined police sweep in Tehran, those identified as IDUs were approached consecutively	-A questionnaire was completed for each individual via a face-to-face interview by a physician.<br>-Blood specimens were collected by a professional phlebotomist for	N= 417	N= 58 for age between 17-24<br>N= 213 for age between 25-34	Range= (17 to > 45)	100% Male	100% IDU	74.6% did not complete high school<br>74.6% had history of incarceration<br>39.3% married, 38% single	-Demographic characteristics, imprisonment history, injecting drug practices, and sexual behaviors	N/A	-Factors independently associated with HIV/HCV coinfection included history of using opioid in jail, and age (P<0.05)*<br>-HIV/HCV coinfection was associated with	N/A	-Did not detect associations between HIV/HCV coinfection and other demographic characteristics, type of drugs used, injection practices, history of	

Refid	Author	Title	Abstract	User	Level	Author	Date	Jurisd	PubType	PubType_comment	Quant-CaseContr	Quant-CrossSect	Quant-Other	Quant-Other_comment	Qual-FocGroup	Qual-SemiStr	Mixed	Aim	Dates	InclExclCriteria	RecruitmentMethods	DataCollection	Participants	Participants_Youth	Age_mean	Gender_Percentage	Pop_Largest	SampleOther	Outcome	Comparator	RiskFactors	Intervention	MainFindings	Notes
	Emadi Koochak, W. McFarland		Tehran, Iran. METHODS: A cross-sectional survey included 499 male Injection Drug Users arrested by police during a predetermined police sweep in Tehran (February, 2006). At the temporary detention center, they were screened using a urine test and a physical examination for injection marks. Those who were identified as injectors were sent to the rehabilitation center for 3 months. A questionnaire was filled out for each individual by interview. Blood specimens were collected for HIV and HCV testing. The variables associated with HIV/HCV coinfection at a significance level of P<0.10																		prior to entering a rehab center to participate in the study.	HIV and HCV antibody testing for those who agreed to participate in the blood test									older age, being divorced, tattoos, past incarceration, opioid use in jail, and ever having sex.		sexually transmitted disease, or other sexual behaviors<br>-Of the 417 participants, 100 (24.0%) had HIV/HCV coinfection (95%CI 19.9 – 28.4). History of opioid use in jail and age remained significant, independent predictors of HIV/HCV coinfection	

Refid	Author	Title	Abstract	User	Level	Author	Date	Jurisd	PubType	PubType_comment	Quant-CaseContr	Quant-CrossSect	Quant-Other	Quant-Other_comment	Qual-FocGroup	Qual-SemiStr	Mixed	Aim	Dates	InclExclCriteria	RecruitmentMethods	DataCollection	Participants	Participants_Youth	Age_mean	Gender_Percentage	Pop_Largest	SampleOther	Outcome	Comparator	RiskFactors	Intervention	MainFindings	Notes
			were considered in multivariate analysis. RESULTS: Of the 417 participants, 100 (24.0%) had HIV/HCV coinfection (95%CI 19.9 - 28.4). Factors independently associated with HIV/HCV coinfection included history of using opioid in jail, and age (P<0.05). There were not any association between other demographic characteristics (marital status, birthplace, residence, and education), type and years of drug abuse, age of first injection, years of injection, sharing needles inside and outside of jail, injection in jail, history of tattooing, any sexual behavior, and history of sexually transmitted diseases with																															

Refid	Author	Title	Abstract	User	Level	Author	Date	Jurisd	PubType	PubType_comment	Quant-CaseContr	Quant-CrossSect	Quant-Other	Quant-Other_comment	Qual-FocGroup	Qual-SemiStr	Mixed	Aim	Dates	InclExclCriteria	RecruitmentMethods	DataCollection	Participants	Participants_Youth	Age_mean	Gender_Percentage	Pop_Largest	SampleOther	Outcome	Comparator	RiskFactors	Intervention	MainFindings	Notes
			HIV/HCV coinfection (P>0.05). CONCLUSIONS: This study supports that incarceration is contributing to the increased spread of HIV/HCV coinfection. So, there is urgent need for effective harm reduction programs, particularly among incarcerated Injection Drug Users.																															
855	J. G. Kahhaleh, M. El Nakib, A. R. Jurjus	Knowledge, attitudes, beliefs and practices in Lebanon concerning HIV/AIDS, 1996-2004	This cross-sectional study, aimed at evaluating the impact of HIV prevention interventions in Lebanon since 1996, was performed between January 2004 and July 2004 on 3200 Lebanese aged 15-49 years. Of the sexually active respondents, 13.0% of men and 2.6% of	mohammad.alakel	5	Kahhaleh	2009	Beirut, Lebanon	PubPeer			Quant-CrossSect						To evaluate the impact of HIV prevention interventions in Lebanon since 1996	01/2004 to 07/2004	All persons aged 15–49 years were included whether they were regular residents of the household or had spent the night before the interview in the household. Regular household members who were temporarily away from home were included as household	Households were selected for interviewing using systematic random sampling.	A survey using a stratified cluster sample design.	N= 3200	N= 2064 (age 15-34)	15–49 years	58.6% Male	64.5% were young Lebanese under the age of 35 years	-4% had no education at all<br>-27% are university graduate<br>-23% are married<br>-14.7% would be considered illiterate	(1) identification and demographic data,<br>(2) background characteristics, including source of information on health issues and media,<br>(3) marriage and regular partners,<br>(4) non-regular commercial sex,<br>(5) condoms,<br>(6) STDs and health issues (divided into 2 parts, one for men only and	Comparing sociodemographic parameters in 1996 versus 2004	-Use of condoms at the last intercourse was only 25.0% in 2004, similar to 23.7% in 1996<br>-Over 30% of those involved with non-regular partners stated that they gave or received money or gifts in exchange for sex (commercial sex); for 26.4% of these, the sexual encounter was during the first	N/A	The findings of the study showed that of the sexually active respondents, 13.0% of men and 2.6% of women had regular partners other than the spouse but only 25.0% used a condom in their last sexual intercourse. However, 16.8% had sex with casual partners and 71.7% of those used a	

Refid	Author	Title	Abstract	User	Level	Author	Date	Jurisd	PubType	PubType_comment	Quant-CaseContr	Quant-CrossSect	Quant-Other	Quant-Other_comment	Qual-FocGroup	Qual-SemiStr	Mixed	Aim	Dates	InclExclCriteria	RecruitmentMethods	DataCollection	Participants	Participants_Youth	Age_mean	Gender_Percentage	Pop_Largest	SampleOther	Outcome	Comparator	RiskFactors	Intervention	MainFindings	Notes
			women had regular partners other than the spouse but only 25.0% used a condom in their last sexual intercourse. However, 16.8% had sex with casual partners and 71.7% of those used a condom. Knowledge about preventive practices against HIV/AIDS has regressed since 1996, 85.7% compared to 94.9%. Self reports of symptoms suggestive of sexually transmitted disease were 9.1% compared to 5.6% in 1996.																	members and interviewed during a later visit.									the second for women only), (7) knowledge of AIDS, (8) risk perception, behaviour change and attitudes to persons with HIV/AIDS.		meeting.<br>-in 2004 37.9% of the sexually active respondents aged 15–24 years had casual sex and this decreased with age<br>-Self-reported symptoms of STD (pain during urination or discharge from penis) in the previous 12 months were reported by 12.4% of sexually active males in 2004<br>-Just over 35% of those reporting having symptoms of STD sought professional treatment in 2004, while 15.1% said they did nothing<br>-Younger respondents (< 25 years) who were not married and were sexually active constituted the category that had the highest rate of condom use during their		condom. Knowledge about preventive practices against HIV/AIDS has regressed since 1996, 85.7% compared to 94.9%. Self-reports of symptoms suggestive of sexually transmitted diseases were 9.1% compared to 5.6% in 1996.	

Refid	Author	Title	Abstract	User	Level	Author	Date	Jurisd	PubType	PubType_comment	Quant-CaseContr	Quant-CrossSect	Quant-Other	Quant-Other_comment	Qual-FocGroup	Qual-SemiStr	Mixed	Aim	Dates	InclExclCriteria	RecruitmentMethods	DataCollection	Participants	Participants_Youth	Age_mean	Gender_Percentage	Pop_Largest	SampleOther	Outcome	Comparator	RiskFactors	Intervention	MainFindings	Notes
																															last sexual intercourse, 34.6% (45 of 130 who ever used a condom), and the highest rates of having a regular partner, 25.7% -60.9% (78 of 128) of younger participants (<25 years) who had causal sexual intercourse in the last 12 months reported using a condom, this was less than all of the other age groups			
898	N. El-Sayyed, I. A. Kabbash, M. El-Gueniedy	Risk behaviours for HIV/AIDS infection among men who have sex with men in Cairo, Egypt	A sample of 73 men who have sex with men (MSM) in Cairo, Egypt, were screened for HIV infection and were interviewed to study their risk behaviours for HIV/AIDS. Most (65.8%) had initiated sexual activity before 15 years; 65.8% took both active and passive roles in sexual	mohammad.alakel	5	El-Sayyed	2008	Cairo, Egypt	PubPeer			Quant-CrossSect						To study HIV/AIDS risk behaviors of men who have sex with men in Cairo, Egypt.	2003	Not reported (being MSM).	-MSM volunteers succeeded in bringing groups of MSM to be interviewed individually.<br>-All participants (n = 73) were recruited by the snowball technique during a period of 8 months of fieldwork.	A predesigned and pretested questionnaire sheet was used for data collection. The questionnaire included the following data:	N= 73	N= 58	-Age range was 15–47 years<br>-79.5% aged 15-24	100% Male	100% MSM	- Not married (84.9%)<br>-Working in manual work (71.2%)<br>-Most (79.5%) had received primary or secondary education	-MSM behaviour (age at first sexual experience, type of sexual practices (ever used condom or asked partner to use it during last 3 months) number of partners/week, frequency of sexual acts and whether with single or multiple partners, and heterosexuality);	-One-third of older MSM (33.3%) had only 1 sexual partner per sex act compared with 15.5% among the younger group.<br>-Comparing old MSM vs. Young MSM<br>-Knowledge, attitude and practices of men who have sex with men compared across	-The age at which homosexual relations was initiated was < 15 years among 65.8% of respondents<br>-Respondents aged < 25 years were found to be more sexually active; 25.9% had sex more than once per day compared with only<br>6.7% among	N/A	-Educational level was found to have its effect on the knowledge about risks and protective measures for HIV/AIDS. The majority of the study group were illiterate or of only primary education which means that they are at high risk for<br>HIV infection.<br>-Most	

Refid	Author	Title	Abstract	User	Level	Author	Date	Jurisd	PubType	PubType_comment	Quant-CaseContr	Quant-CrossSect	Quant-Other	Quant-Other_comment	Qual-FocGroup	Qual-SemiStr	Mixed	Aim	Dates	InclExclCriteria	RecruitmentMethods	DataCollection	Participants	Participants_Youth	Age_mean	Gender_Percentage	Pop_Largest	SampleOther	Outcome	Comparator	RiskFactors	Intervention	MainFindings	Notes
			acts. The frequency of sexual acts was < 1 per week for 73.3% of those aged 25+ years, but > 1 daily for 25.9% of those aged < 25 years. Heterosexual relations were reported by 73.3% of the older age group, while 70.7% of the younger age group were exclusively MSM. Condoms were always used by only 19.2% of the sample.																										Use of alcohol and illicit drugs; past and present history of any manifestations related to sexually transmitted infections (STIs).<br>-Ever complained of discharge, painful micturition or defecation or ulcers on sexual organs during last 6 months<br>-Ever complained of discharge from urethral or anal canal or ulcers on sexual organs during last 6 months	education levels.	those 25+ years<br>-5.2% of younger persons had > 8 sexual partners per week compared to 0% of the older group<br>-Use of illicit drugs was reported by 31.5% of the study group with no statistically significant difference in relation to age<br>-The most commonly used drugs were cannabis herb (bango) (21.9%),<br>-Cannabis herb was used by more of the younger age group (24.1%) than the older age group (13.3%)<br>-Condoms were never used by just over half of the studied sample (52.1%); 28.8% sometimes used them, while only 19.2% reported always using condoms during sexual relations		participants (65.8%) had initiated sexual activity before 15 years; 65.8% took both active and passive roles in sexual acts. The frequency of sexual acts > 1 daily for 25.9% of those aged < 25 years. Condoms were always used by only 19.2% of the sample. Condom use increased with increased educational level.	

Refid	Author	Title	Abstract	User	Level	Author	Date	Jurisd	PubType	PubType_comment	Quant-CaseContr	Quant-CrossSect	Quant-Other	Quant-Other_comment	Qual-FocGroup	Qual-SemiStr	Mixed	Aim	Dates	InclExclCriteria	RecruitmentMethods	DataCollection	Participants	Participants_Youth	Age_mean	Gender_Percentage	Pop_Largest	SampleOther	Outcome	Comparator	RiskFactors	Intervention	MainFindings	Notes
																															-Always-use condoms was reported by significantly more of those aged 25+ years (40.0%) than those < 25 years (13.8%) (?2 = 5.28, P = 0.022)*<br>-Heterosexual relations were reported by 73.3% of the older age group, while 70.7% of the younger age group were exclusively MSM (p=0.002)*			
902	N. El-Sayyed, I. A. Kabbash, M. El-Gueniedy	Knowledge, attitude and practices of Egyptian industrial and tourist workers towards HIV/AIDS	This study explored knowledge, attitudes and practices towards HIV/AIDS infection among 1256 Egyptian industrial and tourism workers aged 16-40 years. Compared with industrial workers, tourism workers had a significantly better perception of the magnitude of the HIV/AIDS	mohammad.alakel	5	El-Sayyed	2008	Mahalla El-Koubra, 10th of Ramadan and Kafr El- Dawar, Luxor city, South Sinai tourist resorts and Red Sea tourist resorts, Egypt	PubPeer			Quant-CrossSect						To explore knowledge, attitudes and practices towards HIV/AIDS infection among 1256 Egyptian industrial and tourism workers aged 16–40 years	Not reported	Industrial workers in the textile industry in 3 large industrial cities in Egypt; and tourism workers in 3 main tourist locations in Egypt.	A list of factories and tourist establishments located in the selected towns and the total number of workers in each location were obtained.<br>A sample of 3 factories and 3 tourist establishments were randomly selected from places accommodating more than 100 workers.<br>Workers in these areas	A predesigned questionnaire sheet was used for data collection.	N= 1256	-Among industrial workers, those aged < 22 years represented 35.8% as compared to 9.6% among tourism workers. N= 570<br>-Industrial workers (n=543) age 16-30; Tourism workers (n=627) age 16-30; Total (n=1170) (93% of total)	Range 16-40	80% male	(49.7%) industrial workers and 632 (50.3%) tourism workers	60.4% of workers were unmarried	-Sociodemographic data, including age, sex, marital status, educational level, occupation and residence.<br>-Knowledge about the epidemiological aspects of HIV/AIDS, including the magnitude of the problem worldwide and in Egypt (low, moderate or high prevalence of HIV),	-Compared with industrial workers, tourism workers had a significantly better perception of the magnitude of the HIV/AIDS problem worldwide as well as in Egypt and of the likelihood of the problem worsening<br>-The majority of tourism workers (61.2%) were aged 26–30 years as compared to	-Only 2.8% and 1.4% of both groups reported avoidance of illicit drugs and limiting the number of sexual partners respectively.<br>-Use of condoms was reported by 0.2% of industrial workers and 0.6% of tourism workers<br>-Illiteracy was higher among industrial workers (14.9%) compared to	N/A	-Knowledge of tourism workers was also significantly better about causative agent of AIDS and methods of transmission.<br>-Both groups had negative attitudes towards patients living with HIV/AIDS concerning their right to confidentiality and to work.<br>-Both groups had a positive	

Refid	Author	Title	Abstract	User	Level	Author	Date	Jurisd	PubType	PubType_comment	Quant-CaseContr	Quant-CrossSect	Quant-Other	Quant-Other_comment	Qual-FocGroup	Qual-SemiStr	Mixed	Aim	Dates	InclExclCriteria	RecruitmentMethods	DataCollection	Participants	Participants_Youth	Age_mean	Gender_Percentage	Pop_Largest	SampleOther	Outcome	Comparator	RiskFactors	Intervention	MainFindings	Notes
			problem worldwide as well as in Egypt and of the likelihood of the problem worsening. Knowledge of tourism workers was also significantly better about causative agent of AIDS and methods of transmission. Both groups had negative attitudes towards patients living with HIV/AIDS concerning their right to confidentiality and to work. Both groups had a positive attitude towards behaviour change for protection from HIV/AIDS, principally via avoidance of extramarital sexual relations and adherence to religious beliefs. Use of condoms as a way to avoid HIV/AIDS was reported by only																		were divided into small clusters according to the circumstances of each place. A number of clusters were randomly chosen from each place to represent 10%–20% of the total population of that place.								methods of transmission of infection, clinical manifestations and knowledge about treatment of patients.<br>-Attitudes and practices towards HIV/ AIDS and behaviour changes that could take place in response to HIV/AIDS risk of infection.	29.8% among industrial workers<br>-Workers aged 31–40 years represented 13.0% among industrial workers as compared to only 0.8% among tourism workers.<br>-The sex distribution was also significantly different in the 2 groups; females represented 36.2% of industrial workers as compared to 4.4% among tourism workers (?2 = 196.65, P < 0.001)<br>-Changes in behaviours compared between industrial and tourism workers.	tourism workers (1.7%) and the percentage of those with higher education was 28.3% among tourism workers compared to only 5.1% of industrial workers (?2= 235.32, P < 0.001)*<br>-The majority of industrial workers were manual workers (71.2%) while nearly one-half of tourism workers were employees (50.3%) (?2 = 238.03, P< 0.001)<br>-The majority of industrial workers lived with their families (94.3%) while the majority of tourism workers lived away from their families with friends (65.5%) (?2 = 512.97, P < 0.001)<br>-Concerning knowledge about the magnitude of		attitude towards behaviour change for protection from HIV/AIDS, principally via avoidance of extramarital sexual relations and adherence to religious beliefs.	

Refid	Author	Title	Abstract	User	Level	Author	Date	Jurisd	PubType	PubType_comment	Quant-CaseContr	Quant-CrossSect	Quant-Other	Quant-Other_comment	Qual-FocGroup	Qual-SemiStr	Mixed	Aim	Dates	InclExclCriteria	RecruitmentMethods	DataCollection	Participants	Participants_Youth	Age_mean	Gender_Percentage	Pop_Largest	SampleOther	Outcome	Comparator	RiskFactors	Intervention	MainFindings	Notes
			0.4% of workers.																												the problem of HIV/AIDS, significantly more tourism workers than industrial workers reported AIDS as the most serious disease worldwide (86.6% versus 79.7%) (?2 = 10.66, P < 0.001)<br>-Similar proportions of workers of both groups reported AIDS as the most serious problem in Egypt (32.4% for industrial and 36.1% for tourism workers).<br>-Perception of the importance of liver diseases as a serious problem was significantly higher among tourism workers (33.2%) compared to industrial workers (23.6%) (?2 = 14.43, P < 0.001).<br>-Knowing the magnitude of the problem of			

Refid	Author	Title	Abstract	User	Level	Author	Date	Jurisd	PubType	PubType_comment	Quant-CaseContr	Quant-CrossSect	Quant-Other	Quant-Other_comment	Qual-FocGroup	Qual-SemiStr	Mixed	Aim	Dates	InclExclCriteria	RecruitmentMethods	DataCollection	Participants	Participants_Youth	Age_mean	Gender_Percentage	Pop_Largest	SampleOther	Outcome	Comparator	RiskFactors	Intervention	MainFindings	Notes
																															HIV/AIDS and its likelihood to get worse in the future was significantly better among tourism workers compared to industrial workers (?2= 16.81 and 6.71 respectively, P < 0.01).			
915	F. Ramezani Tehrani, H. Malek-Afzali	Knowledge, attitudes and practices concerning HIV/AIDS among Iranian at-risk sub-populations	This study in 2003 looked at knowledge, attitudes and behaviours concerning HIV among 3 high-risk groups (201 cross-border truck drivers, 50 female sex workers and 754 youths) in 4 cities in the Islamic Republic of Iran. The level of knowledge about HIV was low on average, especially among individuals with high-risk behaviours. Truck drivers and female sex workers had higher knowledge	mohammad.alakel	5	Tehrani	2008	Saravan, Astara, Islamshahr and Kermanshah, Iran	PubPeer			Quant-CrossSect						To look at knowledge, attitudes, and behaviors concerning HIV among 3 high-risk groups (201 cross-border truck drivers, 50 female sex workers, and 754 youths) in 4 cities in the Islamic Republic of Iran.	2003	Not reported	-The sample of youths was recruited from the list of total households of each city using a modified stratified, multistage cluster sampling approach (the segmentation method)<br>-For cross-border truck drivers, a randomized sample of individuals could not be recruited thus a viable sampling strategy was to randomly select registration numbers and then recruit truck drivers present with	An anonymous questionnaire including closed questions was used.	N= 754	N= 754	Range: 15-25	Not reported	3 high-risk groups (truck drivers, female sex workers, and youths),	-62.2% of youth are single	-Ever seen somebody use inhaled drugs<br>-Ever seen somebody use injected drugs<br>-Ever had a previous history of STI	Comparing knowledge, attitudes, and behaviors concerning HIV among 3 high-risk groups (truck drivers, female sex workers, and youths)	-Truck drivers were less knowledgeable about HIV/AIDS than were the youths<br>- 27.9% of youth had a previous STI<br>-The majority of respondents with a previous history of STIs from all of these 3 groups were not aware about the need to treat sexual partners<br>-More than one-third of sexually active truck drivers and male youths (35.2%) had never used condoms. Reduction of sexual pleasure was acknowledged	N/A	-Condom use was low in all groups, with (35.2%) of youth reporting having never used condoms.<br>-Very few youth (14.2%) were concerned about HIV infection and less than 1% of them had been tested for HIV before.	we focused on results for youth as they were the youngest.

Refid	Author	Title	Abstract	User	Level	Author	Date	Jurisd	PubType	PubType_comment	Quant-CaseContr	Quant-CrossSect	Quant-Other	Quant-Other_comment	Qual-FocGroup	Qual-SemiStr	Mixed	Aim	Dates	InclExclCriteria	RecruitmentMethods	DataCollection	Participants	Participants_Youth	Age_mean	Gender_Percentage	Pop_Largest	SampleOther	Outcome	Comparator	RiskFactors	Intervention	MainFindings	Notes
			about sexually transmitted infections than youths but their knowledge came primarily from personal experience rather than public awareness programmes. Truck drivers had a more positive attitude to temporary marriage and pre- and extramarital sex than youths. Condom use was low in all groups. Better targeted education of high-risk groups is needed.																		those numbers on the recruitment days<br>-For FSWs a modified respondent-driven sampling method was selected										-as the most common reason (42%) for not using condom among youths.<br>-12.4% of male youths had a previous history of extramarital or premarital sex and 38.3% of truck drivers<br>-Very few youths (14.2%) were concerned about HIV infection and less than 1% of them had been tested for HIV before			
938	M. Hajiabdolbaghi, N. Razani, N. Karami, P. Kheirandish, M. Mohraz, M. Rasoolinejad, K. Arefnia, Z. Kourorian, G. Rutherford, W. McFarland	Insights from a survey of sexual behavior among a group of at-risk women in Tehran, Iran, 2006	Despite high rates of HIV among male injection drug users, the sexual behaviors of at-risk women in Iran remain unknown. A questionnaire on HIV knowledge and risk behavior was administered in	mohammad.alakel	5	Hajiabdolbaghi	2006	Tehran, Iran	PubPeer				Quant-Other	Intervention				To gain an understanding of sexual and substance use behaviours and HIV/AIDS knowledge among at-risk young women (who were runaways/seeking safe haven) in Tehran, Iran.	Not reported	Women at a Tehran-based NGO.	Women were recruited for this study through a Tehran-based NGO that provides shelter and occupational training for runaway women and girls in Tehran. Also included in our sample was the peer education staff, which had	Data were collected via a questionnaire administered before and after an HIV educational session. HIV and syphilis testing.	N= 50	N=33	50% <24 years old.	100% Female	-Some high–risk women had poly–substance abuse and high–risk sexual behaviors<br>-100% women at-risk.	-The majority of participants were from an urban area (82%), had never been married (64%), and were employed (60%).<br>-6% reported that an unrelated man supported them.<br>-64% never married	-Sexual and substance use behaviors, and HIV/AIDS knowledge.<br>-Ever had sexual contact<br>-Ever used alcohol<br>-Ever used cigarettes<br>-Ever used any other illicit substance	Compare knowledge of HIV scores pre/post-test.	-Respondents had high knowledge about condom use but did not act on their knowledge.<br>-Previously testing for HIV was reported by 7% of single women, 31% of married women, and 50% of those whose marital status	Pretest on HIV/AIDS Knowledge. Intervention: A 4–hour educational session on HIV/AIDS conducted by the Iranian Research Center for HIV/AIDS followed by a post-test.	-Few of the respondents listed kissing or touching as their only contact but still considered this as sex<br>-Respondents had high knowledge about condom use but did not act on their knowledge<br>-Half admitted to a history of	

Refid	Author	Title	Abstract	User	Level	Author	Date	Jurisd	PubType	PubType_comment	Quant-CaseContr	Quant-CrossSect	Quant-Other	Quant-Other_comment	Qual-FocGroup	Qual-SemiStr	Mixed	Aim	Dates	InclExclCriteria	RecruitmentMethods	DataCollection	Participants	Participants_Youth	Age_mean	Gender_Percentage	Pop_Largest	SampleOther	Outcome	Comparator	RiskFactors	Intervention	MainFindings	Notes
			a Tehran nongovernmental organization targeting runaways and other women seeking safe haven. Half (total N = 50) were less than 24 years old; baseline HIV knowledge was high. The few who acknowledged using illicit substances said they used "frequently." Nonresponse rates to questions regarding sexual behavior were high (12 of 50). Half admitted a history of sexual activity; 40% of those reported their first sexual contact with someone other than their husband; three people had multiple partners. Three women reported a history of rape. Zero (97.5% one-sided																	past histories of commercial sex work							-84% Heterosexual women<br>-4% Homosexual women<br>-48% had answered marriage as the reason for having sex<br>-52% answered yes for vaginal sex<br>-4% answered yes for anal sex<br>-42% answered "sometimes" for the frequency of using condom			was unknown.<br>-Of the women who had a history of sexual contact, eight (38%) said they had no information about STDs but had received education regarding HIV/AIDS.<br>-About half the women (46%) said they knew how to use a condom.<br>-70% thought that using a condom was protective during sex, but only 22% of those who were sexually active said they had used condoms during sex in the last year.<br>-Educational level was not a significant predictor of condom use.		sexual activity; 40% of those reported their first sexual contact with someone other than their husband; three people had multiple partners. 70% thought that using a condom was protective during sex, but only 22% of those who were sexually active said they had used condoms during sex in the last year. Educational level was not a significant predictor of condom use.	

Refid	Author	Title	Abstract	User	Level	Author	Date	Jurisd	PubType	PubType_comment	Quant-CaseContr	Quant-CrossSect	Quant-Other	Quant-Other_comment	Qual-FocGroup	Qual-SemiStr	Mixed	Aim	Dates	InclExclCriteria	RecruitmentMethods	DataCollection	Participants	Participants_Youth	Age_mean	Gender_Percentage	Pop_Largest	SampleOther	Outcome	Comparator	RiskFactors	Intervention	MainFindings	Notes
			confidence interval [CI] = 0, 0.17) of 35 women tested positive for HIV or syphilis. This study documents the existence of sexual behavior in a population of Iranian women, represents one of the first attempts at sexual research in the Iranian context, and highlights challenges in surveying this vulnerable group.																															
947	K. Mohammad, F. K. Farahani, M. R. Mohammadi, S. Alikhani, M. Zare, F. R. Tehrani, A. Ramezankhani, A. Hasanzadeh, H. Ghanbari	Sexual risk-taking behaviors among boys aged 15-18 years in Tehran	PURPOSE: The purpose of this study is to evaluate the extent and potential correlates of sexual risk-taking behaviors among adolescent boys aged 15-18 in metropolitan Tehran. METHODS: Data were collected from a population-based, cross-	mohammad.alakel	5	Mohammad et al.,	2007	Tehran, Iran	PubPeer			Quant-CrossSect						To evaluate the extent and potential correlates of sexual risk-taking behaviors among adolescent boys aged 15–18 in metropolitan Tehran.	07/ 2002 to 09/ 2002	Data for this study were gathered from never-married boys aged 15–18 residing in Tehran, Iran.	The sample was derived through cluster sampling of the 22 main municipality regions of Tehran	Data were collected via anonymous, self-administered questionnaire	Whole sample N=1385 Sexually experienced N=382 (sample focused on in study)	N=382	Age Range= 15 to 18	100% Males	Adolescent boys Approximately 28% of initial sample (382/1385) reported any lifetime sexual experience	68% of sexually experienced adolescents reported either having multiple sexual partners or non-use of condom	Any lifetime sexual experience, age at first sex, condom and contraceptive use, feelings of regret about sexual experiences, and information about the first sexual partner were requested.	-Compared risk awareness and beliefs by sexual experience and protection behavior<br>-Compared factors associated with condom non-use and multiple sexual partners	-Most (68%) of sexually experienced adolescents reported either having multiple sexual partners or non-use of condom.<br>-Younger adolescents (68%) reported significantly less condom use than older adolescents (78%).<br>-Access to Internet was	N/A	Having no access to Internet, feeling regretful at sexual debut, having one sexual partner in lifetime and lower knowledge of condoms are predictors of condom non-use. Older age, using alcoholic drinks, early sexual debut, and poor knowledge of	

Refid	Author	Title	Abstract	User	Level	Author	Date	Jurisd	PubType	PubType_comment	Quant-CaseContr	Quant-CrossSect	Quant-Other	Quant-Other_comment	Qual-FocGroup	Qual-SemiStr	Mixed	Aim	Dates	InclExclCriteria	RecruitmentMethods	DataCollection	Participants	Participants_Youth	Age_mean	Gender_Percentage	Pop_Largest	SampleOther	Outcome	Comparator	RiskFactors	Intervention	MainFindings	Notes
			sectional survey of adolescent males (ages 15-18) residing in Tehran, Iran. Of 1385 subjects, 382 reported sexual experience (27.7%). The article is mainly considering sexually experienced subjects. Two factors were considered as sexual risk-taking behavior ("not using condom or inconsistent condom use in sexual contacts" and "ever had multiple sexual partners in lifetime." Several other factors were considered as independent variables and their relations were assessed using t test, chi2 test, and logistic regression models.<br>RESULTS: Having no access to Internet, feeling regretful at																											associated with increased condom use (< .05*).<br>-The odds of condom non-use among adolescents who were not aware of the protective role of condoms against STI and HIV was 2.24 compared with those who were aware (p < .002)*<br>-Those adolescents who reported alcohol use also reported more condom use (80% vs. 72%) and having multiple partners (80% vs. 68%) compared with non-users of alcohol.<br>-About 13% of those who reported consistent condom use did not know that condoms should not be used more than once, whereas 31% of condom non-users did not		reproductive physiology are predictors of multiple sexual partners among adolescent boys aged 15–18 years.	

Refid	Author	Title	Abstract	User	Level	Author	Date	Jurisd	PubType	PubType_comment	Quant-CaseContr	Quant-CrossSect	Quant-Other	Quant-Other_comment	Qual-FocGroup	Qual-SemiStr	Mixed	Aim	Dates	InclExclCriteria	RecruitmentMethods	DataCollection	Participants	Participants_Youth	Age_mean	Gender_Percentage	Pop_Largest	SampleOther	Outcome	Comparator	RiskFactors	Intervention	MainFindings	Notes
			sexual debut, having one sexual partner in lifetime and lower knowledge of condoms are predictors of condom non-use. Older age, using alcoholic drinks, early sexual debut, and poor knowledge of reproductive physiology are predictors of multiple sexual partners among adolescent boys aged 15-18 years. CONCLUSION: Appropriate interventional programs should be implemented for adolescents in Iran to encourage and enable them to delay first sex and abstain unwanted and unplanned penetrative sex, to stress the health risks of alcohol use in terms of sexual health and finally to																											know this fact ($p < .001$)*<br>-18% of condom users did not know about the efficacy of condoms in prevention of pregnancy, compared with 38% among condom non-users ($p <.001$)*			

Refid	Author	Title	Abstract	User	Level	Author	Date	Jurisd	PubType	PubType_comment	Quant-CaseContr	Quant-CrossSect	Quant-Other	Quant-Other_comment	Qual-FocGroup	Qual-SemiStr	Mixed	Aim	Dates	InclExclCriteria	RecruitmentMethods	DataCollection	Participants	Participants_Youth	Age_mean	Gender_Percentage	Pop_Largest	SampleOther	Outcome	Comparator	RiskFactors	Intervention	MainFindings	Notes
			enhance their knowledge on different aspects of reproductive health particularly prevention against STI/HIV.																															
1027	A. Refaat	Practice and awareness of health risk behaviour among Egyptian university students	Health risk behaviour contributes markedly to today's major killers. A descriptive cross-sectional study was conducted to assess current awareness and practice of health risk behaviour among Egyptian university students. Only 121 students (18%) were practising risky behaviour. Tobacco use, alcohol and drugs use and risky sexual behaviour were positively correlated. Multiple regression analysis revealed that the main determinants of	mohammad.alakel	5	Refaat	2004	Ismailia, Egypt	PubPeer			Quant-CrossSect						To assess current awareness and practice of health risk behaviour among Egyptian university students	Not reported	Students at Sucz Canal University main campus in Ismailia. in their first and second year.	The schools were stratified according to type of study. one school was randomly selected from each study type. Students were randomly allocated from student lists.	A questionnaire was used to collect data	N= 687	N= 687	The median age of students was 18 years (54.7% ?18 and 45.3% ? 19)	59.0% were female	-Male, of older age, having a high allowance and having no attention to danger.	-71% urban residents<br>-98.8% never married<br>-12.1% of students' fathers were uneducated<br>-25.7% of students' mothers were uneducated<br>-17.7% of students were working in addition to their study	-Tobacco use: current smokers of cigarettes or water pipes or others<br>-Alcohol and substance use: current users of alcohol or other substances<br>-Unsafe sexual behaviour: having multiple partners, or sex without contraceptives or protection from sexually transmitted infection (STI)	Compared socio demographic characteristics across students practicing different types of risk behaviours.	-10% of participants reported ever having sex, median age for starting sex was 16 years old.<br>-Of the 32 participants who answered the question on contraception at last sexual encounter, 12 (37.5%) did not use any contraception.<br>-Of the 33 participants who answered the question on drug/alcohol use, 10 (30.3%) had alcohol or drugs before their last sexual encounter.<br>-Median number of sexual partners in last 3 months was 2.<br>-Current use of alcohol or drugs was reported in 5% of students:	N/A	-Only 121 students (18%) were practicing risky behaviour, tobacco use, alcohol and drug use, and risky sexual behaviour were positively correlated.<br>-30% of students lacked adequate knowledge of AIDS.<br>-Most of thse who had sexual relationships did not use contraceptives or any method of protection from sexually transmitted infections.<br>-Main sources of knowledge were the media (38%) then peers (30%)	

Refid	Author	Title	Abstract	User	Level	Author	Date	Jurisd	PubType	PubType_comment	Quant-CaseContr	Quant-CrossSect	Quant-Other	Quant-Other_comment	Qual-FocGroup	Qual-SemiStr	Mixed	Aim	Dates	InclExclCriteria	RecruitmentMethods	DataCollection	Participants	Participants_Youth	Age_mean	Gender_Percentage	Pop_Largest	SampleOther	Outcome	Comparator	RiskFactors	Intervention	MainFindings	Notes
			risky behaviour were being a male, of older age, having a high allowance and having no attention to danger. About 30% of students lacked adequate knowledge on AIDS. Most of those who had sexual relationships did not use contraceptives or any method of protection from sexually transmitted infection. Main sources of knowledge were the media (38%) then peers (30%).																												more among older students, males, those with higher allowance or were working.<br>- Statistically significant correlation between the three types of risk behaviours (sexual, tobacco use, alcohol/drug use).*<br>-Main determinants of risky behaviour was being male, older, having a high allowance and paying no attention to danger.<br>-58.0% of students had moderate awareness of the dangers of risky health behaviors while 25.9% had no awareness at all<br>-Alcohol was ever tried bg 14.4% of the students questioned, median age 17 years for first use.<br>-Currently only 4.1% were			

Refid	Author	Title	Abstract	User	Level	Author	Date	Jurisd	PubType	PubType_comment	Quant-CaseContr	Quant-CrossSect	Quant-Other	Quant-Other_comment	Qual-FocGroup	Qual-SemiStr	Mixed	Aim	Dates	InclExclCriteria	RecruitmentMethods	DataCollection	Participants	Participants_Youth	Age_mean	Gender_Percentage	Pop_Largest	SampleOther	Outcome	Comparator	RiskFactors	Intervention	MainFindings	Notes
																															drinking alcohol<br>-Only 8 students ever used intravenous drug injection at median age of 13 years for first use and only 3 students were still using it daily<br>-Ever using stimulating frugs was reported by 9.3% while 18.2% ever sniffed substances such as benzene, paints and sprays<br>-10% ever ha dsex, median age starting at 16 years<br>-61,5% did nothing to protect themselves from STI			
1069	S. M. Adib, S. Akoum, S. El-Assaad, A. Jurjus	Heterosexual awareness and practices among Lebanese male conscripts	To assess sexual behaviour and awareness about sexually transmitted infections (STIs) among young male adults in Lebanon, and to explore determinants associated with increasing	mohammad.alakel	5	Adib	2002	Beirut, Lebanon	PubPeer			Quant-CrossSect						To assess sexual behaviour and awareness about sexually transmitted infections (STIs) among young male adults in Lebanon and to explore determinants associated with increasing	1999	Young Lebanese men born between 1 January 1973 and 31 December, 1980, who were mentally and physically able and called to serve in 1999	Conscripts were selected directly from the name rosters of each camp. Every tenth name was selected sequentially for participation, to obtain 730 names. All selected conscripts were approached,	Data was collected through a structured questionnaire completed through face to face interviews.	N= 730	N= 730	Age ranged between 19 and 26, mean of 21.2 and a median of 21 years	100% Males	Young Lebanese men born between 1 January 1973 and 31 December, 1980	-95.5% single<br>-50% Reported any lifetime heterosexual experience<br>-51% resided in urban areas<br>-44.5% were poorly educated<br>-17.5% illiterate<br>-85% had held some type of employment	-Age, education and parental education (3 levels)<br>-Employment prior to conscription (yes/no)<br>-Crowding in the household (persons/room)<br>-Place of residence (urban/rural)	N/A	-50% of participants reported any sexual experience. Number of partners in the past year ranged from 0 to 50 with a mean of 3.5 (SD 5.6).<br>-Age of first sexual activity ranged from	N/A	-Non-mutually exclusive sex predominated, and only half of the respondents reported consistent condom use during insertive vaginal sex.<br>-Higher sexual risk-taking was associated with urban	

Refid	Author	Title	Abstract	User	Level	Author	Date	Jurisd	PubType	PubType_comment	Quant-CaseContr	Quant-CrossSect	Quant-Other	Quant-Other_comment	Qual-FocGroup	Qual-SemiStr	Mixed	Aim	Dates	InclExclCriteria	RecruitmentMethods	DataCollection	Participants	Participants_Youth	Age_mean	Gender_Percentage	Pop_Largest	SampleOther	Outcome	Comparator	RiskFactors	Intervention	MainFindings	Notes
			levels of sexual risk, a cross-sectional survey of 730 conscripts was conducted. About 50% reported any lifetime heterosexual experience. Non-mutually exclusive sex predominated, and only half of the respondents reported consistent condom use during insertive vaginal sex. Higher sexual risk-taking was associated with urban residence, higher education, lower family crowding and younger age at first sexual experience. A national strategy with epidemiological and behavioural surveillance and educational programmes must be initiated before sexual risk-taking and incidence of															levels of sexual risk			and only 10 refused to participate or were physically not available at the time of the interview. They were replaced by other randomly selected names from the corresponding camps.								-Ever had sex and had sex in 1998<br>-Those who had ever had any sexual activity, age at first encounter<br>-Type of sexual practice<br>-The frequency of condom use and causes of inconsistent condom use were assessed. Rating on a 4 level index of sexual risk.		13-24 years.<br>-Increasing risk levels was significantly* associated with increasing age, SES index. High risk was associated with higher education, lower crowding, and urban residence, younger age at first sexual experience*. In a multivariate model the most significant predictors of risk were lower crowding and higher education.<br>-Main reason for inconsistent condom use was confidence in partner/having an exclusive partner (n=123/143; 86.0%).<br>-40% of the participants reported at least one experience with insertive vaginal sex.<br>-Of that 40 %, 31% reported inconsistent		residence, higher education, lower family crowding and younger age at first sexual experience.	

Refid	Author	Title	Abstract	User	Level	Author	Date	Jurisd	PubType	PubType_comment	Quant-CaseContr	Quant-CrossSect	Quant-Other	Quant-Other_comment	Qual-FocGroup	Qual-SemiStr	Mixed	Aim	Dates	InclExclCriteria	RecruitmentMethods	DataCollection	Participants	Participants_Youth	Age_mean	Gender_Percentage	Pop_Largest	SampleOther	Outcome	Comparator	RiskFactors	Intervention	MainFindings	Notes
			STIs become a major public health issue.																												condom use<br>-18% never used condom<br>-Among the 367 young men with any sexual experience, the number of partners reported in the previous year ranged between 0 and 50, with a mean of 3.4<br>-42.5% believed their partner(s) had sex with other men, and 30% did not know or were not sure			
1078	K. Hendrickx, E. Lodewijckx, P. Van Royen, J. Denekens	Sexual behaviour of second generation Moroccan immigrants balancing between traditional attitudes and safe sex	Young Moroccan Islamic immigrants are balancing the challenges of modern society and the influences of their cultural and social backgrounds. Prevention and information programs need insights into their knowledge, attitudes and behaviour concerning choice of partner,	mohammad.alakel	5	Hendrickx	2002	Antwerp, Belgium	PubPeer						Qual-FocGroup			To obtain information directly from Moroccan adolescents about their expectations concerning marriage, choice of partner and family planning, and their attitudes related to premarital sexuality, contraception use, safe sex and prevention of STDs and AIDS.	06/1997 and 06/1998	Second generation Moroccan youth o the second generation, were between 15 and 21 years old, unmarried, and from Berber or Arabic speaking families, able to speak in Dutch.	Participants were recruited from Moroccan youth centres and a family practice in Antwerp	Data were collected during the focus groups	N= 55	N= 55	The age distribution was between 15 and 21 years, with an average age of 17–18.	27 Moroccan boys and 28 Moroccan girls Thus, 51% females	Moroccan youth in Antwerp, Belgium	-100% unmarried<br>-Forty-four had lived in Belgium since birth, or since the first year of life.<br>-Half of them were of Arabic origin and half of Berber origin.<br>-Forty-four were currently going to school: mostly in lower secondary, 12 in higher secondary, and 1 in university.<br>-The number of brothers and sisters was in	Expectations of marriage, family planning, sexual behaviour, contraception, STD and AIDS.	N/A	-All of the participants had heard about AIDS at school, on television or in the media, but the information is often incomplete or incorrect<br>-There was a lot of confusion about the means of contamination and about symptoms. In regard to other STDs, it was even worse.<br>-According to these boys, all	N/A	-Adolescents have limited knowledge of contraceptives, STD and AIDS. Some boys pretend to perform safe sex in certain ‘‘unfixed’’ circumstances but show no concern about the possible risks for future virgin spouses.<br>-Most of the girls do not consider safe sex before or after marriage.<br>-There is a	

Refid	Author	Title	Abstract	User	Level	Author	Date	Jurisd	PubType	PubType_comment	Quant-CaseContr	Quant-CrossSect	Quant-Other	Quant-Other_comment	Qual-FocGroup	Qual-SemiStr	Mixed	Aim	Dates	InclExclCriteria	RecruitmentMethods	DataCollection	Participants	Participants_Youth	Age_mean	Gender_Percentage	Pop_Largest	SampleOther	Outcome	Comparator	RiskFactors	Intervention	MainFindings	Notes
			sexuality, contraception, STD and AIDS prevention. In a qualitative research project, Moroccan adolescents were invited to focus groups. The results show the specific influence of family, religion and tradition, the importance of virginity at marriage for girls, and the "almost evidence" of premarital coitus for boys. These adolescents have limited knowledge of contraceptives, STD and AIDS. Some boys pretend to perform safe sex in certain "unfixed" circumstances but show no concern about the possible risks for future virgin spouses. Most of the girls do not consider safe sex before																									38 cases between 5 and 8 (with a greater number for the boys). Forty-four indicated they were not engaged.<br>-All participants could express themselves in Dutch			Moroccan men and boys always have a condom in their pocket. They think it is important to use a condom, in a casual relationship and in contact with a prostitute. They do not use a condom if they think there is no risk, and also with the fact that ‘‘it feels better without a condom’’. Moroccan boys also consider condom use to prevent pregnancy. There are also situations where a condom is forgotten such as in drug use or unexpected sexual relationships. The girls speak of having no sexual relationships before marriage and are therefore not concerned with safe sex.		taboo on homosexuality.<br>-The results show the specific influence of family, religion and tradition. Boys think it is important to use a condom, in a casual relationship and in contact with a prostitute. They do not use a condom if they think there is no risk or are in long term relationships. Most of the girls do not consider safe sex before or after marriage.	

Refid	Author	Title	Abstract	User	Level	Author	Date	Jurisd	PubType	PubType_comment	Quant-CaseContr	Quant-CrossSect	Quant-Other	Quant-Other_comment	Qual-FocGroup	Qual-SemiStr	Mixed	Aim	Dates	InclExclCriteria	RecruitmentMethods	DataCollection	Participants	Participants_Youth	Age_mean	Gender_Percentage	Pop_Largest	SampleOther	Outcome	Comparator	RiskFactors	Intervention	MainFindings	Notes
			or after marriage. There is a taboo on homosexuality.																															
1313	H. El Kazdouh, A. El-Ammari, S. Bouftini, S. El Fakir, Y. El Achhab	Perceptions and intervention preferences of Moroccan adolescents, parents, and teachers regarding risks and protective factors for risky sexual behaviors leading to sexually transmitted infections in adolescents: qualitative findings	BACKGROUND: Sexual choices and practices of adolescents living in conservative societies, including Morocco, can be influenced either positively or negatively by the prevailing contextual and social norms. These norms not only limit the access to reproductive health information and services but also lead to abstinence among devout adolescents. Thus, identifying contextual risks and protective factors of risky sexual behaviors leading to sexually transmitted infections (STIs) in adolescents, as well as exploring	mohammad.alakel	5	El Kazdouh	2019	Taza, Morocco	PubPeer						Qual-FocGroup			To identify the perceptions of adolescents, parents, and teachers regarding factors that affect adolescents' sexual behavior that make them vulnerable to sexually transmitted infections, factors that protect them, and to identify their intervention preferences.	05/ 2016 to 07/2016	Not reported	- Adolescents (28 girls and 28 boys) were randomly recruited from two public middle schools.<br>-Parents (5 females and 21 males) who were selected based on their voluntary participation.<br>-Teachers (5 females and 13 males) in disciplines related to health risk behaviors from the selected schools were randomly invited to participate in the study.	The data were collected via focus group discussions (audio-recorded, transcribed, coded) with three different categories of participants (adolescents, parents, and teachers)	N= 56	N= 56	Adolescent group age range from 14 to 16 years	50% Males, 50% Females	Not reported	-Adolescents, in their last year of middle school<br>-36.4% of adolescents reported having a mother that was illiterate<br>-Qualitative discussions of low income	The adolescent group was selected because their school curriculum includes topics related to health risk behaviors.	Comparing perceptions regarding risky sexual behaviour between adolescents, teachers, and parents.	-Most of the adolescents perceived that they lacked information regarding STIs mode of transmission and prevention methods.<br>-Most participants stated that the lack of sexual health discourse between parents and their sons and daughters could lead to increased adolescent engagement in risky sexual activity.<br>-Participants in all three FGD categories agreed that schools were playing a limited role in raising adolescent awareness about sexuality and STIs and changing their risky behaviors. Participants	N/A	-Allowing parents, teachers, and adolescents to work together could help reduce the socio-cultural and personal barriers that prevent effective communication about sexuality.<br>-Schools can play a vital role in reducing risky sexual behaviors and STI acquisition rates in adolescents by promoting sex education in school curriculum and encouraging adolescents to engage in extracurricular activities and awareness campaigns.<br>-Five overall themes seemed to influence risky sexual behaviors in adolescents: (1) risky sexual	

Refid	Author	Title	Abstract	User	Level	Author	Date	Jurisd	PubType	PubType_comment	Quant-CaseContr	Quant-CrossSect	Quant-Other	Quant-Other_comment	Qual-FocGroup	Qual-SemiStr	Mixed	Aim	Dates	InclExclCriteria	RecruitmentMethods	DataCollection	Participants	Participants_Youth	Age_mean	Gender_Percentage	Pop_Largest	SampleOther	Outcome	Comparator	RiskFactors	Intervention	MainFindings	Notes
			perceptions of adolescents, parents and teachers regarding effective intervention preferences could improve the sexual health of adolescents. METHOD(S): We conducted a qualitative study using focus group discussions (FGDs) based on the socio-ecological model as a theoretical framework. Sample groups of adolescents, parents, and teachers were selected from two public middle schools (disadvantaged and advantaged according to socio-economic level) in Taza city, Morocco, from May to July 2016. Participants were polled on protective factors and perceived																											stated that this was mainly because information and awareness provided at schools are inadequate compared with temptations offered to adolescents on the Internet, the media, and in society<br>-Participants also believed that many risky sexual behaviors have spread and have become acceptable by teenagers despite their contradiction with Moroccan norms and religious instructions.<br>-Most participants agreed on the negative role of television, movies, advertisements, the Internet, and other social media in increasing adolescent sexual risky behaviors by		practices and STIs; (2) the adolescent's social domain; (3) the role of school; (4) media, including internet and social media; and (5) sociocultural norms.	

Refid	Author	Title	Abstract	User	Level	Author	Date	Jurisd	PubType	PubType_comment	Quant-CaseContr	Quant-CrossSect	Quant-Other	Quant-Other_comment	Qual-FocGroup	Qual-SemiStr	Mixed	Aim	Dates	InclExclCriteria	RecruitmentMethods	DataCollection	Participants	Participants_Youth	Age_mean	Gender_Percentage	Pop_Largest	SampleOther	Outcome	Comparator	RiskFactors	Intervention	MainFindings	Notes
			facilitators of risky sexual behaviors leading to sexually transmitted infections (STIs) in adolescents as well on their perception of intervention preferences to reduce the risks. Three sets of data were initially formed, coded, and analyzed using thematic analysis. RESULT(S): Seventeen FGDs were conducted, including 8 groups of adolescents (28 boys and 28 girls, 14-16years old), 5 groups of parents (21 males and 5 females), and 4 groups of teachers (13 males and 5 females). Five overall themes seemed to influence risky sexual behaviors in adolescents: (1)																												showing teenage characters with multiple emotional and sexual relationships.			

Refid	Author	Title	Abstract	User	Level	Author	Date	Jurisd	PubType	PubType_comment	Quant-CaseContr	Quant-CrossSect	Quant-Other	Quant-Other_comment	Qual-FocGroup	Qual-SemiStr	Mixed	Aim	Dates	InclExclCriteria	RecruitmentMethods	DataCollection	Participants	Participants_Youth	Age_mean	Gender_Percentage	Pop_Largest	SampleOther	Outcome	Comparator	RiskFactors	Intervention	MainFindings	Notes
			risky sexual practices and STIs; (2) the adolescent's social domain; (3) the role of school; (4) media, including internet and social media; and (5) socio-cultural norms. Participants also suggested a number of possible interventions to improve the sexual health of adolescents and to reduce the risk of STIs, which could be applied at multiple levels. CONCLUSION(S): Successful intervention programs should target the multifaceted factors affecting the adolescent's sexual behaviors, from the individual to the societal level. Allowing parents, teachers, and adolescents to work together could help reduce the socio-																															

Refid	Author	Title	Abstract	User	Level	Author	Date	Jurisd	PubType	PubType_comment	Quant-CaseContr	Quant-CrossSect	Quant-Other	Quant-Other_comment	Qual-FocGroup	Qual-SemiStr	Mixed	Aim	Dates	InclExclCriteria	RecruitmentMethods	DataCollection	Participants	Participants_Youth	Age_mean	Gender_Percentage	Pop_Largest	SampleOther	Outcome	Comparator	RiskFactors	Intervention	MainFindings	Notes
			cultural and personal barriers that prevent effective communication about sexuality. Furthermore, schools can play a vital role in reducing risky sexual behaviors and STI acquisition rates in adolescents by promoting sex education in school curriculum and encouraging adolescents to engage in extracurricular activities and awareness campaigns.																															
1468	M. Noroozi, S. Ahmadi, B. Armoon, Z. Jorjoran Shushtari, A. Sharhani, E. Ahounbar, S. E. Karimi, A. Rahmani, Y. Mokhayeri, M. Qorbani, M. H. Farhadi, K. Waye, N. Alibeigi, M. R. Bazrafshan	Social determinants associated with risky sexual behaviors among men who inject drugs in Kermanshah, Western Iran	Background: Limited studies exist on risky sexual behaviors and s ociodemographi c variables among people who use drugs. Understanding the social determinants of risky sexual behaviors is important for designing appropriate	mohammad.ala kel	5	Noroozi et al.,	2018	Kermanshah, Iran	PubPeer			Quant-CrossSect						To investigate the social determinants of risky sexual behaviors among people who inject drugs (PWID) in Kermanshah, Iran	2013 to 2014	Interviews were conducted individually with males who were of 18 years of age or older, with histories of injecting illicit drugs at least 1 month prior to the study's onset.<br>Additional inclusion criteria consisted of speaking and understanding	The researchers pooled samples through a snowball sampling method.	Data were collected by holding interviews to acquire information and a structured questionnaire was used.	N= 510	N< 30 = 312	Age of 14 to 78 years.<br>Median=31 years;<br>SD=11.23 years	100% Males	100% IDU	82.5% had a low income (< $150)<br>70% had ever been in jail	-History of substance use (i.e., age of initiation, types of abused drugs during the past 6 months)<br>-Inconsistent condom use with any type of sexual partner, accessing harm reduction services, ever in jail, socioeconomic status: derived	Comparing factors associated with inconsistent condom use among IDU	-Compared to the PWID who did not report inconsistent condom use; participants that were more likely to partake in inconsistent condom use were those of low (AOR = 3.2; P < 0.05)* or moderate (AOR = 2.2; P < 0.05)* socioeconomic status and	N/A	-People who inject drugs who reported having multiple sex partners with inconsistent condom use were more likely to experience poly-drug use, methamphetami ne use, being of low socioeconomic and education level, and refuse to attend harm	

Refid	Author	Title	Abstract	User	Level	Author	Date	Jurisd	PubType	PubType_comment	Quant-CaseContr	Quant-CrossSect	Quant-Other	Quant-Other_comment	Qual-FocGroup	Qual-SemiStr	Mixed	Aim	Dates	InclExclCriteria	RecruitmentMethods	DataCollection	Participants	Participants_Youth	Age_mean	Gender_Percentage	Pop_Largest	SampleOther	Outcome	Comparator	RiskFactors	Intervention	MainFindings	Notes
			HIV/AIDS interventions. Therefore, to illuminate this uncharted area, we aimed to investigate the social determinants of risky sexual behaviors among people who inject drugs (PWID) in Kermanshah, Iran. Method(s): Cross-sectional data were analyzed from the HIV Behavioral Surveillance among PWID, conducted from 2013 to 2014 in Kermanshah, Iran. Additional data were collected by holding interviews to acquire information on s ociodemographic characteristics (i.e., age, educational), history of substance use (i.e., age of initiation, use of specific drug types in the past 6 months),																	Persian language and providing informed consent.									from level of education, employment status, level of income, and housing		reported metha mphetamine use (AOR = 2.8; $P < 0.05$)*<br>-Protective factor: accessing harm reduction services was inversely associated with inconsistent condom use (< 0.05)*.		reduction programs.<br>-The findings illustrate that harm reduction programs (e.g., condom distribution programs) as well as needle and syringe programs are associated with reduced risky sexual behaviors and could be considered a protective factor for inconsistent condom use among those with multiple sex partners)	

Refid	Author	Title	Abstract	User	Level	Author	Date	Jurisd	PubType	PubType_comment	Quant-CaseContr	Quant-CrossSect	Quant-Other	Quant-Other_comment	Qual-FocGroup	Qual-SemiStr	Mixed	Aim	Dates	InclExclCriteria	RecruitmentMethods	DataCollection	Participants	Participants_Youth	Age_mean	Gender_Percentage	Pop_Largest	SampleOther	Outcome	Comparator	RiskFactors	Intervention	MainFindings	Notes
			sexual behaviors including consistent condom use (yes/no), and type of sexual partners (main, casual, exchange). We analyzed variables individually through bivariate analysis and subsequently ran significant variables (P < 0.2) in a multivariate regression model. P < 0.05 was considered statistically significant. Descriptive statistics and logistic regression were conducted in Stata 11. Result(s): Participants were between the age of 14 and 78 years (median = 31 years; SD = 11.23 years). Compared to PWIDs who reported no sexual risk																															

Refid	Author	Title	Abstract	User	Level	Author	Date	Jurisd	PubType	PubType_comment	Quant-CaseContr	Quant-CrossSect	Quant-Other	Quant-Other_comment	Qual-FocGroup	Qual-SemiStr	Mixed	Aim	Dates	InclExclCriteria	RecruitmentMethods	DataCollection	Participants	Participants_Youth	Age_mean	Gender_Percentage	Pop_Largest	SampleOther	Outcome	Comparator	RiskFactors	Intervention	MainFindings	Notes
			behavior, participants that were more likely to partake in sexual risk behaviors were those of low (adjusted odds ratio [AOR] = 3.2, P < 0.05) or moderate (AOR = 2.2, P < 0.05) socioeconomic status and meth amphetamine use (AOR = 2.8, P < 0.05). Conclusion(s): A key finding of this study is that PWID who reported having multiple sex partners with inconsistent condom use were more likely to experience poly-drug use, methamphetamine use, being of low socioeconomic and education level, and refuse to attend harm reduction programs. Copyright © 2018, © 2018 Taylor & Francis Group, LLC.																																

Refid	Author	Title	Abstract	User	Level	Author	Date	Jurisd	PubType	PubType_comment	Quant-CaseContr	Quant-CrossSect	Quant-Other	Quant-Other_comment	Qual-FocGroup	Qual-SemiStr	Mixed
1490	B. Armoon, M. Noroozi, Z. Jorjoran Shushtari, A. Sharhani, E. Ahounbar, S. Karimi, S. Ahmadi, A. Farhoudian, A. Rahmani, M. Abbasi, B. D. L. Marshal, O. Rezaei, F. Rezaei, H. Sharifi, M. Najafi, A. Bazrafshan, M. H. Farhadi	Factors associated with HIV risk perception among people who inject drugs: Findings from a cross-sectional behavioral survey in Kermanshah, Iran	Background: In Iran there are limited data regarding HIV risk perceptions among people who inject drugs (PWID). The objective of this study was to explore HIV risk perception and to examine factors associated with the perception of HIV risk among PWID in Iran. Methods: We surveyed 433 PWID in Kermanshah concerning demographic characteristics, sexual risk behaviors, HIV risk perception, and drug-related risk behaviors in the month prior to the study. Two classes of HIV risk perception (high vs. low) were identified. We used logistic regression to identify factors associated with high HIV risk perception. Result: Of 433	mohammad.alakel	5	Armoon et al.,	2018	Kermanshah, Iran	PubPeer			Quant-CrossSect					

Refid	Aim	Dates	InclExclCriteria	RecruitmentMethods	DataCollection	Participants	Participants_Youth	Age_mean	Gender_Percentage	Pop_Largest	SampleOther	Outcome	Comparator	RiskFactors	Intervention	MainFindings	Notes
1490	To explore HIV risk perception and to examine factors associated with the perception of HIV risk among people who inject drugs in Iran.	09/2014 to 12/2014	-Participants were required to be over 18 years old and to have injected illicit drugs at least once in the past month, and the ability to speak and comprehend Farsi enough to respond to survey questions.	Using snowball sampling.	Face-to-face interviews were conducted using a structured questionnaire.	N=433	N= 255 (age<30)	The mean age ± standard deviation (SD) was 33.5 ± 7.6 (interquartile range (IQR), 25.6–42.4) years	100% males	100% people who inject drugs (PWID)	-70% belong to low socioeconomic status<br>-49% unemployed	-Examined injecting behavior, sexual behavior, exposure to harm reduction, and HIV prevention interventions.<br>-The injection behaviors considered: duration of injection drug use, frequency of injection in the past 1 month, sharing (borrowing or lending) syringes/ needles and cookers, and reuse of syringes (yes/no) in the past 1 year.	-The study compared risk perception (low vs high) by socio-demographic characteristics in PWID .	-The PWID with high HIV risk perception compared with 'low perception' were more likely to be unemployed ($p < 0.05$)*, homeless ($p < 0.05$)*, and in low socioeconomic status ($p < 0.05$)*, use methamphetamine ($p < 0.05$)*, started drug use under the age of 22 ($p < 0.05$)*, and use multiple drugs at the same time ($p < 0.05$)*.<br>-Those with low (AOR = 3.4, $p < 0.05$)* or moderate (AOR = 1.8, $p < 0.05$)* socioeconomic status, those who started drug use at any age under 22 years old (AOR = 2.2, $p < 0.05$)*, PWID who used methamphetamine (AOR = 2.8, $p < 0.05$)*, and those who used multiple drugs at	N/A	This study found that socioeconomic status, initiating drug use at younger age, using methamphetamine, polydrug use, and needle- and syringe-exchange program utilization were associated with higher HIV risk perception among PWID in Iran.	

Refid	Author	Title	Abstract	User	Level	Author	Date	Jurisd	PubType	PubType_comment	Quant-CaseContr	Quant-CrossSect	Quant-Other	Quant-Other_comment	Qual-FocGroup	Qual-SemiStr	Mixed	Aim	Dates	InclExclCriteria	RecruitmentMethods	DataCollection	Participants	Participants_Youth	Age_mean	Gender_Percentage	Pop_Largest	SampleOther	Outcome	Comparator	RiskFactors	Intervention	MainFindings	Notes
			PWID who participated in this study, 36% (95% confidence interval; CI95%: 25.3%, 42.2%) of participants reported high HIV risk perception. Met hamphetamine use (adjusted odds ration; AOR = 3.7, $p < 0.05$), or use of multiple drugs at the same time (AOR = 1.7, $p < 0.05$) was associated with higher HIV risk perception. Moreover, PWID who were NSP users had 2.8 times the odds of high risk perception compared with non-Needle and Syringe Program (NSP) users (AOR = 2.8). Conclusion: This study implies that initiating drug use at a younger age, using methamp hetamine, polydrug use,																												the same time (AOR = 2.1, $p < 0.05$)* were more likely to report high HIV risk perception. -PWID that were syringe program users had 2.8 times the odds of high risk perception compared with non-Needle and Syringe Program users.			

Refid	Author	Title	Abstract	User	Level	Author	Date	Jurisd	PubType	PubType_comment	Quant-CaseContr	Quant-CrossSect	Quant-Other	Quant-Other_comment	Qual-FocGroup	Qual-SemiStr	Mixed	Aim	Dates	InclExclCriteria	RecruitmentMethods	DataCollection	Participants	Participants_Youth	Age_mean	Gender_Percentage	Pop_Largest	SampleOther	Outcome	Comparator	RiskFactors	Intervention	MainFindings	Notes
			and needle- and syringe-exchange program utilization were predictors of higher HIV risk perception among PWID in Iran. Copyright © 2017 Taylor & Francis Group, LLC.																															
1575	F. El Kak, N. El Salibi, L. Ghandour	Sexual communication and risky sexual practices and perceptions among university students from lebanon	Aim: Investigate the association between youth sexual practices and perceptions and their sexual communication with partners, parents/guardians and peers. Methods: Cross-sectional online survey data on 2180 undergraduate and graduate students (18-30 years) attending a large private university in Lebanon. Statistically significant (p-value<0.05) sex-adjusted findings are reported. Results: 81% talked about sex with their close	mohammad.alakel	5	El Kak et al.,	2017	Beirut, Lebanon	PubPeer			Quant-CrossSect						To investigate the association between youth sexual practices and perceptions and their sexual communication with partners, parents/guardians and peers.	Not reported	Not reported	Not reported	Not reported	N=2180	N=2180	Range= 18-30	Not reported	Not reported	All attending a private university in Lebanon.	Not reported	Not reported	-Students who felt comfortable communicating with peers were more likely to engage in sexual intercourse with 3 or more partners, engage in first oral sex with an unfamiliar partner and drink/use drugs at sexual debut. -Students who discussed sexual activity preferences with partners were more likely to perceive condoms as effective at preventing pregnancy, HIV/AIDS, and other STIs, and were more likely	N/A	-Students who communicated with peers about sexual preferences/matters were more likely to engage in risky sexual practices but were also more well-informed about sexual health matters. Further studies must investigate the content/frequency of sexual communication to better guide interventions promoting positive sexual communication, and sexual assertiveness in youth. -Students who communicated with partners	Conference abstract published in peer reviewed journal

Refid	Author	Title	Abstract	User	Level	Author	Date	Jurisd	PubType	PubType_comment	Quant-CaseContr	Quant-CrossSect	Quant-Other	Quant-Other_comment	Qual-FocGroup	Qual-SemiStr	Mixed	Aim	Dates	InclExclCriteria	RecruitmentMethods	DataCollection	Participants	Participants_Youth	Age_mean	Gender_Percentage	Pop_Largest	SampleOther	Outcome	Comparator	RiskFactors	Intervention	MainFindings	Notes
			friends, 57% with their partners, 31% with their mother and only 23% with their father. Students who felt comfortable communicating with peers were more likely to engage in sexual intercourse with 3 or more partners, engage in first oral sex with an unfamiliar partner and drink/use drugs at sexual debut. Students who discussed sexual activity preferences with partners were more likely to perceive condoms as effective at preventing pregnancy, HIV/AIDS, and other sexually transmitted infections, and were more likely to believe that birth control pills were effective at preventing pregnancy. Not																											to believe that birth control pills were effective at preventing pregnancy.<br>-Not communicating with partners was associated with increased odds of not knowing about the effectiveness of condoms at preventing pregnancy and misperceiving that birth control pills are effective at preventing HIV/AIDS. Students who didn't communicate with a partner, close friends, or parents were more likely to admit never hearing of emergency contraceptive pills or unsure of their use.		were more likely to think condoms are effective against STIs and HIV, not communicating with their partners were associated with an increased odds of not knowing about the effectiveness of condoms.	

Refid	Author	Title	Abstract	User	Level	Author	Date	Jurisd	PubType	PubType_comment	Quant-CaseContr	Quant-CrossSect	Quant-Other	Quant-Other_comment	Qual-FocGroup	Qual-SemiStr	Mixed	Aim	Dates	InclExclCriteria	RecruitmentMethods	DataCollection	Participants	Participants_Youth	Age_mean	Gender_Percentage	Pop_Largest	SampleOther	Outcome	Comparator	RiskFactors	Intervention	MainFindings	Notes
			communicating with partners was associated with increased odds of not knowing about the effectiveness of condoms at preventing pregnancy and misperceiving that birth control pills are effective at preventing HIV/AIDS. Besides, students who didn't communicate with a partner, close friends, or parents were more likely to admit never hearing of emergency contraceptive pills or unsure of their use. Conclusion: Students who communicated with partners about sexual pr eferences/matte rs were more likely to engage in risky sexual practices but were also more well-informed about sexual																															

Refid	Author	Title	Abstract	User	Level	Author	Date	Jurisd	PubType	PubType_comment	Quant-CaseContr	Quant-CrossSect	Quant-Other	Quant-Other_comment	Qual-FocGroup	Qual-SemiStr	Mixed	Aim	Dates	InclExclCriteria	RecruitmentMethods	DataCollection	Participants	Participants_Youth	Age_mean	Gender_Percentage	Pop_Largest	SampleOther	Outcome	Comparator	RiskFactors	Intervention	MainFindings	Notes
			health matters. Further studies must investigate the content/frequency of sexual communication to better guide interventions promoting positive sexual communication, and sexual asser-tiveness in youth.																															
1822	L. G. Johnston, K. Alami, M. H. E. Rhilani, M. Karkouri, O. Mellouk, A. Abadie, N. Rafif, L. Ouarsas, A. Bennani, B. E. Omari	HIV, syphilis and sexual risk behaviours among men who have sex with men in Agadir and Marrakesh, Morocco	Objective To collect baseline measurements of HIV and syphilis prevalence and sexual risk behaviours among men who have sex with men (MSM) in Agadir and Marrakech, Morocco, and provide strategic information to improve outreach programmes. Methods and Design Respon dent-driven sampling was used to recruit men who reported having anal sex with another man in the last 6	mohammad.alakel	5	Johnston et al.,	2013	Agadir and Marrakesh, Morocco	PubPeer			Quant-CrossSect						To collect baseline measurements of HIV and syphilis prevalence and sexual risk behaviours among men who have sex with men (MSM) in Agadir and Marrakech, Morocco, and provide strategic information to improve outreach programmes.	2010-2011	Men who reported having anal sex with another man in the last 6 months, aged 18 years and older and living in either Agadir or Marrakesh for the past 6 months, regardless of nationality.	MSM were sampled using r espondent-driven sampling, a peer-to-peer recruitment method. Respondents were compensated for completing the survey and enrolling others.	Eligible men completed a face-to-face interview in Arabic, and provided blood specimens for HIV and syphilis testing from a biobehavioural survey	Agadir (N=323)<br>Marrakech (N=346)<br>Total N= 669	N = 235 in Agadir for age <= 24<br>N= 228 in Marrakech for age <=24<br>N total <= 24 is 463	Not reported	100% Males	100% MSM (90% identify as gay/ bisexual/transsexual)	52.6% unemployed, 95.3% single	-HIV and syphilis prevalence (via blood testing) and sexual risk behaviours (ex. Paid money or goods for sex (past 6 months), received money or goods for sex in past 6 months, Condom use—last anal sex with male commercial sex partner, Anal sex—regular male sex partners (past 6 months)).	-Risk factors among MSM in Agadir compared to MSM in Marrakech.	-76% ever had sex with a woman<br>-67% reported receiving money/goods for sex<br>- 54.8% of male participants had anal sex with an occasional male sex partner within the past 6 months<br>-35% did not use condom at last anal sex with male commercial sex partner	N/A	-3% of men who have sex with men (MSM) in Marrakesh and 6% in Agadir are HIV positive,<br>-MSM have reported multiple male anal sex partner types and inconsistent condom use.<br>MSM are having unprotected sex with women,<br>MSM reported receiving money or goods for sex which may be a means of earning extra income and/or avoiding being ‘homosexual’ or ‘gay’.	

Refid	Author	Title	Abstract	User	Level	Author	Date	Jurisd	PubType	PubType_comment	Quant-CaseContr	Quant-CrossSect	Quant-Other	Quant-Other_comment	Qual-FocGroup	Qual-SemiStr	Mixed	Aim	Dates	InclExclCriteria	RecruitmentMethods	DataCollection	Participants	Participants_Youth	Age_mean	Gender_Percentage	Pop_Largest	SampleOther	Outcome	Comparator	RiskFactors	Intervention	MainFindings	Notes
			months, aged 18 years and older and living in either Agadir or Marrakech for the past 6 months, regardless of nationality. Data were analysed with the multiplicity estimator using respondent-driven sampling analysis tool V.6.0. Results 323 MSM in Agadir and 346 in Marrakech were recruited into the survey. Most MSM in both cities reported being < 25 years, being unemployed, bisexual and in a couple with both a man and a woman. Most reported selling sex and having sex with women. HIV prevalence was 5.6% in Agadir and 2.8% in Marrakesh; syphilis was 7.0% in Agadir and 10.8% in Marrakesh. Among MSM																															

Refid	Author	Title	Abstract	User	Level	Author	Date	Jurisd	PubType	PubType_comment	Quant-CaseContr	Quant-CrossSect	Quant-Other	Quant-Other_comment	Qual-FocGroup	Qual-SemiStr	Mixed	Aim	Dates	InclExclCriteria	RecruitmentMethods	DataCollection	Participants	Participants_Youth	Age_mean	Gender_Percentage	Pop_Largest	SampleOther	Outcome	Comparator	RiskFactors	Intervention	MainFindings	Notes
			who tested positive for HIV, 31.6% in Agadir and 56.4% in Marrakesh were co-infected with syphilis. Conclusions HIV and syphilis findings coupled with high risk activities indicate the need for expanding programmes targeting MSM throughout Morocco. Selling sex and sex with women may be a strategy to cope with extreme stigma towards MSM. Criminalisation and discrimination of MSM in Morocco underscores the urgent need for longterm and sustainable risk reduction through legal reforms and promotion and protection of human rights.																															
1989	A. Sayarifard, A. A. Kolahi, M.	Frequency of unprotected	Considering the lack of	mohammad.alakel	5	Sayarifard et al.,	2011	Tehran, Iran	PubOther	conference abstract		Quant-CrossSect						To determine the frequency of	2009	Not reported	The sampling was a	Data from interviews were	N= 128	Not reported	Mean age of participants was	100% females	100% female sex workers	Not reported	Outcomes: frequency of	N/A	-51 (39.8%) of the female sex	N/A	Lack of using condoms during	Conference abstract in peer

Refid	Author	Title	Abstract	User	Level	Author	Date	Jurisd	PubType	PubType_comment	Quant-CaseContr	Quant-CrossSect	Quant-Other	Quant-Other_comment	Qual-FocGroup	Qual-SemiStr	Mixed	Aim	Dates	InclExclCriteria	RecruitmentMethods	DataCollection	Participants	Participants_Youth	Age_mean	Gender_Percentage	Pop_Largest	SampleOther	Outcome	Comparator	RiskFactors	Intervention	MainFindings	Notes
	A. Hajjar Hamedani	sexual behaviors regarding HIV/AIDS transmission in at-risk women	information about the frequency of anal intercourse, this study aimed to determine the frequency of anal intercourse and the use of condom amongst a sample of female sex workers in Tehran/Iran in 2009.We conducted a cross-sectional study and obtained data from 128 female sex workers in Tehran. The sampling was a combination of available samples from who worked in the streets, visitor passes to Drop-in Centers and those who were introduced by young men and introduced by female sex workers through respondent driven sampling. Data from interviews were collected by trained															anal intercourse and the use of condoms amongst a sample of female sex workers in Tehran/Iran in 2009			combination of available samples from who worked in the streets, visitor passes to Drop-in Centers and those who were introduced by young men and introduced by female sex workers respondent driven sampling.	collected by trained personnel			26.8, SD 6.2, Range (16-45) years				anal intercourse and the use of condoms		workers in the study said they never had anal intercourse -from the total of 77 participants who had anal intercourse, only 22 (28.6%) always or sometimes used condom and 71.4% never used condom.		anal intercourse was more common among elder or addicted female sex workers and those who worked in south of the city and also those who used a type of contraceptive.	reviewed journal

Refid	Author	Title	Abstract	User	Level	Author	Date	Jurisd	PubType	PubType_comment	Quant-CaseContr	Quant-CrossSect	Quant-Other	Quant-Other_comment	Qual-FocGroup	Qual-SemiStr	Mixed	Aim	Dates	InclExclCriteria	RecruitmentMethods	DataCollection	Participants	Participants_Youth	Age_mean	Gender_Percentage	Pop_Largest	SampleOther	Outcome	Comparator	RiskFactors	Intervention	MainFindings	Notes
			personnel. Mean age of participants was 26.8 +/- 6.2(16-45) years. 51 (39.8%) of the female sex workers in the study said they never had anal intercourse, 13 (10.1%) reported sometimes, only 8 (6.3%) of whom always used condom and 56 (43.8%) never used condom. in other words, from the total of 77 participants who had anal intercourse, only 22 (28.6%) always or sometimes used condom and 71.4% never used condom. Lack of using condoms during anal intercourses was more common among elder or addicted female sex workers and those who worked in south of the city and																															

Refid	Author	Title	Abstract	User	Level	Author	Date	Jurisd	PubType	PubType_comment	Quant-CaseContr	Quant-CrossSect	Quant-Other	Quant-Other_comment	Qual-FocGroup	Qual-SemiStr	Mixed	Aim	Dates	InclExclCriteria	RecruitmentMethods	DataCollection	Participants	Participants_Youth	Age_mean	Gender_Percentage	Pop_Largest	SampleOther	Outcome	Comparator	RiskFactors	Intervention	MainFindings	Notes
			also those who used a type of contraceptive. This study showed that the behavior of female sex worker in Iran towards preventing the transmission of HIV/AIDS is not satisfying at all. Continuing this situation puts themselves and the society in higher risk of damage.																															
2051	Z. Mahfoud, R. Afifi, S. Ramia, D. E. Khoury, K. Kassak, F. E. Barbir, M. Ghanem, M. El-Nakib, J. Dejong	HIV/AIDS among female sex workers, injecting drug users and men who have sex with men in Lebanon: Results of the first biobehavioral surveys	Objectives: To measure HIV prevalence and associated risk factors among female sex workers, injecting drug users (IDUs) and men who have sex with men (MSM) in Lebanon and the prevalence of hepatitis B virus and hepatitis C virus among IDUs. Methods and Design: A cross-sectional survey of 135 female sex workers, 81 IDUs and 101	mohammad.alakel	5	Mahfoud et al.,	2010	Beirut, Lebanon	PubPeer			Quant-CrossSect						To measure HIV prevalence and associated risk factors among female sex workers, injecting drug users and men who have sex with men in Lebanon and the prevalence of hepatitis B virus and hepatitis C virus among injecting drug users.	08/2007 and 07/2008	-Participants needed to be residents of Lebanon, at least 16 years old and engaged in an HIV risk behavior within the past 12 months (i.e., women who had sold sex, men who had injected drugs or men who had sex with a man).<br>-Excluded: participants not capable of understanding the informed consent in Arabic or	Given the hidden nature of the risk groups under study, respondent-driven sampling (a type of snowball sampling) was chosen.	Interviews using a biobehavioral survey were conducted at the NGO sites or at an agreed mutually convenient site by interviewers from the NGO.	N= 317	N= 143 for age (16-24)	Not reported range (16 to >25)	57% men (all IDUs were male)	43% FSW, 32% MSM, 26% IDU	85% IDUs and 19% FSW were ever jailed	Risk behaviors (ex. number of clients last month, ever sold sex, injected in past month, condom use with non-regular noncommercial male sex partners in last month), the sociodemographic background of participants, prior testing for HIV and knowledge and attitudes about HIV	Comparing knowledge about and attitudes toward HIV across FSW, IDU, and MSM	- 63% of the FSWs stated they were under 18 years of age at first sexual intercourse.<br>-96% FSW had five or more clients in the last month.<br><br>-The majority of IDUs (52%) had injected five or more times in the last month.<br>-Only 24% stated that they used new needles every time they inject, and 21% had shared a needle the last time	N/A	-HIV prevalence was 3.7% (n=1) among MSM but no HIV cases were detected among female sex workers or IDUs.<br>-Among IDUs, prevalence of hepatitis C virus antibody was 51%.<br>-75% of MSM had nonregular male sexual partners during the last year but only 39% reported using a condom every time.<br>-36% of MSM and 12% of	

Refid	Author	Title	Abstract	User	Level	Author	Date	Jurisd	PubType	PubType_comment	Quant-CaseContr	Quant-CrossSect	Quant-Other	Quant-Other_comment	Qual-FocGroup	Qual-SemiStr	Mixed	Aim	Dates	InclExclCriteria	RecruitmentMethods	DataCollection	Participants	Participants_Youth	Age_mean	Gender_Percentage	Pop_Largest	SampleOther	Outcome	Comparator	RiskFactors	Intervention	MainFindings	Notes
			MSM was recruited using r espondent-driven sampling. A structured interview was conducted by members of non governmental organizations working with these populations and blood was collected for serological testing. Results: HIV prevalence was 3.7% among MSM but no HIV cases were detected among female sex workers or IDUs. Among IDUs, prevalence of hepatitis C virus antibody was 51% and prevalence of hepatitis B virus surface antigen was 5%. Three-quarters of MSM had nonregular male sexual partners during the last year but only 39% reported using a condom every time. There was																	English.											they injected.<br>-25% of IDUs stated that they were under 20 years of age when they had first injected drugs.<br>- 43% IDUs used a condom the last time they had sex with a regular non-commercial sex partner<br><br>-52% of MSM stated they were under 18 years of age at first sexual intercourse.<br>-Overall 63% of MSM reported having had sex with a man they met over internet.<br>-About three quarters (73%) of the MSM had at least one nonregular noncommercial sex partner in the last year<br>-63% of MSMused a condom every time they had sex with a regular noncommercial partner, but only		IDUs reported that they had sold sex<br>-Previous testing for HIV was lowest among MSM (at 22%) despite their having the highest level both of knowledge about HIV and of perception of being at risk of HIV infection (67%).<br>-88% of IDUs and 86% of FSWs had taken the HIV test as opposed to only 30% of MSM. A rationale is that IDUs and FSWs had taken the test in the past as part of a mandatory requirement.	

Refid	Author	Title	Abstract	User	Level	Author	Date	Jurisd	PubType	PubType_comment	Quant-CaseContr	Quant-CrossSect	Quant-Other	Quant-Other_comment	Qual-FocGroup	Qual-SemiStr	Mixed	Aim	Dates	InclExclCriteria	RecruitmentMethods	DataCollection	Participants	Participants_Youth	Age_mean	Gender_Percentage	Pop_Largest	SampleOther	Outcome	Comparator	RiskFactors	Intervention	MainFindings	Notes
			evidence of overlapping HIV risk: 36% of MSM and 12% of IDUs reported that they had sold sex. Previous testing for HIV was lowest among MSM (at 22%) despite their having the highest level both of knowledge about HIV and of perception of being at risk of HIV infection (67%). Conclusion: Prevention efforts at greater scale are needed to reach these at-risk populations in Lebanon. These should target MSM in particular, including access to HIV testing, but will need to address and overcome stigma. For IDUs, surveillance and prevention efforts should integrate both hepatitis C virus																												39% did so every time they had sex with non-regular noncommercial sex partners in the last month.<br><br>-91% of the FSWs stated that they used condoms every time they had sex with their regular male clients yet only 48% stated that they had used condoms with their noncommercial partner the last time they had sex.			

Refid	Author	Title	Abstract	User	Level	Author	Date	Jurisd	PubType	PubType_comment	Quant-CaseContr	Quant-CrossSect	Quant-Other	Quant-Other_comment	Qual-FocGroup	Qual-SemiStr	Mixed	Aim	Dates	InclExclCriteria	RecruitmentMethods	DataCollection	Participants	Participants_Youth	Age_mean	Gender_Percentage	Pop_Largest	SampleOther	Outcome	Comparator	RiskFactors	Intervention	MainFindings	Notes
			and HIV. © 2010 Wolters Kluwer Health Lippincott Williams & Wilkins.																															
2054	S. Shayesteh Khou, N. Moshtagh Bidokhti, A. Pourshahbaz	Effectiveness of project RESPECT in reducing sexual risk behaviors of male-to-female transgender patients	Objective: The purpose of the present study was to determine the efficacy of project RESPECT in reducing sexual risk behaviours of male to female transgenders. Design and Method: This study was an interventional, q uasiexperimental and case-control. 30 transgenders, male to female, which had sexual risk behaviours were selected and divided into experimental and control groups. Transgenders in experimental group received 4 - session intervention conducted one session per	mohammad.alakel	5	Shayestehkhou et al.,	2013	Tehran, Iran	PubPeer		Quant-CaseContr							To determine the effectiveness of Project RESPECT (consists of counselling sessions) in reducing sexual risk behaviors of transgender women in Tehran.	Not reported	General eligibility: (1) To be identifying as a transgender woman; (2) 18 to 35 years old; (3) Having sexual behaviors during the past month; and (4) Having a history of sexual risk behaviors such as multiple sexual partners, irregular condom use and drug use during intercourse.	Participants were recruited through two private and public clinics of sexual disorders in Tehran.	The questionnaires collected demographic data and evaluated sexual risk behaviors.	N= 30	100% age: 18-35	Mean age is 23.67 (experimental) and 24.47 (control) and range: 18-35	100% transgender women	100% transgender women	Not reported	-The questionnaire indicated occurrence, frequency and number of sexual partners, specific sexual acts with each partner, the use of condoms during each of sexual acts and the use of drugs during sexual behaviors. Sexual risk behaviors were given scores from 1 to 4 with respect to their riskiness.	-Compared risk behaviours pre and post intervention among experimental and control groups.	-Among the 30 MTF transgender patients, no one had a stable partner during last month. Furthermore, 60% had both casual and stable partners. -Participants who had both stable and casual partners, no one had used condoms in sexual behaviors with stable partners. -However, at pretest 33.34% of the experimental group and 13.34% of control group who had both stable and casual partners had used condoms regularly during intercourse with their casual partners. In addition,	-Counselling intervention: focuses on increasing consistent condom use for vaginal and anal sex with all partners. -Session 1 lasted 15-20 minutes and focused on a personalized assessment of risk and establishment of risk reduction plan. The remaining sessions lasted 60 minutes. The purpose of session 2 was to change self-efficacy of condom use. Session 3 involved HIV testing and identifying situational barriers to condom use.	Project RESPECT is more effective in reducing higher risk sexual risk behaviors than frequency of sexual risk behaviors among male to female transgender patients.	

Refid	Author	Title	Abstract	User	Level	Author	Date	Jurisd	PubType	PubType_comment	Quant-CaseContr	Quant-CrossSect	Quant-Other	Quant-Other_comment	Qual-FocGroup	Qual-SemiStr	Mixed	Aim	Dates	InclExclCriteria	RecruitmentMethods	DataCollection	Participants	Participants_Youth	Age_mean	Gender_Percentage	Pop_Largest	SampleOther	Outcome	Comparator	RiskFactors	Intervention	MainFindings	Notes
			week and individually, but control group received no intervention. The instruments of this research were Demographic and Sexual Risk Questionnaires. Data were analyzed by independent t-test, chi square and percent differences. Results: Although frequency of sexual risk behaviours didn't changed significantly in experimental group, higher sexual risk behaviors were reduced significantly (p=0.01). Conclusion: Project RESPECT was more effective in reducing higher sexual risk behaviors than frequency of sexual risk behaviours of male to female transgenders.																												13.33% of experimental group and 20% of control group consumed drugs while they had sexual behaviors.<br>-A 13.58% reduction in frequency of sexual risk behaviors in experimental group at posttest (not significantly different from pretest).<br>-Although frequency of sexual risk behaviors did not change significantly in experimental group, higher sexual risk behaviors were reduced significantly (P?0.01)*.			

Refid	Author	Title	Abstract	User	Level	Author	Date	Jurisd	PubType	PubType_comment	Quant-CaseContr	Quant-CrossSect	Quant-Other	Quant-Other_comment	Qual-FocGroup	Qual-SemiStr	Mixed	Aim	Dates	InclExclCriteria	RecruitmentMethods	DataCollection	Participants	Participants_Youth	Age_mean	Gender_Percentage	Pop_Largest	SampleOther	Outcome	Comparator	RiskFactors	Intervention	MainFindings	Notes
4013	M. Noroozi, B. D. L. Marshall, A. Noroozi, B. Armoon, H. Sharifi, M. Qorbani, M. Abbasi, M. R. Bazrafshan	Effect of Alcohol Use on Injection and Sexual Behavior among People Who Inject Drugs in Tehran, Iran: A Coarsened Exact Matching Approach	Background: Many studies have recognized the importance of sexual and injection risk behaviors in HIV transmission among people who inject drugs (PWID). We aimed to examine effect of alcohol use on sexual and injection risky behavior using coarsened exact matching (CEM) approach among PWID in Tehran, Iran. Study design: A cross-sectional study. Methods: This study was conducted from Mar to Aug 2016 in Tehran, Iran. PWID were recruited by a convenience and snowball sampling from five of Drop-in Centers (DICs) in Tehran. We assessed three outcomes of interest, all treated as binary variables, including distributive and	mohammad.alakel	5	Noroozi et al.,	2018	Tehran, Iran	PubPeer			Quant-CrossSect						To examine effect of alcohol use on sexual and injection risky behavior using coarsened exact matching approach among injection drug users in Tehran, Iran.	03/2016 to 08/2016	Age 18 yr or more, IDU at least once during the last month, and to be able to provide informed consent.	PWID were recruited by a convenience and snowball sampling from five of Drop-in Centers in Tehran	Trained interviewers administered the questionnaire	N=550	N= 455 (Age <30)	The matched sample (n=400) had a mean age ±SD of 33.9±8.9 (range: 18-60)	100% Males	100% PWID	.-80% of participants low income (<$150 US/month)<br>-50% of the sample reported alcohol use during month prior to the interview<br>-30% of participants had less than high school education	-Receptive syringe sharing (obtaining and using a syringe after being used by someone else), distributive syringe sharing (giving someone else a syringe after you have used it), inconsistent condom use in the last 30 days.	Compared the effect of alcohol use on injecting and sexually risk behaviours in matched and unmatched samples of PWID.	In the matched samples, last month drinkers were more likely to report receptive sharing (OR=2.12 95%; P=0.022)*,<br>as compared to abstainer participants. Distributive sharing in last 30 d, was also significantly higher among last month drinkers group (OR=2.72; P=0.011)*, compared last month abstainers group. Alcohol users had higher odds of alcohol use and inconsistent condom use (OR=4.21; P=0.013)*.	N/A	Alcohol use in the last 30 days was associated with receptive sharing, distributive sharing and an increase in inconsistent condom use with any kind of sexual partners among PWID.	

Refid	Author	Title	Abstract	User	Level	Author	Date	Jurisd	PubType	PubType_comment	Quant-CaseContr	Quant-CrossSect	Quant-Other	Quant-Other_comment	Qual-FocGroup	Qual-SemiStr	Mixed	Aim	Dates	InclExclCriteria	RecruitmentMethods	DataCollection	Participants	Participants_Youth	Age_mean	Gender_Percentage	Pop_Largest	SampleOther	Outcome	Comparator	RiskFactors	Intervention	MainFindings	Notes
			receptive sharing and inconsistent condom use with any type of sexual partner. We applied coarsened exact matching (CEM) to estimate the independent effect of alcohol use on injection and sexual risk behaviours. All data analysis was performed using Stata v.11. Results: Overall, 550 PWID (all male) were enrolled The prevalence of receptive sharing, distributive sharing, and inconsistent condom use was 32%, 15% and 55%, respectively. In the matched samples, last month drinkers were more likely to report receptive sharing (OR=2.12 95% CI:1.31, 3.81, P=0.022), as compared to abstainer																															

Refid	Author	Title	Abstract	User	Level	Author	Date	Jurisd	PubType	PubType_comment	Quant-CaseContr	Quant-CrossSect	Quant-Other	Quant-Other_comment	Qual-FocGroup	Qual-SemiStr	Mixed	Aim	Dates	InclExclCriteria	RecruitmentMethods	DataCollection	Participants	Participants_Youth	Age_mean	Gender_Percentage	Pop_Largest	SampleOther	Outcome	Comparator	RiskFactors	Intervention	MainFindings	Notes
			participants Distributive sharing in last 30 d, was also significantly higher among last month drinkers group (OR=2.72 95% CI 1.72, 4.21, P=0.011), compared last month abstainers group. Finally, there was a statistically significant relationship between alcohol use and inconsistent condom use (OR=4.21 95% CI: 2.71, 7.52, P=0.013). Conclusions: The findings emphasize importance of addressing alcohol use in risk reduction interventions for Iranian PWID with alcohol use.																															
4474	G. J. Wagner, M. Hoover, H. Green, J. Tohme, J. Mokhbat	SOCIAL, RELATIONAL, AND NETWORK DE TERMINANTS OF	Objectives: We examined the social, relational and network determinants of condom use	nicole.andruszkiewicz	5	Wagner et al.,	2015	Beirut, Lebanon	PubPeer			Quant-CrossSect						To examine the social, relational and network determinants of condom use and HIV testing	05/2012 to 12/2012	Being biologically male and male-identified, age 18 years or older, being	Respondent-driven sampling was used.	A survey was administered in English or Arabic with com puter-assisted interview	N= 213	47% < 25 years (n=100)	Mean age was 26.2 years (SD 6.3 years; range 18–52 years;	100% Males	100% MSM	-72% employed -65% had some college education	-Network centralization: a measure of the degree to which one or a few individuals	Compared odds of taking part in any unprotected anal intercourse and HIV Testing across	-Independent correlates of having ever been tested for HIV were lower condom use self-	N/A	In multivariate analysis, being in a relationship was associated with unprotected anal intercourse	Same sample as study ref id 193 (some different findings)

Refid	Author	Title	Abstract	User	Level	Author	Date	Jurisd	PubType	PubType_comment	Quant-CaseContr	Quant-CrossSect	Quant-Other	Quant-Other_comment	Qual-FocGroup	Qual-SemiStr	Mixed	Aim	Dates	InclExclCriteria	RecruitmentMethods	DataCollection	Participants	Participants_Youth	Age_mean	Gender_Percentage	Pop_Largest	SampleOther	Outcome	Comparator	RiskFactors	Intervention	MainFindings	Notes
		UNPROTECTED ANAL SEX AND HIV TESTING AMONG MEN WHO HAVE SEX WITH MEN IN BEIRUT, LEBANON	and HIV testing among men who have sex with men (MSM) in Beirut. Methods: Two-hundred thirteen men were recruited via respondent driven sampling and administered a survey. Results: Sixty-four percent reported unprotected anal intercourse (UAI), including 23% who had UAI with unknown HIV status partners (UAIU); 62% of participants had tested for HIV. In multivariate analysis, being in a relationship was associated with UAI and HIV testing; lower condom self-efficacy was associated with UAIU and HIV testing; gay discrimination was associated with UAIU; MSM disclosure was associated with UAI, UAIU and HIV testing; and															among men who have sex with men (MSM) in Beirut.		fluent in English or Arabic, residing in greater Beirut, and having had oral or anal sex with a man in the previous 12 months		software.							maintain the majority of the ties in the network, such that the connections between alters (contacts) within a network appear to have a hub or center of concentrated ties.<br>-Condom use self-efficacy was measured using six items adapted from a scale<br>-Alters: Participants were asked to list 20 individuals (or "alters") with whom they have been in communication in the past 6 months<br>-Experience of perceived gay-related discrimination was measured with the subscale of the Multiple Discriminations Scale	demographic variables.	efficacy, relationship status (men in a relationship were more than 3.5 times more likely to have been tested), having more alters (contacts) who know the respondent has sex with men, and greater network centralization (all statistically significant)*.<br><br>-64% of the whole sample reported any unprotected anal sex within the previous 3 months, and of these men, 49 (36% of those who had anal sex) had unprotected sex with an HIV-positive or unknown-status partner.<br><br>-Having unprotected anal sex with partners of unknown HIV status was independently correlated to		(UAI) and HIV testing; lower condom self-efficacy was associated with unprotected anal intercourse with unknown status partners (UAIU) and HIV testing; gay discrimination was associated with UAIU; MSM disclosure was associated with UAI, UAIU and HIV testing; and network centralization was associated with HIV testing.	

Refid	Author	Title	Abstract	User	Level	Author	Date	Jurisd	PubType	PubType_comment	Quant-CaseContr	Quant-CrossSect	Quant-Other	Quant-Other_comment	Qual-FocGroup	Qual-SemiStr	Mixed	Aim	Dates	InclExclCriteria	RecruitmentMethods	DataCollection	Participants	Participants_Youth	Age_mean	Gender_Percentage	Pop_Largest	SampleOther	Outcome	Comparator	RiskFactors	Intervention	MainFindings	Notes
			network centralization was associated with HIV testing. Conclusions: Multi-level social factors influence sexual health in MSM.																												lower condom use self-efficacy, greater gay-related discrimination, and more alters knowing the respondent has sex with men (all statistically significant)*.			
5427	Nahla G Abdel-Tawab, Doaa Oraby, Sally Saher, Sarah Ismail	Understanding HIV-related vulnerabilities and stigma among Egyptian youth		mohammad.alakel	5	Abdel Tawab et tal.	2016	Egypt	PubReport						Qual-FocGroup	Qual-SemiStr		To provide an evidence-based description and interpretation of the HIV-related risks and vulnerabilities faced by different subgroups of youth in Egypt.	Not reported	–key informants and stakeholders who are involved in programs that support youth sexual and reproductive health<br>-male and female young people aged 15–18 years, 18–24 years, and HIV positive male and female youth (i.e. two FGDs per group)<br>-young people belonging to high-risk groups, namely men who inject drugs, women who inject drugs, male and female street children (SC), married adolescent girls	-Male and female young people (15–18 years and 18–24 years) were recruited through local NGOs<br>-Key informants and stakeholders were directly approached by a Population Council Program Officer, who explained the study to them<br>- PWID, MSM, FSWs, MAGs, and male and female SC were all recruited through local NGOs	Interviews and focus group discussion	Not reported. 21 interviews with key informants, 8 focus group discussions, 28 interviews with high risk youth	N= 3733	N=15-24	Not reported	Not reported. Focus group discussions were with young people aged 15–18 years, 18–24 years.	Not reported	Outcomes: the extent to which youth are addressed and involved in national programs around HIV prevention	N/A	The study identified a number of social, economic, cultural, and legal factors that may increase vulnerability of Egyptian youth to HIV. Poverty, unemployment, delayed marriage and gender inequality are examples of factors that increase young people's risk-taking behavior and hence vulnerability to HIV. Knowledge of HIV modes of transmission and condom use are both very low among the Egyptian public. Laws and policies that	N/A	Cultural factors as well as fear of discrimination, harassment, or prosecution prevents at risk young men and women from revealing their high-risk behavior or seeking health care, thereby increasing young people's HIV vulnerability.	

Refid	Author	Title	Abstract	User	Level	Author	Date	Jurisd	PubType	PubType_comment	Quant-CaseContr	Quant-CrossSect	Quant-Other	Quant-Other_comment	Qual-FocGroup	Qual-SemiStr	Mixed	Aim	Dates	InclExclCriteria	RecruitmentMethods	DataCollection	Participants	Participants_Youth	Age_mean	Gender_Percentage	Pop_Largest	SampleOther	Outcome	Comparator	RiskFactors	Intervention	MainFindings	Notes
																				(MAGs), female sex workers (FSWs), and men who have sex with men (MSM).											discriminate against key populations (KPs) (e.g., MSM, PWID, and FSWs) and people living with HIV (PLHIV) may discourage those groups from seeking health care or HIV testing and HIV care and treatment services.<br>-Stigma/Harassment: “The community does not accept discussion of these topics.” Female who injects drugs<br>-Inaccurate information: “If correct information had been available for me, I would not have contracted this disease (HIV).”<br>-Lack of sexual health services or awareness of them among youth.<br>-Cultural assumptions that harm reduction workers are			

Refid	Author	Title	Abstract	User	Level	Author	Date	Jurisd	PubType	PubType_comment	Quant-CaseContr	Quant-CrossSect	Quant-Other	Quant-Other_comment	Qual-FocGroup	Qual-SemiStr	Mixed	Aim	Dates	InclExclCriteria	RecruitmentMethods	DataCollection	Participants	Participants_Youth	Age_mean	Gender_Percentage	Pop_Largest	SampleOther	Outcome	Comparator	RiskFactors	Intervention	MainFindings	Notes
																															doing something against the law/religion resulting in suspended services.			
5457	Frances M Aunon, Glenn J Wagner, Rabih Maher, Danielle Khouri, Rachel L Kaplan, Jacques Mokhbat	An exploratory study of HIV risk behaviors and testing among male sex workers in Beirut, Lebanon		nicole.andruszkiewicz	5	Aunon et al.,	2015	Beirut, Lebanon	PubPeer							Qual-SemiStr		To explore the factors influencing sexual risk behaviors and HIV testing among male sex workers in Beirut.	2011	Male sex workers working in bathhouses (hammam) or as escorts.	Recruited through referrals from an NGO and through participant referrals.	Interviews using a semi structured guide conducted by an outreach worker.	N=16	N=16 (age 19-31)	Mean=19.5; SD=3.9; Median =18; Range = 19-31	100% Male	100% Male Sex Workers	-75% had less than or equal to high school education<br>-87.5% single	(a) disclosure of sexual orientation and sex work, (b) sexual behavior (including condom use) with client and nonclient partners, (c) attitudes and behaviors regarding HIV testing, and (d) perceived norms among male sex workers regarding condom use and HIV testing.	N/A	-Participants reported initiating sex work when they were age 19 years.<br>-The male sex workers from the hammam had significantly more annual sex partners than the escorts, reporting an average of 1,015 unique clients per year (median = 1,095), as compared to an average of 343 annual clients among the escorts (median = 313).<br>-Male sex workers from the hammam reported having riskier sex than the escorts. Only one of the men working at hammam consistently used condoms	N/A	Escorts reported more consistent condom use with clients and HIV testing than hammam MSW, with influential factors including HIV risk knowledge and perceived risk susceptibility, job security, and internalized stigma and related feelings of self-worth and fatalism regarding health and HIV risk. In contrast, both groups of MSW typically opted not to condoms with nonclient sex partners, in an effort to differentiate sex for work versus pleasure.	

Refid	Author	Title	Abstract	User	Level	Author	Date	Jurisd	PubType	PubType_comment	Quant-CaseContr	Quant-CrossSect	Quant-Other	Quant-Other_comment	Qual-FocGroup	Qual-SemiStr	Mixed	Aim	Dates	InclExclCriteria	RecruitmentMethods	DataCollection	Participants	Participants_Youth	Age_mean	Gender_Percentage	Pop_Largest	SampleOther	Outcome	Comparator	RiskFactors	Intervention	MainFindings	Notes
																															with clients.<br>-The majority of the escorts (five of the seven escorts) reported always using condoms with their clients.<br>-Less than one half of the hammam sex workers had ever had an HIV test, and only one reported testing regularly.<br>-All of the escorts had been HIV tested, and one half reported testing regularly.			
5458	Carol Abi Ghanem, Cynthia El Khoury, Matt G Mutchler, Bonnie Ghosh-Dastidar, Susan Kegeles, Elie Balan, Jacques E Mokhbat, Glenn J Wagner	Gay Community Integration as Both a Source of Risk and Resilience for HIV Prevention in Beirut		nicole.andruszkiewicz	5	Ghanem et al.,	2019	Beirut, Lebanon	PubPeer			Quant-CrossSect						To examine the relationships between measures of sexual identity development and HIV protective behaviors (condomless anal sex and HIV testing) among men who have sex with men in Beirut.	07/2016 - 03/2017	Being biologically male and male-identified, age 18 to 29 years, fluent in English or Arabic, residing in greater Beirut, and having had oral or anal sex with a man in the past 12 months.	Long-chain peer referral methods. Recruitment flyers, postings on social media, and word of mouth were added.	Using a survey either self administered or via an interviewer.	N= 218	N= 218	Mean (SD) = 23.8 (3.0)<br>Range = 18 - 29	100% Male	100% MSM	-51.4% attended some university<br>-71.5% earn <$1000 month US	-HIV Protective Behaviors: number of male sex partners in the past three months, how many times they engaged in condomless anal sex t over the past 3 months, how many of those acts involved the use of a condom, and the HIV status of the partners with whom condomless	-Compared any condomless anal sex with male partners whose HIV status was positive or unknown in the past 3 months and any history of HIV testing across different variables.	-Greater integration into the gay community was also the only significant correlate of ever having been tested for HIV*.<br>-Just under half (n = 86; 48.9%) of the 176 men who had anal sex reported any condomless anal sex within the past 3 months,<br>of whom 30 17.0%) had	N/A	-Greater integration into the gay community was significantly associated with having recent condomless anal sex with positive/unknown HIV status partners and having any history of HIV testing, after controlling for covariates.<br>-Peer judgmental communication	

Refid	Author	Title	Abstract	User	Level	Author	Date	Jurisd	PubType	PubType_comment	Quant-CaseContr	Quant-CrossSect	Quant-Other	Quant-Other_comment	Qual-FocGroup	Qual-SemiStr	Mixed	Aim	Dates	InclExclCriteria	RecruitmentMethods	DataCollection	Participants	Participants_Youth	Age_mean	Gender_Percentage	Pop_Largest	SampleOther	Outcome	Comparator	RiskFactors	Intervention	MainFindings	Notes
																													acts were engaged with, history of HIV testing in the past 6 months.		such sex with an HIV-positive (n = 4) or unknown status (n = 26) partner. -Among the potential mediators, those significantly correlated with having had any condomless anal sex in the past 3 months with partners whose HIV status was positive or unknown consisted of greater knowledge of HIV risk, greater perceived judgmentalism in communication about sex, greater number of types of gay-related discrimination experienced, and lower general social support*.		about sex and sexuality-related discrimination were related in bivariate analysis to gay integration and condomless anal sex, but neither were significant correlates of condomless anal sex.	
5463	FHI/MOH Egypt	HIV/AIDS Biological & Behavioral Surveillance Survey: Round Two Summary		mohammad.alakel	5	FHI/MOH	2010	Cairo and Alexandria, Egypt	PubReport			Quant-CrossSect						To track behavioral and biological trends in Most at Risk Populations that influence the hiv	08/ 2010 to 12/ 2010	Not reported	The respondent-driven sampling (RDS) method was used to select the MSM and IDUs. As for	Interviews were conducted with the targeted groups	N= 1951 MSM=791 IDU=560 FSW=200 Street children=400	Not reported	-The median age of 15.0 years for street boys and 14.0 years for street girls.	Street children: 50% split	FSW, street boys, street girls, MSM, Male IDUs	-40% of street girls never attended school -59% of street boys attended primary school	-Ever having sex -Ever injecting drugs; Injecting drugs in the previous 12	Comparing street boys to street girls: street boys were more active than street girls	-Street children reported practicing risk behaviors as many of them were engaged	N/A	-The biological results had three major findings. First the results confirmed the	To decide which groups(i.e street children, MSM) to include.

Refid	Author	Title	Abstract	User	Level	Author	Date	Jurisd	PubType	PubType_comment	Quant-CaseContr	Quant-CrossSect	Quant-Other	Quant-Other_comment	Qual-FocGroup	Qual-SemiStr	Mixed	Aim	Dates	InclExclCriteria	RecruitmentMethods	DataCollection	Participants	Participants_Youth	Age_mean	Gender_Percentage	Pop_Largest	SampleOther	Outcome	Comparator	RiskFactors	Intervention	MainFindings	Notes
		Report, Cairo, Egypt. 2010. Available from: https://www.fhi360.org/sites/default/files/media/documents/BBS%202010_0.pdf																epidemic in Egypt.			the FSWs and Street Children, a non-probability sampling technique was used for selection.				-FSWs with a median age of 27.0 years (range 18-62 years) were interviewed<br>-(median age: MSM Cairo= 22.5 years, MSM Alexandria= 22.5 years and MSM Luxor= 21.0 years). Range=15-59 years<br>-Male IDUs Cairo were slightly older (range: 20 - 64 years, median= 34.0 years) than those in Alexandria (range 20 – 62 years, median= 27.0 years).			-97% of street boys are working to earn money<br>- MSM Cairo 7.4% and 15.6% of MSM Luxor never attended school while almost all MSM Alexandria attended school.	months<br>-Having had commercial sex in the 12 months preceding the survey<br>-Having not used condoms in last sex or past 12 months<br>-Having had forced sex in the past 12 months before the survey<br>-Ever heard of STIs<br>-Ever heard of HIV<br>-Ever got tested for STIs in the 12 months before the survey<br>-Percent who reported consuming alcohol in the four weeks preceding the survey<br>-Percent who reported drug abuse<br>-MSM had oral sex 6 months before the study<br>-Male IDUs reporting to ever selling drugs	as most of them were engaged in income-generating activities especially washing cars and selling small products. It is worth mentioning that 14.9% of street boys and 5.6% of street girls begged on the street while a few of them resorted to steeling (5.2% of boys and 1.1% of girls) and sex work (2.1% of boys and 2.2% of girls) to earn money.<br><br>Comparing MSM in different cities in Egypt: MSM Luxor reported higher proportions of non-injecting drug abuse (97.0%, 95% CI=94.4-99.0%) than MSM Alexandria (70.1%, 95%CI= 61.8-76.4) and the lowest proportions were for MSM	in alcohol consumption, drug abuse and sexual activity.<br>-Alcohol consumption was less practiced than drug abuse with 17.5% of street boys and 12.0% of street girls reported consuming alcohol at least once per week in the 30 days prior to the survey.<br>-68.0% of street boys and 25.9% of street girls reported that they ever tried any type of non-injecting drugs.<br>- 7.0% of street boys and 2.0% of street girls reported injecting drugs in the 12 months preceding the survey<br>-7.7% of sexually active street boys and half of sexually active street girls reported having commercial sex in the 12		concentration of HIV infection in MSM population that was detected in Bio-BSS 2006. Second, they detected the growth and the concentration of the HIV epidemic among male injecting drug users. Third, they identified HIV positive cases among street boys and girls.	

Refid	Author	Title	Abstract	User	Level	Author	Date	Jurisd	PubType	PubType_comment	Quant-CaseContr	Quant-CrossSect	Quant-Other	Quant-Other_comment	Qual-FocGroup	Qual-SemiStr	Mixed	Aim	Dates	InclExclCriteria	RecruitmentMethods	DataCollection	Participants	Participants_Youth	Age_mean	Gender_Percentage	Pop_Largest	SampleOther	Outcome	Comparator	RiskFactors	Intervention	MainFindings	Notes
																														Cairo (48.8%, 95% CI=41.7-58.9%).<br>-MSM Cairo and Alexandria were less indulged in commercial MSM activity and more engaged in non-commercial MSM relations than MSM Luxorin the 6 months prior to the survey.<br>-Unprotected sex appeared to be prevailing among the three MSM groups in both oral and anal sexual activity (table 3.4)<br>-Nearly similar proportions of MSM Cairo (88.3%, 95%CI= 82.7-93.6%) and MSM Alexandria (90.3%, 95%CI= 86.1-94.2%) heard of STIs whereas less than a fifth of MSM Luxor (19.0%, 95%CI= 13.6-24.6%) heard of STIs.	months preceding the survey.<br>- Sexually active street girls did not use condoms in last sex or past 12 months in any type of sexual relations. Sexually active street boys didn't use condoms with commercial sex partners while few of them reported condom use with non-commercial sex partners in last sex (4.1%) and over the 12 months preceding the survey (8.2%).<br>-Many sexually active street children were engaged in non-commercial sexual activity (94.2% of boys and 60.0% of girls).<br>-Among the sexually active street boys in the 12 months preceding the survey, 44.2% reported ever			

Refid	Author	Title	Abstract	User	Level	Author	Date	Jurisd	PubType	PubType_comment	Quant-CaseContr	Quant-CrossSect	Quant-Other	Quant-Other_comment	Qual-FocGroup	Qual-SemiStr	Mixed	Aim	Dates	InclExclCriteria	RecruitmentMethods	DataCollection	Participants	Participants_Youth	Age_mean	Gender_Percentage	Pop_Largest	SampleOther	Outcome	Comparator	RiskFactors	Intervention	MainFindings	Notes
																															having sex with a male partner and 15.0% reported being forced to have sex with male partner.<br>-Less than half street boys and 16.0% of street girls heard of STIs while 69.5% of street boys and 28.5% of street girls heard of HIV.<br><br>-21.5% of FSWs consumed alcohol at least once per week in the month prior to the survey<br>-Over half of FSWs reported ever abusing drugs and 6.0% reported injecting drugs in the 12 months preceding the survey<br>-In the week preceding the interviews, 36.5% of FSWs had non-commercial sex partners. However, only 11.0% out of them used			

Refid	Author	Title	Abstract	User	Level	Author	Date	Jurisd	PubType	PubType_comment	Quant-CaseContr	Quant-CrossSect	Quant-Other	Quant-Other_comment	Qual-FocGroup	Qual-SemiStr	Mixed	Aim	Dates	InclExclCriteria	RecruitmentMethods	DataCollection	Participants	Participants_Youth	Age_mean	Gender_Percentage	Pop_Largest	SampleOther	Outcome	Comparator	RiskFactors	Intervention	MainFindings	Notes
																															condoms the last time they had sex with a non-commercial sex partner and 27.4% used condoms at least once over the past 12 months.<br>-Many FSWs (56.2%) stated that a healthy-looking person can't harbor the HIV infection<br><br>-MSM Luxor reported higher alcohol intake than the other two groups as 27.8% (95% CI=22.3-34.9%) reported alcohol intake every day in the four weeks prior to the survey compared to 4.2% (95% CI=2.0-6.0%) for MSM Alexandria and the least were MSM Cairo with 2.0% (95% CI=0.1-3.6%).<br><br>-79.8% (95%CI=75.0-85.8%) of male IDUs Alexandria reported			

Refid	Author	Title	Abstract	User	Level	Author	Date	Jurisd	PubType	PubType_comment	Quant-CaseContr	Quant-CrossSect	Quant-Other	Quant-Other_comment	Qual-FocGroup	Qual-SemiStr	Mixed	Aim	Dates	InclExclCriteria	RecruitmentMethods	DataCollection	Participants	Participants_Youth	Age_mean	Gender_Percentage	Pop_Largest	SampleOther	Outcome	Comparator	RiskFactors	Intervention	MainFindings	Notes
																															injecting drugs with used needles in the 30 days prior to the survey<br>as compared to 30.7% (95%CI= 22.3-37.4%) of male IDUs Cairo.<br>-40.5% (95%CI= 34.9-48.9%) of male IDUs Alexandria reported sharing needles with at least one person in the 30 days prior to the survey as compared to 22.9 % (95%CI= 16.7-32.4%) of male IDUs Cairo.<br>-Male IDUs in both groups reported MSM activity as 14.3% (95%CI= 5.3-32.3%) of male IDUs Cairo and 7.7% (95% CI=2.9-14.8%) of male IDUs Alexandria reported ever having sex with a male partner.			
5466	L. Kobeissi	Strengthening HIV prevention among most-at-risk populations		mohammad.alakel	5	Kobeissi	2014	Suburbs of Damascus (Rif Damascus), Lattakia and	PubReport			Quant-CrossSect						The specific objectives for this IBBS survey in Syria	11/2013-09/2014	Not reported	The sample was selected using r espondent-driven sampling	The survey tools were based on the FHI-published	A total sample of 400 FSWs, 400 MSM, 400 Prisoners and	Not reported	-FSW: mean age was 29.04 (7.9) with the bulk of the	Not reported	FSWs, MSM, IDUs and Prisoners	-Two cases of HIV positive (0.5%) were found among	-Ever heard of HIV/ AIDS<br>-Have ever tested for	Prisoners: Knowledge of STI related symptoms for	-Only 35.3% of FSW indicated that they have ever tested for	N/A	- The overall knowledge of risk factors was not sufficient	To decide which groups to include as youth.

Refid	Author	Title	Abstract	User	Level	Author	Date	Jurisd	PubType	PubType_comment	Quant-CaseContr	Quant-CrossSect	Quant-Other	Quant-Other_comment	Qual-FocGroup	Qual-SemiStr	Mixed	Aim	Dates	InclExclCriteria	RecruitmentMethods	DataCollection	Participants	Participants_Youth	Age_mean	Gender_Percentage	Pop_Largest	SampleOther	Outcome	Comparator	RiskFactors	Intervention	MainFindings	Notes
		(MARPs) in the Syrian Arab Republic. The Integrated Bio-Behavioral Survey (IBBS) in Syria: 2013-2014. Available from: https://reliefweb.int/sites/reliefweb.int/files/resources/The%20Integrated%20Bio%20Behavioral%20Survey%20%28IBBS%29%20in%20Syria%202013%20-%202014.pdf						Tartous, Syria										revolved around collecting data on:<br>a) Prevalence of HIV, hepatitis B and C among MARPs (Most At Risk Populations)<br>b) Patterns of sexual risk-taking among MARPs,<br>c) Injecting drug use,<br>d) HIV testing among MARPs,<br>e) Self-reported symptoms associated with STIs among MARPs,<br>f) HIV knowledge among MARPs, and<br>g) Stigma toward PLHIV among MARPs.			(RDS). The RDS involved a long chain-referral process (similar to snowball sampling).	manual Behavioral Surveillance Surveys: Guidelines for Repeated Behavioral Surveys in Populations at Risk for HIV. The questionnaires were translated into Arabic,<br>-Data for the survey was collected using face-to-face interviews<br>-blood drawing to analyze for HIV, HBV and HCV	394 prisoners were selected. (total = 1594)		sample clustered between 20-25 years old.<br>-MSM: mean age was 25.2 (+10.9) with the bulk of the sample clustered between 20-34 years old.<br>-IDUs: mean age was 32.1 (+9.9) with the bulk of the sample clustered between 20-24 years old<br>-Prisoners: mean age was 32.8 (+10.9), with the bulk of the sample clustered either between 20-34 years old or older than 40 years of age.<br><br>For the four MARPs, the average age ranged between 20-24 years old, except for prisoners who were slightly older.			the MSMs	HIV/AIDs<br>-Ever heard of HIV/AIDS<br>-Consistent condom use withing the past 6 months	women were slightly better among the prisoners compared to their knowledge of men related symptoms.	HIV/AIDs, among which only 22.8% were voluntarily tested.<br>-Less than 30% of FSW indicated consistent condom use with clients during the past six months, with an average number of sexual partners during the past week 12 of 5.2 (+5.8).<br>-64.8% of FSW indicated that they drink alcohol; among which, 13% indicated that they drink on a daily basis. 11% indicated drug use.<br>-35.8% of FSW indicated that they have been forced to have sex.<br><br>-MSM: 13% indicated condom use during oral sex; 32% indicated using condoms during their last anal sex;<br>Less than 37%		and their overall exposure to health awareness campaigns to HIV/AIDS was limited.<br>-The data reflects, as well, that these risk groups are more likely to engage in multiple sexual activities with both usual and casual partners. They are characterized by significant low use of condoms. The majority indicated drinking alcohol and a high percentage actually reported injecting drug use (ranging between 11-22%). Injection drug use was also characterized to be combined with other medications like sleeping pills and Valium. Hence, the four groups are heavily engaged in high-risk	

Refid	Author	Title	Abstract	User	Level	Author	Date	Jurisd	PubType	PubType_comment	Quant-CaseContr	Quant-CrossSect	Quant-Other	Quant-Other_comment	Qual-FocGroup	Qual-SemiStr	Mixed	Aim	Dates	InclExclCriteria	RecruitmentMethods	DataCollection	Participants	Participants_Youth	Age_mean	Gender_Percentage	Pop_Largest	SampleOther	Outcome	Comparator	RiskFactors	Intervention	MainFindings	Notes
																															indicated consistent condom use partners during the past six months, with an average number of sexual partners whom practiced with oral sex during the past six months was 45.7(+189.2); and whom practiced with anal sex was 26.4 (+127.8)<br>-67.5% of MSM indicated drinking alcohol, among which 11% indicated that they drink on daily basis. 12.3% indicated drug use.<br>-Only 43.4% of IDUs indicated that they have ever tested for HIV/AIDs<br>-Less than 30% of IDUs indicated consistent condom use with clients during the past six months, with an average number of sexual partners during the past		behaviors that will further exacerbate their risk of contracting HIV/AIDs as well other STDs.	

Refid	Author	Title	Abstract	User	Level	Author	Date	Jurisd	PubType	PubType_comment	Quant-CaseContr	Quant-CrossSect	Quant-Other	Quant-Other_comment	Qual-FocGroup	Qual-SemiStr	Mixed	Aim	Dates	InclExclCriteria	RecruitmentMethods	DataCollection	Participants	Participants_Youth	Age_mean	Gender_Percentage	Pop_Largest	SampleOther	Outcome	Comparator	RiskFactors	Intervention	MainFindings	Notes
																															week of 5.2 (+ 5.8). 93.9% indicated that they drink alcohol, among which 29% indicated that they drink on daily basis and 56.8% indicated that they drink more than once per week. 31.1% indicated using drugs more than once a day.<br><br>-IDUs; 19.5% indicated that they have used a syringe used by someone else the last time they injected, and 11.2% reported using a syringe used by someone else during the last month. 38% cleaned the used syringes with either alcohol or water. Only 20.3% indicated receiving management/ rehabilitation for their addiction (current or past).			

Refid	Author	Title	Abstract	User	Level	Author	Date	Jurisd	PubType	PubType_comment	Quant-CaseContr	Quant-CrossSect	Quant-Other	Quant-Other_comment	Qual-FocGroup	Qual-SemiStr	Mixed	Aim	Dates	InclExclCriteria	RecruitmentMethods	DataCollection	Participants	Participants_Youth	Age_mean	Gender_Percentage	Pop_Largest	SampleOther	Outcome	Comparator	RiskFactors	Intervention	MainFindings	Notes
																															-Only 40% indicated that they have ever tested for HIV/AIDS,<br><br>-Prisoners: 25.8% indicated using condoms during their last sexual act; 4.3% indicated consistent condom use with casual partners during the past six months. 21% indicated that they get paid to have sex with a casual partner. 46.3% indicated drinking alcohol, among which 17.3% indicated that they more than once per week. 22%indicated drug use.			
5467	Kingdom of Morocco,Ministry of Health,Department of Epidemiology and Disease Control,National STI/AIDS Programme	HIV modes of transmission analysis in Morocco. Available from: http://files.unaids.org/en/media/unaids/contentassets/documents/countryreport/2010/201008_M		mohammad.alakel	5	Mumtaz	2010	Morocco	PubReport			Quant-CrossSect						Not reported.	Not reported	Youth aged 15-24.	Not reported	Not reported.	2000	2000	Range 15-24 years	Not reported	Youth	Not reported	-ever having vaginal intercourse<br>-ever having anal intercourse<br>-age at first sexual act<br>-ever having sex with a FSW<br>-number of condoms used	Not applicable.	- 36% of males and 5.2% of females declared having had a vaginal intercourse ever (total for both males and females is 20.6%). Also 29.3% of males	Not applicable.	Youth in Morocco report relatively high levels of risk behavior.	Has one paragraph (page 16) that focuses on another study that we cannot access which the above data is from .

Refid	Author	Title	Abstract	User	Level	Author	Date	Jurisd	PubType	PubType_comment	Quant-CaseContr	Quant-CrossSect	Quant-Other	Quant-Other_comment	Qual-FocGroup	Qual-SemiStr	Mixed	Aim	Dates	InclExclCriteria	RecruitmentMethods	DataCollection	Participants	Participants_Youth	Age_mean	Gender_Percentage	Pop_Largest	SampleOther	Outcome	Comparator	RiskFactors	Intervention	MainFindings	Notes
		OT_Morocco_en.pdf																											in the last month<br>-using a condom during last sex act<br>-ever used drugs		and 1.0% of females reported ever having anal sex (total 15.2%), and as much as 3.7% of males reported ever having anal sex with a male.<br>-The average age at first sexual act was 16.7 years for males and 18.2 years for females.<br>-Among the sexually active participants, 6.9% of males 11.3% of females declared having sexual acts regularly since puberty, 89.2% of males and 49.3% of females sometimes or from time to time, and 3.9% of males and 23.9% of females very rarely.<br>-Also, since sexual debut, 68% of males and 38.5% of females had more than one sexual partner			

Refid	Author	Title	Abstract	User	Level	Author	Date	Jurisd	PubType	PubType_comment	Quant-CaseContr	Quant-CrossSect	Quant-Other	Quant-Other_comment	Qual-FocGroup	Qual-SemiStr	Mixed	Aim	Dates	InclExclCriteria	RecruitmentMethods	DataCollection	Participants	Participants_Youth	Age_mean	Gender_Percentage	Pop_Largest	SampleOther	Outcome	Comparator	RiskFactors	Intervention	MainFindings	Notes
																															(total 34.6%).<br>- 67% of sexually active male youth declared ever having sex with a FSW<br>-During the last sex with a FSW, 65.3% of the youth used a condom.<br>-Overall during last sex, 71% of males and 62.5% of females used condoms (total 70.4%).<br>-During the last month, males used on average 1.5 condoms and females 2.0 condoms (total 1.5 condoms).<br>- On the other hand, 16.4% of males and 1.8% of females declared current use of any type of drugs.			
